# Minor tranquillizers for short-term treatment of newly onset symptoms of anxiety and distress: a systematic review with network meta-analysis of randomized trials

**DOI:** 10.1007/s00406-023-01680-0

**Published:** 2023-08-25

**Authors:** Klaus Munkholm, Anja Ussing, Maria Brink, Henriette Edemann-Callesen, Sengül Sari Canbolat, Robin Christensen, Kristine Søgaard Dahl, Bjørn H. Ebdrup, Mikkel Erik Juul Jensen, Casper Kierulf-Lassen, Gitte Krogh Madsen, Sabrina Mai Nielsen, Camilla Paludan Paulsen, Jeanett Friis Rohde, Simon Tarp, Lone Baandrup

**Affiliations:** 1 Mental Health Services in the Capital Region of Denmark, Mental Health Centre Copenhagen, Copenhagen, Denmark; 2 Danish Health Authority, Copenhagen, Denmark; 3 Department of Psychiatry Odense, Mental Health Services in the Region of Southern Denmark, Odense, Denmark; 4 Section for Biostatistics and Evidence-Based Research, The Parker Institute, Bispebjerg and Frederiksberg Hospital, Copenhagen, Denmark; 5 Research Unit of Rheumatology, Department of Clinical Research, University of Southern Denmark, Odense University Hospital, Odense, Denmark; 6 Mental Health Services in the Capital Region of Denmark, Mental Health Centre Sct. Hans, Roskilde, Denmark; 7 Center for Neuropsychiatric Schizophrenia Research (CNSR), Mental Health Centre Glostrup, University of Copenhagen, Glostrup, Denmark; 8 Faculty of Health and Medical Sciences, Department of Clinical Medicine, University of Copenhagen, Copenhagen, Denmark; 9 Department of Geriatric Medicine, Aarhus University Hospital, Aarhus, Denmark; 10 Department of Clinical Pharmacology, Aarhus University Hospital, Aarhus, Denmark; 11 General Practice, Roskilde Laegehus, Roskilde, Denmark; 12 Research Unit for Dietary Studies, The Parker Institute, Bispebjerg and Frederiksberg Hospital, Frederiksberg, Denmark; 13 Mental Health Centre Copenhagen, Hovedvejen 17, 1st Floor, 2000 Frederiksberg, Denmark

**Keywords:** Minor tranquilizers, Anxiety, Distress, Systematic review, Network meta-analysis.

## Abstract

Unspecific symptoms of anxiety and distress are frequently encountered in patients in both general practice and acute psychiatric services. Minor tranquillizers may be a treatment option when non-pharmacological interventions are insufficient or unavailable. We conducted a systematic review with network meta-analysis of the evidence for short-term (1–4 weeks) pharmacological treatment of newly onset symptoms of anxiety and distress. We searched the PsycInfo, MEDLINE, EMBASE and Cochrane Library databases and extracted data following a predefined hierarchy of outcomes. We assessed risk of bias using the Cochrane Risk of Bias tool and the certainty of the evidence using the Grading of Recommendations Assessment, Development and Evaluation framework (GRADE). We included 34 randomized trials comprising a total of 7044 patients with adjustment disorders or anxiety spectrum disorders. The network meta-analysis showed that regarding the critical outcome symptoms of anxiety within 1–4 weeks benzodiazepines (SMD − 0.58, 95% CI − 0.77 to − 0.40), quetiapine (SMD − 0.51, 95% CI − 0.90 to − 0.13) and pregabalin (SMD − 0.58, 95% CI − 0.87 to − 0.28) all performed better than placebo with no statistically significant difference between the drugs. Data on other important outcomes were inconsistently reported. Adverse effects varied, but overall, it was uncertain whether adverse effects differed between interventions. The evidence regarding the risk of dependence was uncertain, but dependence may be a concern in susceptible individuals even with short-term treatment. Overall, the certainty of the evidence according to GRADE was rated as low to very low across outcomes. Despite the limitations in the evidence, the results of this review can inform treatment guidelines, supporting clinicians in the choice of minor tranquillizer in this prevalent and help-seeking, clinically heterogeneous population.

**Supplementary Information:**

The online version contains supplementary material available at 10.1007/s00406-023-01680-0.

## Introduction

For the last couple of decades, a focus on interventions to reduce prescribing of benzodiazepines has led to a decline in the proportion of people treated with benzodiazepines as well as a decline in the number of long-term benzodiazepine users [27, 28, 32, 67]. However, this picture is not uniform and reports of high or increasing prevalence of benzodiazepine use are still prevalent [33, 59, 71, 78]. Administrative limitations to reduce benzodiazepine prescribing practice have been particularly pronounced in Denmark compared with other comparable countries[27]. Consequently, prescribing of long-acting benzodiazepines was reduced by 66% and prescribing of short-acting benzodiazepines by 37% in Denmark from 2003 to 2013 [18]. The number of short-term users declined from 27.3 to 23.4 per 1000 inhabitants from 2012 to 2017 and the number of long-term users declined from 7.7 to 5.3 per 1000 inhabitants in the same period [11]. This coincided with the introduction of a revised national guideline on prescription of addictive drugs in 2008 [13]. Meanwhile, national audits from the Danish Health Data Authority show a subsequent increase in the use of other sedative drugs such as low-dose quetiapine (≤ 150 mg daily), antihistamines, and melatonin [10], with use of low-dose quetiapine having tripled within the last decade [12]. Other countries have observed similar changes in the prescribing patterns [33]. There has been an increasing concern regarding this shift from benzodiazepine prescribing to off-label use of other drugs [33] and it has been claimed that the restrictive approach towards benzodiazepines presumably underlying this change in prescription practice has been taken too far [29].

Benzodiazepines have a rapid anxiolytic effect but pose a potential risk of development of tolerance and dependency [62]. Patterns of use in Europe, Canada and the US show substantially higher use of benzodiazepines in women compared with men and that the use increases with age [47]. Recent data show that on average, 39.4% of first-time benzodiazepine users become long-term users with certain benzodiazepines posing an even higher risk [68]. Other sedative drugs used in clinical practice (e.g., antihistamines, sedative antidepressants) represent other caveats of prescribing such as anticholinergic, neurological, and metabolic side effects. Especially use of low-dose quetiapine as a substitute to avoid benzodiazepine prescribing has been a concern [50] with data indicating that low-dose quetiapine prescriptions are most often initiated in general practice, especially in the elderly [25] and most often as off-label prescriptions [6]. Antipsychotics are increasingly used off-label and acute stress and adjustment disorders with related symptoms of anxiety and distress are among the most frequent indications (12), even though knowledge of the risk–benefit ratio for off-label indications is missing.

There is a paucity of knowledge on the best choice of minor tranquilizer for the short-term treatment of newly onset symptoms of anxiety and distress that cannot be managed by non-pharmacological approaches. Therefore, the Danish National Health Authority gathered a guideline development group to examine the evidence-base and to establish national clinical recommendations. National clinical recommendations from the Danish Health Authority are developed in a standardized way following the Cochrane Handbook [22] and the GRADE Handbook [57].

This paper presents the results of the systematic review with network meta-analysis that was conducted to establish the evidence-base for developing the national clinical recommendation on the short-term pharmacological treatment to manage newly onset symptoms of anxiety and distress in non-hospitalised adults. Patients suffering from these symptoms mostly present in general practice but may also present to acute psychiatric services. The anticipated duration of pharmacological treatment should ideally be short, i.e., a maximum of 4 weeks, and patients with evident need for longer-term treatment are not addressed in this review. The patients typically present with symptoms resembling adjustment disorder after distressing life events, but acute worsening of longer lasting underlying symptoms (e.g., in patients with mild to moderate depression or anxiety) are also covered by the population of interest for this review. To fit with the inclusion criteria of this review, non-pharmacological interventions (e.g., general counselling, sleep-advice and psychoeducation) should have been tried and found insufficient or they have been deemed not relevant for the given patient population.

## Methods

The systematic review was conducted and reported in accordance with the Cochrane Handbook [22], the Preferred Reporting Items for Systematic Reviews and Meta-analyses (PRISMA) recommendations [48] and PRISMA recommendations for Network Meta-analysis [26]. The systematic review was conducted as part of the development of a Danish national clinical recommendation on the use of minor tranquilizers for short-term symptom relief of newly onset symptoms of anxiety and distress in adults published by the Danish Health Authorities in 2023 [8]. The systematic review question including detailed inclusion and exclusion criteria regarding populations, interventions, comparators and outcomes was pre-specified in a protocol prior to the performance of the literature search. The protocol was approved by the management of the Danish Health Authority and published on the official website of the Danish Health Authority [9] prior to initiating the review.

### Eligibility criteria

Our pre-specified eligibility criteria were based on the Population, Intervention, Comparison, Outcome and Time (PICOT) framework [20, 70]. We considered randomised clinical trials (RCTs) which investigated the effect of minor tranquilizers for short-term symptom relief of newly onset symptoms of anxiety and distress in adults. Both published and unpublished trials were eligible as long as relevant results were available.

#### Population

Non-hospitalised adults with newly onset symptoms of anxiety and distress in need of short-term pharmacological treatment (maximum up to 4 weeks). This could include patients who were in distress or crisis as a result of illness, death, accident or other stressful life-events and could include patients with a diagnosis of acute stress or adjustment disorder. It includes both patients without prior psychiatric disorder as well as patients diagnosed with mild to moderate depression or anxiety disorder. The population does not include patients diagnosed with organic mental disorders, psychotic disorders, bipolar disorder, severe depression, obsessive compulsive disorder, or otherwise in need of inpatient mental health care. The need for short-term pharmacological treatment was defined as a need for pharmacological treatment due to symptoms of anxiety or distress that affect the patient to such a degree that non-pharmacological treatment was not considered relevant or non-pharmacological treatment had been tried without sufficient effect.

#### Interventions

We included trials investigating one or more of the following interventions: benzodiazepines, antipsychotics with sedative effects (e.g., quetiapine or olanzapine in low doses), sedative antidepressants (mirtazapine and mianserin), antihistamines with sedative properties (e.g., promethazine), melatonin, z-drugs (zopiclone, zolpidem) and pregabalin. The interventions could be administered as monotherapy or in combination with any other psychopharmacologic- or non-pharmacologic treatment. For the network meta-analysis, we included arms with drugs other than the above, but only to inform the indirect comparisons in the network. There were deviations from our protocol with regards to the drugs included (see Supplement).

#### Comparators

Comparators included both no pharmacological treatment and the total group of drugs included as interventions.

#### Outcomes

Outcomes were in advance categorized as critical or important to patients [20, 70].

Critical (primary) outcomes were: symptoms of anxiety measured with the Hamilton Rating scale for Anxiety (HAM-A), function of daily living/disability (measured on any scale), and serious adverse events. As no valid minimal clinically important difference (MCID) has been established for the HAM-A, the guideline working group decided to consider a standardised mean difference (SMD) on the HAM-A of 0.3 as the MCID for symptoms of anxiety. We did not consider any specific MCID for other outcomes.

Secondary outcomes were: quality of life, suicidal thoughts/attempts, addiction, fractures, weight change, cardiac side effects, extrapyramidal symptoms, sleep quality, daytime drowsiness, and dizziness.

#### Time points of interest

Trials reporting data within 4 weeks after commencement of pharmacological treatment for one of our critical outcomes of interest (symptoms of anxiety or function of daily living/disability) were eligible for inclusion. Outcomes reported at 1 week after commencement were prioritized, followed by outcomes reported at 2, 3 and 4 weeks, respectively, where these were available. For all other outcomes, this timeframe was the primary time point of interest, except for the outcomes addiction and suicidal thoughts/attempts, for which the time point of interest was extended to 6 and 12 months, respectively.

### Data sources and search strategy

First, we conducted a broad systematic search for systematic reviews to identify RCTs that met our inclusion criteria. The search was conducted on 14th January 2022 and limited from 1st January 2016 and forward. From this search, we included a systematic review that investigated pharmacological treatment for adjustment disorder in adults [46]. Since the body of evidence that could be included from this review was very sparse and only included data for two of our interventions of interest, we decided to expand our inclusion criteria to include indirect evidence from trials including adults with a diagnosis of anxiety without recent onset of symptoms of anxiety. Following this, another three systematic reviews investigating the effect of pharmacological treatment on social anxiety [77], generalised anxiety disorder [60] and panic disorder [5], respectively, were included. We screened all included RCTs in the included systematic reviews for eligibility. We then conducted a systematic search for primary trials. The search was conducted on 11th February 2022 and was limited to the last search in the included systematic reviews (January 2015). This search was updated 8th September 2022. Searches were conducted in PsycInfo, PubMed/MEDLINE, EMBASE and Cochrane Library. No restrictions concerning publication status were applied. Language was limited to English, Norwegian, Swedish and Danish. The searches were supplemented by manually screening the reference lists in the reports of the included trials and in relevant systematic reviews. The details of the search strategy and the results are available in the Supplement.

### Trial selection

After removal of duplicates the identified references were imported to Covidence systematic review software (Veritas Health Innovation, Melbourne, Australia) for screening and risk of bias assessments. One of two review authors screened title and abstracts for eligibility (HEC or KM), and full-text reports were screened independently and in duplicate by two review authors (KM, HEC, TK, MEJJ, CKL or AU). Disagreements were resolved through discussion by three review authors (KM, AU and ST).

### Data extraction and quality assessments

Two authors independently extracted data in Excel (AU, JFR, SSC or CPP). A template including information on primary diagnosis, interventions, comparators, outcome measures and results was created and tested a priori. Data extraction was validated and any discrepancies were resolved through discussion. The quality of the included systematic reviews was assessed by two independent reviewers (KM and AU) with A MeaSurement Tool to Assess systematic Reviews (AMSTAR) [58]. The risk of bias (RoB) of the primary trials included from the systematic review of O’Donnell et al. was assessed independently in Covidence by two reviewers (AU and JFR) using the Cochrane Risk of Bias tool [21]. Consensus was reached through discussion. The risk of bias assessment of the primary trials included from the three other systematic reviews [5, 60, 77] was adapted from the source.

### Certainty of the evidence

For the results of the meta-analyses, the certainty of the estimate for each outcome was assessed using the GRADE framework [57]. Here, the certainty in the effect estimates obtained by data from RCTs starts at “high certainty” and can be downgraded one or more times to “moderate”, “low”, or “very low” certainty based on limitations in trial design (risk of bias), indirectness, imprecision, inconsistency, and publication bias. The overall certainty of the body of evidence is determined by the lowest certainty level for the critical outcomes.

For the results of the network meta-analysis, the confidence was assessed using CINeMA [44]. Here, confidence in the results is assessed based on six domains: within-study bias, reporting bias, indirectness, imprecision, heterogeneity, and incoherence.

All assessments of certainty in the estimates were made in collaboration between three review authors (KM, AU and ST) and for the confidence in the NMA estimates SMN participated as well. All assessments were finally approved by the guideline panel.

### Statistical analyses/methods

For all analyses, trials testing the same drug in multiple arms (but in different doses), were handled by pooling those arms. Thus, individual trials contribute only a single arm for a specific treatment.

#### Pair-wise meta-analyses

Meta-analyses were performed as a random-effects model as variation between studies was anticipated. Missing values for standard deviations (SD) were calculated from the available data when possible, otherwise missing data were imputed with the median SD from the trials reporting an exact SD. For all continuous outcomes, standardised mean differences (SMD) were calculated and for dichotomous outcomes Risk Ratios (RR) were calculated. For all estimates, 95% confidence intervals (CIs) were provided. The absolute effect estimates per 1000 individuals and corresponding CIs were calculated in MAGICapp [39] based on the assumed risk in the control group and the estimated risk ratio. When an absolute effect estimate could not be calculated, e.g., due to no events for a dichotomous outcome, the absolute effect was based on a risk difference analysis. Heterogeneity was assessed by visual inspection of the forest plot, and by interpreting the *I*^2^ statistic and Chi^2^-test. To explore reasons for potential heterogeneity, we conducted a subgroup analysis dividing the interventions into subgroups of different drugs within the same drug class. We generated funnel plots to judge publication bias when at least ten trials were included in an analysis. All pair-wise meta-analyses were performed using Review Manager (The Nordic Cochrane Centre, Cochrane Collaboration, Copenhagen, Denmark).

#### Network meta-analysis

For the critical outcome, anxiety symptoms measured with HAM-A, we performed a random-effects network meta-analysis. We estimated the SMD with corresponding 95% CIs for each treatment comparison, and estimated the p-scores [55]. For the CINeMA assessments (incl. the domains within-study bias, reporting bias, indirectness, imprecision, heterogeneity, and incoherence), we decided to only evaluate the comparisons between agomelatine, benzodiazepines, hydroxyzine, mianserin, pregabalin, quetiapine, and placebo. Within-study bias was evaluated based on average RoB for each comparison. Reporting bias was evaluated based on the average risk of reporting bias for each comparison. Indirectness was evaluated based on average indirectness for each comparison. Imprecision, heterogeneity, and incoherence were evaluated considering a clinically important size of effect of 0.3 SMD. The transitivity assumption underlying network meta-analysis (i.e., that the network includes studies that are sufficiently similar in important clinical and methodological characteristics) was evaluated by comparing the distribution of clinical and methodological variables that could act as effect modifiers across treatment comparisons, as well as conceptually evaluating the definition of each node (treatment) in the network, and that the treatments have similar indications (i.e., are in principle jointly randomizable). The variables evaluated were diagnosis, use of co-medication and the use of a placebo run-in design. We evaluated the consistency (i.e., the agreement between direct and indirect evidence; sometimes called coherence) by considering direct and indirect evidence separately with node splitting (also called side-splitting). For sensitivity, we repeated the network meta-analysis with mean differences instead of SMDs, as well as performed a Bayesian random-effects network meta-analysis using Markov–chain Monte–Carlo simulation with vague priors. We evaluated the ranking probabilities and calculated the surface under the cumulative ranking curves (SUCRA). We assessed the convergence based on trace plots and the Gelman-Rubin statistic. The network meta-analyses were performed in R version 4.0.3 (R Core Team, Vienna, Austria) with the *netmeta* [54] and *gemtc* [72] packages, of which the latter performs network meta-analyses within the Bayesian framework using JAGS (i.e., a program for analysis of Bayesian hierarchical models using Markov-chain Monte-Carlo simulation). The *pcnetmeta* [37] package was used to draw the network plots. For the CINeMA assessment, we used the CINeMA software [44].

## Results

### Selection of studies

In the search for systematic reviews, we identified 2,828 records after removal of duplicates. We excluded 2,735 records by screening of title and abstracts and assessed 93 records for inclusion by full-text review. We initially included one systematic review concerning pharmacological treatment of patients with adjustment disorder [46], and in our supplementary search included a further three reviews, which concerned pharmacological treatment of patients with generalized anxiety disorder [60], patients with social anxiety disorder [77], and patients with panic disorder [5]. From these systematic reviews, we included a total of 30 primary RCTs that met our adjusted inclusion criteria.

In the search for primary studies (performed 11 February 2022 and updated 8 September 2022), we identified 3,419 records after removal of duplicates of which 28 were assessed for inclusion by full-text review and four RCTs were included.

In total, 34 RCTs (43 publications and 1 unpublished trial) comprising a total of 7044 patients were included. See PRISMA flowchart of the trial selection process (Supplementary Fig. S1A, B). A table of included trials are listed in Supplementary Table S1. A complete list of excluded trials assessed in full-text with reasons for exclusion is given in Supplementary Table S2.

### Quality assessment of systematic reviews

The AMSTAR evaluation of the included systematic reviews is presented in Supplementary Table S3. Concerning the rigor and transparency of the literature search and inclusion of primary trials (domain 1–4), we judged that the reviews were of sufficient quality to enable us to base our search for primary trials on their last search date.

### Trial characteristics

The characteristics of the included trials are available in Supplementary Table S1. The populations included patients with adjustment disorder in 6 trials [3, 14, 15, 43, 51, 63], patients with generalized anxiety disorder in 14 trials [19, 30, 35, 38, 40–42, 49, 51–53, 61, 64, 66], patients with social anxiety disorder in 5 trials [36, 74–76], and patients with panic disorder in 9 trials [1, 2, 4, 16, 31, 34, 45, 56, 69].

All trials included participants of both sexes, except for the trial by Ravazzi et al., who included a population of women with breast cancer [51]. In the vast majority of trials, there was a preponderance of women and the trials involved patients with an average age between 33 and 57 years. The majority of trials included only outpatients [3, 4, 14, 16, 19, 30, 35, 38, 40, 42, 43, 45, 49, 52, 53, 56, 63, 64, 66, 69, 73, 74]. In one trial, the population consisted of both inpatients and outpatients [1], in two trials the patients were inpatients [34, 61] and in eight trials, there was no information on hospitalization status [2, 15, 31, 35, 41, 66, 75, 76].

In the vast majority of trials, there was no information on whether the symptoms of anxiety were newly onset, and in the trials that had information on duration, the patients had suffered from symptoms for at least 1 year [2, 16, 31, 34, 36, 45, 53, 56, 64, 66, 73, 74] except in the trial by Ansseau et al. [3], where the patients had experienced symptoms for an average of 2 months.

None of the trials had information on whether non-pharmacological treatment had been considered or tested.

In most trials, patients with psychotic disorders [1, 2, 16, 30, 31, 34, 36, 38, 40, 42, 45, 51–53, 56, 63, 64, 66, 73–76], depression [2, 15, 16, 19, 30, 34, 36, 38, 42, 43, 45, 49, 51, 53, 56, 61, 63, 64, 73, 74] and/or bipolar disorders [1, 31, 34, 36, 42, 45, 51–53, 56, 63, 64, 66, 69, 73–75] were excluded, just as known alcohol or substance use disorder was often an exclusion criterion [1, 2, 15, 19, 30, 31, 36, 38, 40, 42, 45, 52, 53, 56, 61, 63, 64, 66, 69, 73–76].

The experimental interventions applied in the trials included: benzodiazepines (alprazolam, lormetazepam, clorazepate, lorazepam, bromazepam, diazepam and delorazepam) [1, 3, 14, 15, 19, 34, 38, 41–43, 45, 49, 51–53, 56, 61, 63, 66, 69, 75], tricyclic antidepressants (TCA) (imipramine, clomipramine, maprotiline and opipramol) [1, 2, 4, 16, 31, 34, 42, 53, 56, 69], monoamine oxidase inhibitors (MAO-I) (brofaromine, moclobemide and phenelzine) [4, 31, 36, 73, 76], selective serotonin inhibitors (SSRI) (fluoxetine, fluvoxamine, escitalopram and paroxetine) [2, 16, 40, 53], mianserin [3], tianeptine [3], pregabalin [19, 49, 52, 66], quetiapine [30, 35, 40], anxiolytics (etifoxine and buspirone) [43, 63, 74], beta blockers (atenolol) [36], antihistamines (hydroxyzine) [38], and agomelatine [64, 65]. In 23 of the trials, a control arm that received a placebo was included [1, 14, 19, 30, 35, 36, 38, 40–42, 45, 49, 52, 56, 61, 64–66, 69, 73–76]. In one trial, the interventions (benzodiazepines, homeopathic medicines and placebo) were given in addition to paroxetine [61], and in another trial, quetiapine or placebo was given in addition to an unspecified SSRI or SNRI [30].

The trials had a duration of between 4 and 24 weeks. Data regarding the critical outcome of symptoms of anxiety were assessed in the short-term (1–4 weeks) in all the included trials and in one trial for the critical outcome of daily functioning.

In 19 of the included trials industry funding was indicated [1, 3, 15, 19, 30, 31, 35, 36, 38, 40, 41, 43, 45, 51, 52, 56, 63, 64, 69], 1 trial did not report industry funding [61] and for the remaining 14 trials, sponsor information was not reported.

### Risk of bias in the included trials

The summary of the risk of bias assessment is available in Supplementary Fig. S2. We judged six trials to be at low risk of selection bias [40–43, 51, 52] and the remaining 28 trials were at unclear risk of selection bias due to inadequate reporting regarding random sequence generation and/or allocation concealment. Three trials were judged to be at high risk of performance bias due to lack of blinding of participants and personnel [14, 53, 61], 15 trials were judged to be at low risk [17, 19, 30, 35, 38, 40–43, 49, 52, 56, 63–65] and the remaining 16 trials were judged to be at unclear risk of performance bias. Two were judged to be at high risk of detection bias due to lack of blinding of outcome assessors [14, 53], while 12 trials were at low risk [17, 19, 38, 40–43, 49, 52, 56, 61, 65] and the remaining 20 trials were judged to be at unclear risk of detection bias. Sixteen trials were judged to be at high risk of attrition bias due to incomplete outcome data [3, 16, 17, 19, 30, 35, 36, 38, 40–42, 49, 51–53, 69], while 10 trials were judged to be at low risk [15, 31, 43, 45, 56, 65, 73–76], and the remaining 8 trials were judged to be at unclear risk of attrition bias. Five trials were assessed to have high risk of bias due to selective reporting [2, 4, 7, 16, 69], while 11 trials were judged to be at low risk [3, 30, 31, 34, 35, 43, 45, 51, 63, 64], and the remaining 18 trials were judged to be at unclear risk of reporting bias.

### Certainty of the evidence

The results of the GRADE-process and the CINeMA assessment are shown in Supplementary Table S3.

The overall certainty of evidence was low to very low. The most common reasons for downgrading were indirect study populations, imprecise meta-analysis summary effect estimates, and risk of bias.

Since the funnel plots did not suggest publication bias, no downgrading for this item was performed (not shown).

### Quantitative synthesis

We present the results of the network meta-analysis of the critical outcome anxiety symptoms below. The results for the pair-wise meta-analyses of all outcomes, including forest plots, are presented in the Supplement (Supplementary Figs. S3–S5 and Supplementary Results text, Supplementary Table S8). Summary of Findings (SoF) tables are available in Supplementary Table S5.

### Network meta-analysis

**Fig. 1:**
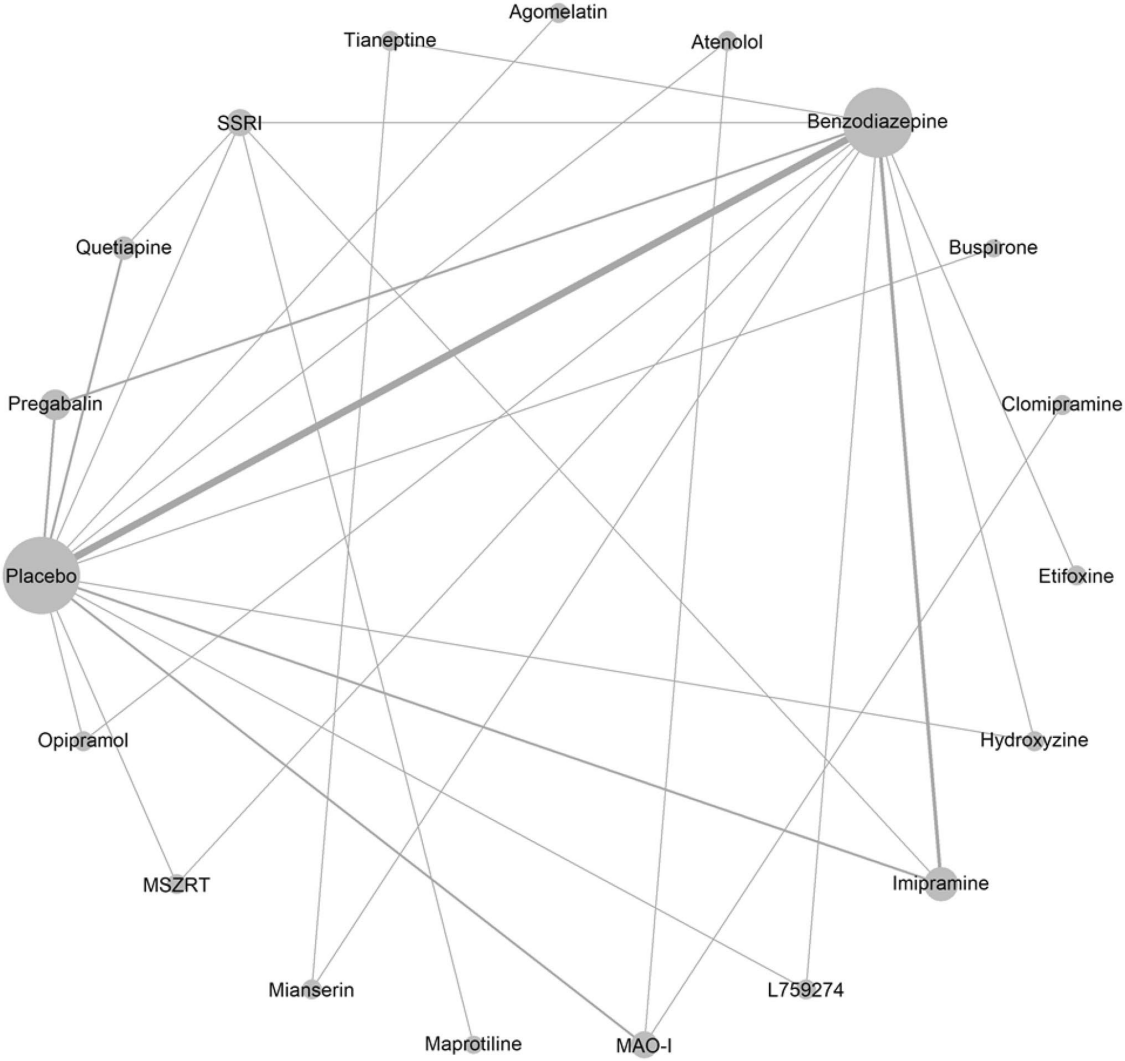
Network meta-analysis network plot of anxiety symptoms measured with HAM-A. The circle size reflects the number of trials, and the line width reflects the number of comparisons. No connecting line between two treatments indicates that there is no direct comparison. *HAM-A* Hamilton Anxiety Scale

**Fig. 2:**
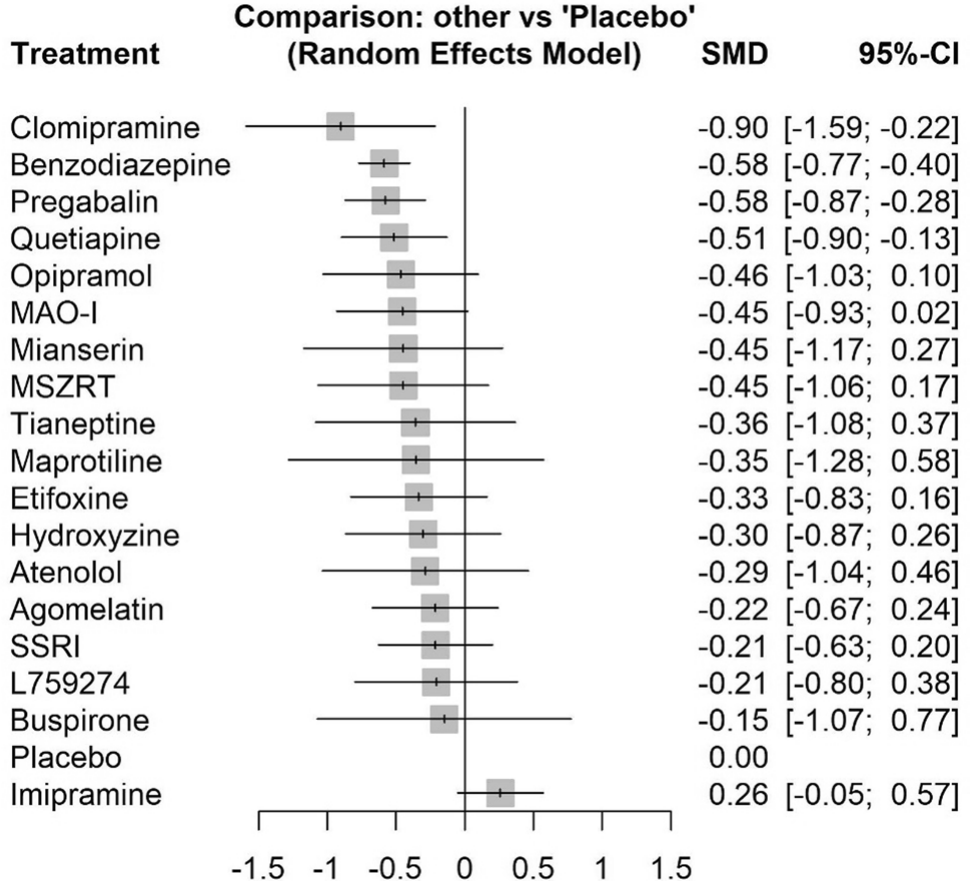
Forest plot of the effect of each treatment against placebo on anxiety symptoms measured with HAM-A (SMDs with 95% CIs), estimated from network meta-analysis. *HAM-A* Hamilton Anxiety Scale, *SMD* standardized mean difference, *CI* confidence interval, *MAO-I* monoamine oxidase inhibitors, *MSZRT* modified suanzaorentang, *SSRI* selective serotonin reuptake inhibitors

The network meta-analysis included 31 trials and is presented in Fig. 1. Among the treatments of interest, benzodiazepines (SMD − 0.58, 95% CI − 0.77 to − 0.40, low certainty evidence), pregabalin (SMD − 0.58, 95% CI − 0.87 to − 0.28, low certainty evidence), and quetiapine (SMD − 0.51, 95% CI − 0.90 to − 0.13, low certainty evidence), reduced symptoms of anxiety measured on the HAM-A scale compared with placebo (Table 1, Fig. 2).There were no statistically significant differences between benzodiazepines, pregabalin, and quetiapine in the reduction of symptoms of anxiety (Table 1). The p-scores indicated that benzodiazepines ranked the highest and that quetiapine ranked the lowest of the three drugs or drug groups (Supplementary Fig. S4A, B).

**Table 1:**
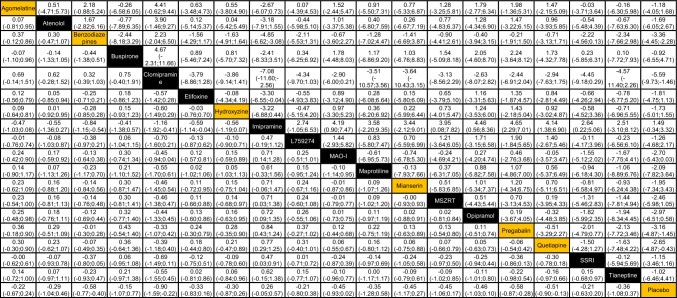
Results for all treatment comparisons of the network meta-analysis of anxiety symptoms measured with HAM-A (SMDs with 95% CIs bottom left side, MDs with 95% CIs top right side)

The bottom row includes the estimates shown in Fig. 2. The treatments of interest for the CINeMA assessment are highlighted with orange color, i.e., agomelatine, benzodiazepine, hydroxyzine, mianserin, pregabalin, quetiapine, and placebo

*HAM-A* Hamilton Anxiety Scale

There was no statistically significant difference in symptoms of anxiety measured on the HAM-A compared with placebo for agomelatine, hydroxyzine and mianserin. The certainty of the evidence assessment is available in Supplementary Table S3. There was no evidence of inconsistency (Supplementary Fig. S6) nor indications of violation of the transitivity assumption (Supplementary Fig. S7). Conducting the network meta-analysis with mean differences instead of SMDs resulted in similar results (Supplementary Fig. S5 and Supplementary Fig. S4B). Likewise, the estimated treatment effects and ranking of the drugs according to SUCRAs using Bayesian network meta-analysis supported our findings (Supplementary Table S4).

The GRADE assessment are presented in the SoF Tables (Supplementary Table S5).

## Discussion

To our knowledge, this study is the first systematic review with network meta-analysis of minor tranquilizers for the short-term treatment of newly onset symptoms of anxiety and distress in adult outpatients. Based on evidence of low to very low certainty, we found that benzodiazepines, pregabalin and quetiapine appear superior to placebo in reducing symptoms of anxiety and that the effect is above that of our predefined threshold for MCID. There were no difference in efficacy between the drugs when evaluated against each other. Data on the critical outcomes of function of daily living and serious adverse events were sparse. Likewise, data on adverse events were generally not systematically reported, and we could not detect clear differences between these three pharmacological treatment options in terms of tolerability. The network meta-analyses confirmed that there was no difference in efficacy between benzodiazepines, pregabalin and quetiapine when evaluated against each other. We did not find evidence of an effect of treatment with either hydroxyzine or agomelatine on symptoms of anxiety. Using the GRADE system as guide, we formulated a weak recommendation for short-term use of one of the three pharmacological options of benzodiazepines with short half-lives, low-dose quetiapine (≤ 150 mg daily) or pregabalin for the short-term treatment of symptoms of anxiety and/or distress in adults.

The majority of included trials included patients with pre-existing anxiety disorders, with only a minority of trials investigating the interventions in patients with adjustment disorders. A further consideration was that in none of the trials were there any indication of whether non-pharmacological treatment options had been tried or considered before the initiation of pharmacological treatment. Also, only few trials reported the duration of symptoms of anxiety or distress and in those instances the duration exceeded 1 year. Thus, although the majority of trials included outpatients, those factors have the consequence that the evidence-base could be considered to be of indirect nature, which led to our downgrading of the certainty of the evidence.

Our review was conducted using rigorous and transparent methods in accordance with the Cochrane Handbook, the PRISMA recommendations and the GRADE-method. We pre-specified the inclusion criteria and critical and important outcomes, judged as critical or important to patients and made those publicly available prior to commencement of the review. Following the GRADE-method we employed a rigorous and transparent method for formulating the research question (PICOT) and assessing the certainty in the evidence by assessing risk of bias, inconsistency, indirectness, imprecision and publication bias for each outcome. Our literature search was comprehensive and included multiple databases, with two independent review authors conducting trial selection, data extraction and risk of bias assessments. We documented the search process transparently. It may be considered a limitation that we restricted inclusion of trials to reports in English, Danish, Swedish and Norwegian, although these constitute the vast majority of relevant trials: of 90 articles identified in full-text 15 studies were excluded for this reason We believe it is unlikely that inclusion of trials reported in other languages would change the results substantially. Furthermore, the search for primary trials was based on the last search date of the included systematic reviews, which may have resulted in not identifying all older relevant primary trials. We did, however, perform hand searches for primary trials in both systematic reviews and primary trials that likely have limited this risk. Our review benefited from the inclusion of a network meta-analysis, which allowed for head-to-head comparison of the effect of available drug options on symptoms of anxiety.

We identified only a few trials in patients with a diagnosis of adjustment disorder, which may be considered a diagnostic category closely resembling our population of interest. Most of the evidence identified for this review and network meta-analysis was thus of indirect character which calls for future research of pharmacological treatment options of this frequent clinical condition that often presents a treatment challenge in primary care.

The results of this systematic review and network meta-analysis document equal efficacy for the short-term treatment of anxiety and distress for benzodiazepines, pregabalin and quetiapine. Likewise, the medications did not substantially differ with respect to the frequency of the predefined adverse events compared with placebo, although for several outcomes the data was limited. However, clinical experience and data from other relevant areas of indication might point to which aspects of a particular clinical case that may aid the clinician in choice of drug. Since the duration of treatment was per definition limited to a few weeks in our review, the risk of development of benzodiazepine dependence is of limited concern, although even short-term treatment may confer a risk of introducing dependence in susceptible individuals. However, if the clinical situation implies a risk of prolonged treatment beyond the short-term time frame, the risk of developing dependence of benzodiazepines increases and would weigh against the choice of benzodiazepines for treatment of newly onset symptoms of anxiety and distress. Likewise, the risk of metabolic effects with prolonged treatment with quetiapine would weigh against the choice of quetiapine in clinical situations with a perceived risk of prolonging treatment beyond 3–4 weeks. Quetiapine is associated with metabolic adverse effects in doses used for recommended indications, e.g., in patients with schizophrenia where 6–8 weeks of treatment at 600 mg/day leads to a difference in weight gain of up to 1.48 kg [79]. In low dose [24] and for short time, the picture is less clear. Low-dose quetiapine (tablet strength 25–50 mg) has been found to be associated with an increased risk of adverse cardiovascular events and cardiovascular death compared with Z-drug hypnotics in a recent Danish register-based cohort study [23], but the finding was based on a median duration of treatment of 2.6 years, i.e., considerably longer than for the population relevant to this review, and may partly be attributable to confounding by indication [80]. A parallel study reported that use of low-dose quetiapine was not associated with excess risk of type-2 diabetes as compared with SSRIs [24]. With regard to pregabalin, the need to titrate the medication to relevant doses might weigh against the choice of pregabalin in clinical situations with need of immediate onset-of-action of the prescribed medication. Additionally, patient preferences regarding expected onset-of-action and risk of adverse effects should play an important role in the final choice of medication for this patient population.

## Conclusion

Evidence of low certainty indicates that short-term treatment with benzodiazepines, pregabalin and quetiapine is superior to placebo in reducing symptoms of anxiety and distress in adults, with no considerable difference between the treatments. Notwithstanding the limitations of the evidence-base, we consider the presented results to be of high clinical relevance and significant utility since the patient group comprises a substantial proportion of patients seeking help in general practice and in various acute outpatient settings. Patient preferences and other individual clinical characteristics are important additional factors to guide the clinicians in the choice of drug.

### Supplementary Information

Below is the link to the electronic supplementary material.

Supplementary file1 (DOCX 2672 KB)

## Data Availability

Data extracted from included studies and details regarding risk of bias assessment are available as part of forest plots availble in the Supplementary Information. Detailed characteristics of the included studies are also available in the Supplementary Information.

## References

[1] Albus M, Lecrubier Y, Maier W, Buller R, Rosenberg R, Hippius H (1990). Drug treatment of panic disorder: early response to treatment as a predictor of final outcome. Acta Psychiatr Scand.

[2] Amore M, Magnani K, Cerisoli M, Casagrande C, Ferrari G (1999). Panic disorder. A long-term treatment study: fluoxetine vs imipramine. Hum Psychopharmacol Clin Exp.

[3] Ansseau M, Bataille M, Briole G, de Nayer A, Fauchere PA, Ferrero F, Van Moffaert M (1996). Controlled comparison of tianeptine, alprazolam and mianserin in the treatment of adjustment disorders with anxiety and depression. Hum Psychopharmacol Clin Exp.

[4] Bakish D, Saxena BM, Bowen R, D’Souza J (1993). Reversible monoamine oxidase-a inhibitors in panic disorder. Clin Neuropharmacol.

[5] Bighelli I, Trespidi C, Castellazzi M, Cipriani A, Furukawa TA, Girlanda F, Guaiana G, Koesters M, Barbui C (2016). Antidepressants and benzodiazepines for panic disorder in adults. The Cochrane Database Syst Rev.

[6] Baandrup L, Kruse M (2016). Incident users of antipsychotics: who are they and how do they fare?. Soc Psychiatry Psychiatr Epidemiol.

[7] Cncps (1992). Drug treatment of panic disorder. Comparative efficacy of alprazolam, imipramine, and placebo. Cross-national collaborative panic study, second phase investigators. Brit J Psychiatry.

[8] Danish Health Authority (2022) National clinical recommendation on the use of mild tranquilizers

[9] Danish Health Authority (2021) National clinical recommendation on the use of mild tranquilizers - focused clinical question

[10] Danish Health Data Authority (2021) Skift i behandlingen af søvnløshed og angsttilstande over de seneste 10 år

[11] Danish Health Data Authority (2018) Stadig færre langtidsbrugere af sovemedicin og angstdæmpende medicin. In:

[12] Danish Health Data Authority (2021) Tredobling i forbrug af det antipsykotiske middel quetiapin i lavdosis gennem de sidste 10 år

[13] Danish Ministry of Health (2008) Vejledning om ordination af afhængighedsskabende lægemidler

[14] De Leo D (1989). Treatment of adjustment disorders: a comparative evaluation. Psychol Rep.

[15] De Wit S, Cremers L, Hirsch D, Zulian C, Clumeck N, Kormoss N (1999). Efficacy and safety of trazodone versus clorazepate in the treatment of hiv-positive subjects with adjustment disorders: a pilot study. J Int Med Res.

[16] Den Boer JA, Westenberg HG (1988). Effect of a serotonin and noradrenaline uptake inhibitor in panic disorder; a double-blind comparative study with fluvoxamine and maprotiline. Int Clin Psychopharmacol.

[17] Emea EMA (2005) Scientific discussion from emea. Product name: Lyrica product no: Mea/h/c/000546/ii/0004. In: EMEA

[18] Eriksen SI, Bjerrum L (2015). Reducing prescriptions of long-acting benzodiazepine drugs in denmark: a descriptive analysis of nationwide prescriptions during a 10-year period. Basic Clin Pharmacol Toxicol.

[19] Feltner DE, Crockatt JG, Dubovsky SJ, Cohn CK, Shrivastava RK, Targum SD, Liu-Dumaw M, Carter CM, Pande AC (2003). A randomized, double-blind, placebo-controlled, fixed-dose, multicenter study of pregabalin in patients with generalized anxiety disorder. J Clin Psychopharmacol.

[20] Guyatt GH, Oxman AD, Kunz R, Atkins D, Brozek J, Vist G, Alderson P, Glasziou P, Falck-Ytter Y, Schunemann HJ (2011). Grade guidelines: 2. Framing the question and deciding on important outcomes. J Clin Epidemiol.

[21] Higgins JP, Altman DG, Gotzsche PC, Juni P, Moher D, Oxman AD, Savovic J, Schulz KF, Weeks L, Sterne JA, Cochrane Bias Methods G, Cochrane Statistical Methods G (2011). The cochrane collaboration’s tool for assessing risk of bias in randomised trials. BMJ.

[22] Higgins JPT, Thomas J, Chandler J, Cumpston M, Li T, Page MJ, Welch VA (2019). Cochrane handbook for systematic reviews of interventions.

[23] Hojlund M, Andersen K, Ernst MT, Correll CU, Hallas J (2022). Use of low-dose quetiapine increases the risk of major adverse cardiovascular events: results from a nationwide active comparator-controlled cohort study. World Psychiatry.

[24] Hojlund M, Lund LC, Andersen K, Correll CU, Hallas J (2021). Association of low-dose quetiapine and diabetes. JAMA Netw Open.

[25] Hojlund M, Rasmussen L, Olesen M, Munk-Olsen T, Pottegard A (2022). Who prescribes quetiapine in denmark?. Br J Clin Pharmacol.

[26] Hutton B, Salanti G, Caldwell DM, Chaimani A, Schmid CH, Cameron C, Ioannidis JP, Straus S, Thorlund K, Jansen JP, Mulrow C, Catala-Lopez F, Gotzsche PC, Dickersin K, Boutron I, Altman DG, Moher D (2015). The prisma extension statement for reporting of systematic reviews incorporating network meta-analyses of health care interventions: checklist and explanations. Ann Intern Med.

[27] Højlund M, Gudmundsson LS, Andersen JH, Saastamoinen LK, Zoega H, Skurtveit SO, Wastesson JW, Hallas J, Pottegård A (2022). Use of benzodiazepines and benzodiazepine-related drugs in the nordic countries between 2000 and 2020. Basic Clin Pharmacol Toxicol.

[28] Islam MM, Conigrave KM, Day CA, Nguyen Y, Haber PS (2014). Twenty-year trends in benzodiazepine dispensing in the australian population. Intern Med J.

[29] Jorgensen MB, Videbech P, Osler M (2017). benzodiazepines still play a role in modern psychiatric therapy. Ugeskr Laeger.

[30] Khan A, Atkinson S, Mezhebovsky I, She F, Leathers T, Pathak S (2011). Extended release quetiapine fumarate (quetiapine xr) as adjunct therapy in patients with generalized anxiety disorder and a history of inadequate treatment response: a randomized, double-blind study. Psychopharmacol Bull.

[31] Kruger MB, Dahl AA (1999). The efficacy and safety of moclobemide compared to clomipramine in the treatment of panic disorder. Eur Arch Psychiatry Clin Neurosci.

[32] Kurko T, Saastamoinen LK, Tuulio-Henriksson A, Taiminen T, Tiihonen J, Airaksinen M, Hietala J (2018). Trends in the long-term use of benzodiazepine anxiolytics and hypnotics: a national register study for 2006 to 2014. Pharmacoepidemiol Drug Saf.

[33] Kurko TA, Saastamoinen LK, Tahkapaa S, Tuulio-Henriksson A, Taiminen T, Tiihonen J, Airaksinen MS, Hietala J (2015). Long-term use of benzodiazepines: definitions, prevalence and usage patterns - a systematic review of register-based studies. Eur Psychiatry.

[34] Lepola U, Heikkinen H, Rimon RRP (1990). Clinical evaluation of alprazolam in patients with panic disorder; a double-blind comparison with imipramine. Hum Psychopharmacol.

[35] Li R, Wu R, Chen J, Kemp DE, Ren M, Conroy C, Chan P, Serrano MB, Ganocy SJ, Calabrese JR, Gao K (2016). A randomized, placebo-controlled pilot study of quetiapine-xr monotherapy or adjunctive therapy to antidepressant in acute major depressive disorder with current generalized anxiety disorder. Psychopharmacol Bull.

[36] Liebowitz MR, Schneier F, Campeas R, Hollander E, Hatterer J, Fyer A, Gorman J, Papp L, Davies S, Gully R (1992). Phenelzine vs atenolol in social phobia. A placebo-controlled comparison. Arch Gen Psychiatry.

[37] Lin L, Zhang J, Hodges JS, Chu H (2017). Performing arm-based network meta-analysis in r with the pcnetmeta package. J Stat Softw.

[38] Llorca PM, Spadone C, Sol O, Danniau A, Bougerol T, Corruble E, Faruch M, Macher JP, Sermet E, Servant D (2002). Efficacy and safety of hydroxyzine in the treatment of generalized anxiety disorder: a 3-month double-blind study. J Clin Psychiatry.

[39] MAGIC Evidence Ecosystem Fundation (2022) Magicapp

[40] Merideth C, Cutler AJ, She F, Eriksson H (2012). Efficacy and tolerability of extended release quetiapine fumarate monotherapy in the acute treatment of generalized anxiety disorder: a randomized, placebo controlled and active-controlled study. Int Clin Psychopharmacol.

[41] Michelson D, Hargreaves R, Alexander R, Ceesay P, Hietala J, Lines C, Reines S (2013). Lack of efficacy of l-759274, a novel neurokinin 1 (substance p) receptor antagonist, for the treatment of generalized anxiety disorder. Int J Neuropsychopharmacol.

[42] Moller HJ, Volz HP, Reimann IW, Stoll KD (2001). Opipramol for the treatment of generalized anxiety disorder: a placebo-controlled trial including an alprazolam-treated group. J Clin Psychopharmacol.

[43] Nguyen N, Fakra E, Pradel V, Jouve E, Alquier C, Le Guern ME, Micallef J, Blin O (2006). Efficacy of etifoxine compared to lorazepam monotherapy in the treatment of patients with adjustment disorders with anxiety: a double-blind controlled study in general practice. Hum Psychopharmacol.

[44] Nikolakopoulou A, Higgins JPT, Papakonstantinou T, Chaimani A, Del Giovane C, Egger M, Salanti G (2020). Cinema: an approach for assessing confidence in the results of a network meta-analysis. PLoS Med.

[45] Noyes R, Burrows GD, Reich JH, Judd FK, Garvey MJ, Norman TR, Cook BL, Marriott P (1996). Diazepam versus alprazolam for the treatment of panic disorder. J Clin Psychiatry.

[46] O’Donnell ML, Metcalf O, Watson L, Phelps A, Varker T (2018). A systematic review of psychological and pharmacological treatments for adjustment disorder in adults. J Trauma Stress.

[47] Olfson M, King M, Schoenbaum M (2015). Benzodiazepine use in the united states. JAMA Psychiat.

[48] Page MJ, McKenzie JE, Bossuyt PM, Boutron I, Hoffmann TC, Mulrow CD, Shamseer L, Tetzlaff JM, Akl EA, Brennan SE, Chou R, Glanville J, Grimshaw JM, Hrobjartsson A, Lalu MM, Li T, Loder EW, Mayo-Wilson E, McDonald S, McGuinness LA, Stewart LA, Thomas J, Tricco AC, Welch VA, Whiting P, Moher D (2021). The prisma 2020 statement: an updated guideline for reporting systematic reviews. PLoS Med.

[49] Pande AC, Crockatt JG, Feltner DE, Janney CA, Smith WT, Weisler R, Londborg PD, Bielski RJ, Zimbroff DL, Davidson JR, Liu-Dumaw M (2003). Pregabalin in generalized anxiety disorder: a placebo-controlled trial. Am J Psychiatry.

[50] Philip NS, Mello K, Carpenter LL, Tyrka AR, Price LH (2008). Patterns of quetiapine use in psychiatric inpatients: an examination of off-label use. Ann Clin Psychiatry.

[51] Razavi D, Kormoss N, Collard A, Farvacques C, Delvaux N (1999). Comparative study of the efficacy and safety of trazodone versus clorazepate in the treatment of adjustment disorders in cancer patients: a pilot study. J Int Med Res.

[52] Rickels K, Pollack MH, Feltner DE, Lydiard RB, Zimbroff DL, Bielski RJ, Tobias K, Brock JD, Zornberg GL, Pande AC (2005). Pregabalin for treatment of generalized anxiety disorder: a 4-week, multicenter, double-blind, placebo-controlled trial of pregabalin and alprazolam. Arch Gen Psychiatry.

[53] Rocca P, Fonzo V, Scotta M, Zanalda E, Ravizza L (1997). Paroxetine efficacy in the treatment of generalized anxiety disorder. Acta Psychiatr Scand.

[54] Rücker G, Krahn U, König J, Efthimiou O, Davies A, Papakonstantinou T, Schwarzer G (2022) Netmeta: network meta-analysis using frequentist methods. In: R package version 25-0

[55] Rücker G, Schwarzer G (2015). Ranking treatments in frequentist network meta-analysis works without resampling methods. BMC Med Res Methodol.

[56] Schweizer E, Rickels K, Weiss S, Zavodnick S (1993). Maintenance drug treatment of panic disorder. I. Results of a prospective, placebo-controlled comparison of alprazolam and imipramine. Arch Gen Psychiatry.

[57] Schünemann H, Brożek J, Guyatt G, Oxman A, editors (2013) Grade handbook for grading quality of evidence and strength of recommendations. Updated october 2013., Available from guidelinedevelopment.org/handbook

[58] Shea BJ, Hamel C, Wells GA, Bouter LM, Kristjansson E, Grimshaw J, Henry DA, Boers M (2009). Amstar is a reliable and valid measurement tool to assess the methodological quality of systematic reviews. J Clin Epidemiol.

[59] Sidorchuk A, Isomura K, Molero Y, Hellner C, Lichtenstein P, Chang Z, Franck J, Fernández de la Cruz L, Mataix-Cols D (2018). Benzodiazepine prescribing for children, adolescents, and young adults from 2006 through 2013: a total population register-linkage study. PLoS Med.

[60] Slee A, Nazareth I, Bondaronek P, Liu Y, Cheng Z, Freemantle N (2019). Pharmacological treatments for generalised anxiety disorder: a systematic review and network meta-analysis. Lancet.

[61] Song MF, Hu LL, Liu WJ, Liu Y, Tao XY, Wang TT, Wang SD, Zhang L, Zhang YH (2017). Modified suanzaorentang had the treatment effect for generalized anxiety disorder for the first 4 weeks of paroxetine medication: a pragmatic randomized controlled study. Evid Based Complement Alternat Med.

[62] Soyka M (2017). Treatment of benzodiazepine dependence. N Engl J Med.

[63] Stein DJ (2015). Etifoxine versus alprazolam for the treatment of adjustment disorder with anxiety: a randomized controlled trial. Adv Ther.

[64] Stein DJ, Ahokas A, Jarema M, Avedisova AS, Vavrusova L, Chaban O, Gruget C, Olivier V, Picarel-Blanchot F, de Bodinat C (2017). Efficacy and safety of agomelatine (10 or 25 mg/day) in non-depressed out-patients with generalized anxiety disorder: a 12-week, double-blind, placebo-controlled study. Eur Neuropsychopharmacol.

[65] Stein DJ, Ahokas AA, de Bodinat C (2008). Efficacy of agomelatine in generalized anxiety disorder: a randomized, double-blind, placebo-controlled study. J Clin Psychopharmacol.

[66] Stein DJ, Baldwin DS, Baldinetti F, Mandel F (2008). Efficacy of pregabalin in depressive symptoms associated with generalized anxiety disorder: a pooled analysis of 6 studies. Eur Neuropsychopharmacol.

[67] Tahkapaa SM, Saastamoinen L, Airaksinen M, Tuulio-Henriksson A, Aalto-Setala T, Kurko T (2018). Decreasing trend in the use and long-term use of benzodiazepines among young adults. J Child Adolesc Psychopharmacol.

[68] Taipale H, Sarkila H, Tanskanen A, Kurko T, Taiminen T, Tiihonen J, Sund R, Tuulio-Henriksson A, Saastamoinen L, Hietala J (2020). Incidence of and characteristics associated with long-term benzodiazepine use in finland. JAMA Netw Open.

[69] Taylor CB, Hayward C, King R, Ehlers A, Margraf J, Maddock R, Clark D, Roth WT, Agras WS (1990). Cardiovascular and symptomatic reduction effects of alprazolam and imipramine in patients with panic disorder: results of a double-blind, placebo-controlled trial. J Clin Psychopharmacol.

[70] Thabane L, Thomas T, Ye C, Paul J (2009). Posing the research question: not so simple. Can J Anaesth.

[71] Torres-Bondia F, de Batlle J, Galván L, Buti M, Barbé F, Piñol-Ripoll G (2020). Trends in the consumption rates of benzodiazepines and benzodiazepine-related drugs in the health region of lleida from 2002 to 2015. BMC Public Health.

[72] van Valkenhoef G, Kuiper J (2021) Gemtc: network meta-analysis using bayesian methods. In: R package version 10-1

[73] van Vliet IM, den Boer JA, Westenberg HG (1992). Psychopharmacological treatment of social phobia: clinical and biochemical effects of brofaromine, a selective mao-a inhibitor. Eur Neuropsychopharmacol.

[74] van Vliet IM, den Boer JA, Westenberg HG, Pian KL (1997). Clinical effects of buspirone in social phobia: a double-blind placebo-controlled study. J Clin Psychiatry.

[75] Versiani M, Nardi AE, Figueira I, Mendlowicz M, Marques C (1997). Double-blind placebo controlled trial with bromazepam in social phobia. J Bras Psiquiatr.

[76] Versiani M, Nardi AE, Mundim FD, Alves AB, Liebowitz MR, Amrein R (1992). Pharmacotherapy of social phobia. A controlled study with moclobemide and phenelzine. Br J Psychiatry.

[77] Williams T, McCaul M, Schwarzer G, Cipriani A, Stein DJ, Ipser J (2020). Pharmacological treatments for social anxiety disorder in adults: a systematic review and network meta-analysis. Acta Neuropsychiatr.

[78] Woods A, Begum M, Gonzalez-Chica D, Bernardo C, Hoon E, Stocks N (2022). Long-term benzodiazepines and z-drug prescribing in australian general practice between 2011 and 2018: a national study. Pharmacol Res Perspect.

[79] Wu H, Siafis S, Hamza T, Schneider-Thoma J, Davis JM, Salanti G, Leucht S (2022). Antipsychotic-induced weight gain: dose-response meta-analysis of randomized controlled trials. Schizophr Bull.

[80] Østergaard SD, Rohde C (2022). The use of low-dose quetiapine does not necessarily increase the risk of major adverse cardiovascular events. Acta Neuropsychiatr.

