# Supplementary material for: Minor tranquillizers for short-term treatment of newly onset symptoms of anxiety and distress: a systematic review with network meta-analysis of randomized trials
Source: Eur Arch Psychiatry Clin Neurosci. 2023 Aug 25;274(3):475–86. doi: 10.1007/s00406-023-01680-0 (PMC10995039; doi:10.1007/s00406-023-01680-0)
Supplement: Supplementary file 1 — Supplementary file1 (DOCX 2672 KB) [file 406_2023_1680_MOESM1_ESM.docx]

Minor tranquillizers for short-term treatment of newly-onset symptoms of anxiety and distress: A systematic review with network meta-analysis of randomized trials

Supplement

***Table of contents***

**Search strategies**

**Deviations from the protocol**

**Supplementary Figure S1.** Prisma flow chart.

**Supplementary Table S1.** Characteristics of included studies.

**Supplementary Table S2.** List of excluded articles after full text-review.

**Supplementary Table S3.** AMSTAR assessment of included systematic reviews.

**Supplementary Figure S2.** Risk of bias assessment.

## Supplementary Table S3. CINeMA assessment.

**Supplementary Figure S3**. Forest plot for the effect of each treatment against placebo on HAM-A (SMDs with 95% CrI) – Bayesian (sensitivity).

**Supplementary Figure S4**. Ranking of treatments.

# Supplementary Figure S5. Forest plot for the effect of each treatment against placebo on HAM-A (MDs with 95%CIs).

# Supplementary Figure S6. Consistency assessed with node splitting.

# Supplementary Figure S7. Transitivity.

# Supplementary Table S4. SUCRA (based on SMD) – Bayesian (sensitivity).

## Supplementary results text

# Supplementary Figure S8. Forest plots of pair-wise meta-analyses.

# Supplementary Table S5. Summary of Findings Tables.

# Search Strategies

## Primary literature search (February 2022)

Medline (110222)

Database(s): **Ovid MEDLINE(R) and Epub Ahead of Print, In-Process, In-Data-Review & Other Non-Indexed Citations, Daily and Versions(R)**1946 to February 10, 2022
Search Strategy:

| **#** | **Searches** | **Results** |
| --- | --- | --- |
| 1 | Anxiety/ or anxiety.mp. | 274139 |
| 2 | axious*.mp. | 1 |
| 3 | Anxiety Disorders/ or anxiety disorder*.mp. | 58593 |
| 4 | Neurotic Disorders/ or neurotic disorder*.mp. | 18419 |
| 5 | Stress, Psychological/ or Stress Disorders, Traumatic, Acute/ or acute stress*.mp. | 135501 |
| 6 | mental stress.mp. | 3634 |
| 7 | Adjustment Disorders/ or adjustment disorder*.mp. | 5615 |
| 8 | Sleep Wake Disorders/ or insomnia*.mp. | 47862 |
| 9 | "Sleep Initiation and Maintenance Disorders"/ or secondary insomnia*.mp. | 15279 |
| 10 | sleeplessness*.mp. | 714 |
| 11 | crisis*.mp. | 74372 |
| 12 | Life Change Events/ or life crisis*.mp. or Stress, Psychological/ | 148138 |
| 13 | or/1-12 | 533007 |
| 14 | (benzodiazepin* or BZD or Abecarnil* or Adinazolam* or Alprazolam* or Arfendazam* or Bentazepam* or Bretazenil* or Bromazepam* or Bromazepamor* or Brotizolam* or Camazepam* or Chlordiazepoxide* or Chlordesmethyldiazepam* or Cinolazepam* or Clobazam* or Clonazepam* or Clorazepate* or Chlorazepate* or Clotiazepam* or Cloxazolam* or Delorazepam* or Demoxepam* or Desmethyldiazepam* or Desoxydemoxepamor* or Devazepide* or Diazepam* or Doxefazepam* or Estazolam* or Fludiazepamor* or Flunitrazepam* or Flurazepam* or dealkylflurazepam* or Flutoprazepam* or Fosazepam* or Gidazepam* or Girisopam* or Halazepamor* or Haloxazolam* or Ketazolam* or Loflazepate* or Loprazolam* or Lorazepam* or Lormetazepam* or Meclonazepam* or Medazepam* or Metaclazepam* or Mexazolam* or Midazolam* or Nerisopam* or Nimetazepam* or Nitrazepam* or Norchlordiazepoxide* or Norclobazamor* or Nordazepam* or Norfludiazepam* or Norflunitrazepam* or Oxazepam* or Oxazolam* or Phenazepamor* or Pinazepam* or Prazepam* or Premazepam* or Propazepam* or Quazepam* or Ripazepam* or Serazepine* or Sograzepide* or Talampanelor* or Tarazepide* or Temazepam* or Tetrazepam* or Tofisopam* or Triazolam*).mp,sh. | 95896 |
| 15 | Histamine H1 Antagonists/ or antihistamine*.mp. | 23958 |
| 16 | Histamine H1 blocker*.mp. | 30 |
| 17 | Promethazine/ or promethazin*.mp. | 4079 |
| 18 | melatonin*.mp. or Melatonin/ | 29173 |
| 19 | zopiclone*.mp. | 1131 |
| 20 | Zolpidem/ or zolpidem*.mp. | 2682 |
| 21 | z-drug*.mp. | 412 |
| 22 | quetiapin*.mp. | 5600 |
| 23 | Olanzapine/ or olanzapin*.mp. | 9955 |
| 24 | melperone*.mp. | 155 |
| 25 | levomepromazine*.mp. | 536 |
| 26 | Risperidone/ or risperidon*.mp. | 10717 |
| 27 | Mirtazapine/ or mirtazapin*.mp. | 2548 |
| 28 | Mianserin/ or mianserin*.mp. | 3541 |
| 29 | Anti-Anxiety Agents/ or Anti-Anxiety Agent*.mp. | 19253 |
| 30 | (Hypnotic* and Sedative*).mp. [mp=title, abstract, original title, name of substance word, subject heading word, floating sub-heading word, keyword heading word, organism supplementary concept word, protocol supplementary concept word, rare disease supplementary concept word, unique identifier, synonyms] | 33132 |
| 31 | "Hypnotics and Sedatives"/ or sedative*.mp. | 45128 |
| 32 | Antipsychotic Agents/ or antipsychotic*.mp. | 74399 |
| 33 | Antipsychotic drug*.mp. | 10319 |
| 34 | sedating antihistamine*.mp. | 273 |
| 35 | ((anti-anxiety or antianxiety or anti anxiety or Anxiolytic*) adj3 (drug* or agent* or pill*1 or medication* or medicine or effect*)).ti,ab,kw,kf. | 9690 |
| 36 | (((random* or cluster-random* or quasi-random* or control?ed or crossover or cross-over or blind* or mask*) adj4 (trial*1 or study or studies or analy*)) or rct).ti,ab,kf. | 796258 |
| 37 | placebo*.ti,ab,kf. | 233809 |
| 38 | ((single-blind* or double-blind* or triple-blind*) adj2 (method or studies)).ti,ab,kf. | 4331 |
| 39 | ((single or double or triple) adj2 (blind*3 or mask*3) adj2 (method or studies)).ti,ab,kf. | 4413 |
| 40 | or/14-34 | 258669 |
| 41 | 36 or 37 or 38 or 39 | 878898 |
| 42 | 13 and 40 and 41 | 6280 |
| 43 | limit 42 to dt=20150901-20220211 | 1140 |
| 44 | limit 43 to (danish or english or norwegian or swedish) | 1101 |

Embase (110222)

Database(s): **Embase**1996 to 2022 Week 05
Search Strategy:

| **#** | **Searches** | **Results** |
| --- | --- | --- |
| 1 | Anxiety/ or anxiety.mp. | 384985 |
| 2 | axious*.mp. | 2 |
| 3 | Anxiety Disorders/ or anxiety disorder*.mp. | 106344 |
| 4 | Neurotic Disorders/ or neurotic disorder*.mp. | 8830 |
| 5 | Stress, Psychological/ or Stress Disorders, Traumatic, Acute/ or acute stress*.mp. | 74826 |
| 6 | mental stress.mp. | 76536 |
| 7 | Adjustment Disorders/ or adjustment disorder*.mp. | 4450 |
| 8 | Sleep Wake Disorders/ or insomnia*.mp. | 107753 |
| 9 | "Sleep Initiation and Maintenance Disorders"/ or secondary insomnia*.mp. | 25976 |
| 10 | sleeplessness*.mp. | 870 |
| 11 | crisis*.mp. | 76620 |
| 12 | Life Change Events/ or life crisis*.mp. or Stress, Psychological/ | 85028 |
| 13 | or/1-12 | 631412 |
| 14 | (benzodiazepin* or BZD or Abecarnil* or Adinazolam* or Alprazolam* or Arfendazam* or Bentazepam* or Bretazenil* or Bromazepam* or Bromazepamor* or Brotizolam* or Camazepam* or Chlordiazepoxide* or Chlordesmethyldiazepam* or Cinolazepam* or Clobazam* or Clonazepam* or Clorazepate* or Chlorazepate* or Clotiazepam* or Cloxazolam* or Delorazepam* or Demoxepam* or Desmethyldiazepam* or Desoxydemoxepamor* or Devazepide* or Diazepam* or Doxefazepam* or Estazolam* or Fludiazepamor* or Flunitrazepam* or Flurazepam* or dealkylflurazepam* or Flutoprazepam* or Fosazepam* or Gidazepam* or Girisopam* or Halazepamor* or Haloxazolam* or Ketazolam* or Loflazepate* or Loprazolam* or Lorazepam* or Lormetazepam* or Meclonazepam* or Medazepam* or Metaclazepam* or Mexazolam* or Midazolam* or Nerisopam* or Nimetazepam* or Nitrazepam* or Norchlordiazepoxide* or Norclobazamor* or Nordazepam* or Norfludiazepam* or Norflunitrazepam* or Oxazepam* or Oxazolam* or Phenazepamor* or Pinazepam* or Prazepam* or Premazepam* or Propazepam* or Quazepam* or Ripazepam* or Serazepine* or Sograzepide* or Talampanelor* or Tarazepide* or Temazepam* or Tetrazepam* or Tofisopam* or Triazolam*).mp,sh. | 189527 |
| 15 | Histamine H1 Antagonists/ or antihistamine*.mp. | 19418 |
| 16 | Histamine H1 blocker*.mp. | 25 |
| 17 | Promethazine/ or promethazin*.mp. | 7987 |
| 18 | melatonin*.mp. or Melatonin/ | 37261 |
| 19 | zopiclone*.mp. | 3842 |
| 20 | Zolpidem/ or zolpidem*.mp. | 9198 |
| 21 | z-drug*.mp. | 626 |
| 22 | quetiapin*.mp. | 26199 |
| 23 | Olanzapine/ or olanzapin*.mp. | 37524 |
| 24 | melperone*.mp. | 634 |
| 25 | levomepromazine*.mp. | 3048 |
| 26 | Risperidone/ or risperidon*.mp. | 39335 |
| 27 | Mirtazapine/ or mirtazapin*.mp. | 13980 |
| 28 | Mianserin/ or mianserin*.mp. | 4207 |
| 29 | Anti-Anxiety Agents/ or Anti-Anxiety Agent*.mp. | 18226 |
| 30 | (Hypnotic* and Sedative*).mp. [mp=title, abstract, heading word, drug trade name, original title, device manufacturer, drug manufacturer, device trade name, keyword heading word, floating subheading word, candidate term word] | 11977 |
| 31 | "Hypnotics and Sedatives"/ or sedative*.mp. | 37224 |
| 32 | Antipsychotic Agents/ or antipsychotic*.mp. | 102057 |
| 33 | Antipsychotic drug*.mp. | 13376 |
| 34 | sedating antihistamine*.mp. | 368 |
| 35 | ((anti-anxiety or antianxiety or anti anxiety or Anxiolytic*) adj3 (drug* or agent* or pill*1 or medication* or medicine or effect*)).ti,ab,kw,kf. | 10590 |
| 36 | (((random* or cluster-random* or quasi-random* or control?ed or crossover or cross-over or blind* or mask*) adj4 (trial*1 or study or studies or analy*)) or rct).ti,ab,kf. | 1014222 |
| 37 | placebo*.ti,ab,kf. | 290742 |
| 38 | ((single-blind* or double-blind* or triple-blind*) adj2 (method or studies)).ti,ab,kf. | 5265 |
| 39 | ((single or double or triple) adj2 (blind*3 or mask*3) adj2 (method or studies)).ti,ab,kf. | 5375 |
| 40 | or/14-34 | 391477 |
| 41 | 36 or 37 or 38 or 39 | 1111761 |
| 42 | 13 and 40 and 41 | 8512 |
| 43 | limit 42 to yr="2015 - 2022" | 2781 |
| 44 | limit 43 to (danish or english or norwegian or swedish) | 2738 |

PsycInfo (110222)

Database(s): **APA PsycInfo**1806 to January Week 5 2022
Search Strategy:

| **#** | **Searches** | **Results** |
| --- | --- | --- |
| 1 | Anxiety/ or anxiety.mp. | 263251 |
| 2 | axious*.mp. | 1 |
| 3 | Anxiety Disorders/ or anxiety disorder*.mp. | 57231 |
| 4 | Neurotic Disorders/ or neurotic disorder*.mp. | 7357 |
| 5 | Stress, Psychological/ or Stress Disorders, Traumatic, Acute/ or acute stress*.mp. | 5718 |
| 6 | mental stress.mp. | 1225 |
| 7 | Adjustment Disorders/ or adjustment disorder*.mp. | 4371 |
| 8 | Sleep Wake Disorders/ or insomnia*.mp. | 22506 |
| 9 | "Sleep Initiation and Maintenance Disorders"/ or secondary insomnia*.mp. | 59 |
| 10 | sleeplessness*.mp. | 451 |
| 11 | crisis*.mp. | 41768 |
| 12 | Life Change Events/ or life crisis*.mp. or Stress, Psychological/ | 417 |
| 13 | or/1-12 | 333349 |
| 14 | Histamine H1 Antagonists/ or antihistamine*.mp. | 627 |
| 15 | Histamine H1 blocker*.mp. | 2 |
| 16 | Promethazine/ or promethazin*.mp. | 259 |
| 17 | melatonin*.mp. or Melatonin/ | 5410 |
| 18 | zopiclone*.mp. | 303 |
| 19 | Zolpidem/ or zolpidem*.mp. | 967 |
| 20 | z-drug*.mp. | 120 |
| 21 | quetiapin*.mp. | 4015 |
| 22 | Olanzapine/ or olanzapin*.mp. | 6686 |
| 23 | melperone*.mp. | 66 |
| 24 | levomepromazine*.mp. | 154 |
| 25 | Risperidone/ or risperidon*.mp. | 7799 |
| 26 | Mirtazapine/ or mirtazapin*.mp. | 1377 |
| 27 | Mianserin/ or mianserin*.mp. | 1325 |
| 28 | Anti-Anxiety Agents/ or Anti-Anxiety Agent*.mp. | 5241 |
| 29 | (Hypnotic* and Sedative*).mp. | 4633 |
| 30 | "Hypnotics and Sedatives"/ or sedative*.mp. | 8791 |
| 31 | Antipsychotic Agents/ or antipsychotic*.mp. | 42968 |
| 32 | Antipsychotic drug*.mp. | 7605 |
| 33 | sedating antihistamine*.mp. | 16 |
| 34 | ((anti-anxiety or antianxiety or anti anxiety or Anxiolytic*) adj3 (drug* or agent* or pill*1 or medication* or medicine or effect*)).ti,ab,id. | 5257 |
| 35 | (benzodiazepin* or BZD or Abecarnil* or Adinazolam* or Alprazolam* or Arfendazam* or Bentazepam* or Bretazenil* or Bromazepam* or Bromazepamor* or Brotizolam* or Camazepam* or Chlordiazepoxide* or Chlordesmethyldiazepam* or Cinolazepam* or Clobazam* or Clonazepam* or Clorazepate* or Chlorazepate* or Clotiazepam* or Cloxazolam* or Delorazepam* or Demoxepam* or Desmethyldiazepam* or Desoxydemoxepamor* or Devazepide* or Diazepam* or Doxefazepam* or Estazolam* or Fludiazepamor* or Flunitrazepam* or Flurazepam* or dealkylflurazepam* or Flutoprazepam* or Fosazepam* or Gidazepam* or Girisopam* or Halazepamor* or Haloxazolam* or Ketazolam* or Loflazepate* or Loprazolam* or Lorazepam* or Lormetazepam* or Meclonazepam* or Medazepam* or Metaclazepam* or Mexazolam* or Midazolam* or Nerisopam* or Nimetazepam* or Nitrazepam* or Norchlordiazepoxide* or Norclobazamor* or Nordazepam* or Norfludiazepam* or Norflunitrazepam* or Oxazepam* or Oxazolam* or Phenazepamor* or Pinazepam* or Prazepam* or Premazepam* or Propazepam* or Quazepam* or Ripazepam* or Serazepine* or Sograzepide* or Talampanelor* or Tarazepide* or Temazepam* or Tetrazepam* or Tofisopam* or Triazolam*).mp,id. | 25559 |
| 36 | or/14-34 | 68714 |
| 37 | 35 or 36 | 82601 |
| 38 | exp Randomized Controlled Trials/ | 1123 |
| 39 | (((random* or cluster-random* or quasi-random* or control?ed or crossover or cross-over or blind* or mask*) adj4 (trial*1 or study or studies or analy*)) or rct).ti,ab,id. | 118068 |
| 40 | placebo*.ti,ab,id. | 42642 |
| 41 | ((single-blind* or double-blind* or triple-blind*) adj2 (method* or studies)).ti,ab,id. | 1361 |
| 42 | ((single or double or triple) adj1 (blind* or mask*) adj2 (method* or studies)).ti,ab,id. | 1364 |
| 43 | 38 or 39 or 40 or 41 or 42 | 138785 |
| 44 | 13 and 37 and 43 | 3378 |
| 45 | limit 44 to yr="2015 - 2022" | 516 |
| 46 | limit 45 to (danish or english or norwegian or swedish) | 504 |

## Updated primary literature search (September 2022)

Medline (080922)

Database(s): **Ovid MEDLINE(R) and Epub Ahead of Print, In-Process, In-Data-Review & Other Non-Indexed Citations, Daily and Versions**1946 to September 07, 2022
Search Strategy:

| **#** | **Searches** | **Results** |
| --- | --- | --- |
| 1 | Anxiety/ or anxiety.mp. | 288599 |
| 2 | axious*.mp. | 1 |
| 3 | Anxiety Disorders/ or anxiety disorder*.mp. | 61319 |
| 4 | Neurotic Disorders/ or neurotic disorder*.mp. | 18441 |
| 5 | Stress, Psychological/ or Stress Disorders, Traumatic, Acute/ or acute stress*.mp. | 137730 |
| 6 | mental stress.mp. | 3783 |
| 7 | Adjustment Disorders/ or adjustment disorder*.mp. | 5683 |
| 8 | Sleep Wake Disorders/ or insomnia*.mp. | 50231 |
| 9 | "Sleep Initiation and Maintenance Disorders"/ or secondary insomnia*.mp. | 16223 |
| 10 | sleeplessness*.mp. | 750 |
| 11 | crisis*.mp. | 79464 |
| 12 | Life Change Events/ or life crisis*.mp. or Stress, Psychological/ | 150276 |
| 13 | or/1-12 | 555934 |
| 14 | (benzodiazepin* or BZD or Abecarnil* or Adinazolam* or Alprazolam* or Arfendazam* or Bentazepam* or Bretazenil* or Bromazepam* or Bromazepamor* or Brotizolam* or Camazepam* or Chlordiazepoxide* or Chlordesmethyldiazepam* or Cinolazepam* or Clobazam* or Clonazepam* or Clorazepate* or Chlorazepate* or Clotiazepam* or Cloxazolam* or Delorazepam* or Demoxepam* or Desmethyldiazepam* or Desoxydemoxepamor* or Devazepide* or Diazepam* or Doxefazepam* or Estazolam* or Fludiazepamor* or Flunitrazepam* or Flurazepam* or dealkylflurazepam* or Flutoprazepam* or Fosazepam* or Gidazepam* or Girisopam* or Halazepamor* or Haloxazolam* or Ketazolam* or Loflazepate* or Loprazolam* or Lorazepam* or Lormetazepam* or Meclonazepam* or Medazepam* or Metaclazepam* or Mexazolam* or Midazolam* or Nerisopam* or Nimetazepam* or Nitrazepam* or Norchlordiazepoxide* or Norclobazamor* or Nordazepam* or Norfludiazepam* or Norflunitrazepam* or Oxazepam* or Oxazolam* or Phenazepamor* or Pinazepam* or Prazepam* or Premazepam* or Propazepam* or Quazepam* or Ripazepam* or Serazepine* or Sograzepide* or Talampanelor* or Tarazepide* or Temazepam* or Tetrazepam* or Tofisopam* or Triazolam*).mp,sh. | 97356 |
| 15 | Histamine H1 Antagonists/ or antihistamine*.mp. | 24237 |
| 16 | Histamine H1 blocker*.mp. | 30 |
| 17 | Promethazine/ or promethazin*.mp. | 4105 |
| 18 | melatonin*.mp. or Melatonin/ | 30222 |
| 19 | zopiclone*.mp. | 1145 |
| 20 | Zolpidem/ or zolpidem*.mp. | 2745 |
| 21 | z-drug*.mp. | 455 |
| 22 | quetiapin*.mp. | 5740 |
| 23 | Olanzapine/ or olanzapin*.mp. | 10194 |
| 24 | melperone*.mp. | 155 |
| 25 | levomepromazine*.mp. | 540 |
| 26 | Risperidone/ or risperidon*.mp. | 10935 |
| 27 | Mirtazapine/ or mirtazapin*.mp. | 2634 |
| 28 | Mianserin/ or mianserin*.mp. | 3552 |
| 29 | Anti-Anxiety Agents/ or Anti-Anxiety Agent*.mp. | 19418 |
| 30 | (Hypnotic* and Sedative*).mp. [mp=title, book title, abstract, original title, name of substance word, subject heading word, floating sub-heading word, keyword heading word, organism supplementary concept word, protocol supplementary concept word, rare disease supplementary concept word, unique identifier, synonyms] | 33644 |
| 31 | "Hypnotics and Sedatives"/ or sedative*.mp. | 46016 |
| 32 | Antipsychotic Agents/ or antipsychotic*.mp. | 75989 |
| 33 | Antipsychotic drug*.mp. | 10526 |
| 34 | sedating antihistamine*.mp. | 274 |
| 35 | ((anti-anxiety or antianxiety or anti anxiety or Anxiolytic*) adj3 (drug* or agent* or pill*1 or medication* or medicine or effect*)).ti,ab,kw,kf. | 9922 |
| 36 | (((random* or cluster-random* or quasi-random* or control?ed or crossover or cross-over or blind* or mask*) adj4 (trial*1 or study or studies or analy*)) or rct).ti,ab,kf. | 831826 |
| 37 | placebo*.ti,ab,kf. | 239835 |
| 38 | ((single-blind* or double-blind* or triple-blind*) adj2 (method or studies)).ti,ab,kf. | 4405 |
| 39 | ((single or double or triple) adj2 (blind*3 or mask*3) adj2 (method or studies)).ti,ab,kf. | 4488 |
| 40 | or/14-34 | 263772 |
| 41 | 36 or 37 or 38 or 39 | 916040 |
| 42 | 13 and 40 and 41 | 6379 |
| 43 | limit 42 to dt=20150901-20220211 | 1152 |
| 44 | limit 43 to (danish or english or norwegian or swedish) | 1113 |
| 45 | limit 42 to yr="2022 -Current" | 125 |

Embase (090922)

Database(s): **Embase**1996 to 2022 Week 35
Search Strategy:

| **#** | **Searches** | **Results** |
| --- | --- | --- |
| 1 | Anxiety/ or anxiety.mp. | 408358 |
| 2 | axious*.mp. | 2 |
| 3 | Anxiety Disorders/ or anxiety disorder*.mp. | 113059 |
| 4 | Neurotic Disorders/ or neurotic disorder*.mp. | 9425 |
| 5 | Stress, Psychological/ or Stress Disorders, Traumatic, Acute/ or acute stress*.mp. | 78067 |
| 6 | mental stress.mp. | 79552 |
| 7 | Adjustment Disorders/ or adjustment disorder*.mp. | 4645 |
| 8 | Sleep Wake Disorders/ or insomnia*.mp. | 114573 |
| 9 | "Sleep Initiation and Maintenance Disorders"/ or secondary insomnia*.mp. | 31525 |
| 10 | sleeplessness*.mp. | 925 |
| 11 | crisis*.mp. | 82213 |
| 12 | Life Change Events/ or life crisis*.mp. or Stress, Psychological/ | 88069 |
| 13 | or/1-12 | 667130 |
| 14 | (benzodiazepin* or BZD or Abecarnil* or Adinazolam* or Alprazolam* or Arfendazam* or Bentazepam* or Bretazenil* or Bromazepam* or Bromazepamor* or Brotizolam* or Camazepam* or Chlordiazepoxide* or Chlordesmethyldiazepam* or Cinolazepam* or Clobazam* or Clonazepam* or Clorazepate* or Chlorazepate* or Clotiazepam* or Cloxazolam* or Delorazepam* or Demoxepam* or Desmethyldiazepam* or Desoxydemoxepamor* or Devazepide* or Diazepam* or Doxefazepam* or Estazolam* or Fludiazepamor* or Flunitrazepam* or Flurazepam* or dealkylflurazepam* or Flutoprazepam* or Fosazepam* or Gidazepam* or Girisopam* or Halazepamor* or Haloxazolam* or Ketazolam* or Loflazepate* or Loprazolam* or Lorazepam* or Lormetazepam* or Meclonazepam* or Medazepam* or Metaclazepam* or Mexazolam* or Midazolam* or Nerisopam* or Nimetazepam* or Nitrazepam* or Norchlordiazepoxide* or Norclobazamor* or Nordazepam* or Norfludiazepam* or Norflunitrazepam* or Oxazepam* or Oxazolam* or Phenazepamor* or Pinazepam* or Prazepam* or Premazepam* or Propazepam* or Quazepam* or Ripazepam* or Serazepine* or Sograzepide* or Talampanelor* or Tarazepide* or Temazepam* or Tetrazepam* or Tofisopam* or Triazolam*).mp,sh. | 195110 |
| 15 | Histamine H1 Antagonists/ or antihistamine*.mp. | 19893 |
| 16 | Histamine H1 blocker*.mp. | 25 |
| 17 | Promethazine/ or promethazin*.mp. | 8051 |
| 18 | melatonin*.mp. or Melatonin/ | 38806 |
| 19 | zopiclone*.mp. | 3978 |
| 20 | Zolpidem/ or zolpidem*.mp. | 9532 |
| 21 | z-drug*.mp. | 685 |
| 22 | quetiapin*.mp. | 27103 |
| 23 | Olanzapine/ or olanzapin*.mp. | 38688 |
| 24 | melperone*.mp. | 648 |
| 25 | levomepromazine*.mp. | 3122 |
| 26 | Risperidone/ or risperidon*.mp. | 40404 |
| 27 | Mirtazapine/ or mirtazapin*.mp. | 14576 |
| 28 | Mianserin/ or mianserin*.mp. | 4180 |
| 29 | Anti-Anxiety Agents/ or Anti-Anxiety Agent*.mp. | 18908 |
| 30 | (Hypnotic* and Sedative*).mp. [mp=title, abstract, heading word, drug trade name, original title, device manufacturer, drug manufacturer, device trade name, keyword heading word, floating subheading word, candidate term word] | 12266 |
| 31 | "Hypnotics and Sedatives"/ or sedative*.mp. | 38446 |
| 32 | Antipsychotic Agents/ or antipsychotic*.mp. | 104611 |
| 33 | Antipsychotic drug*.mp. | 13595 |
| 34 | sedating antihistamine*.mp. | 373 |
| 35 | ((anti-anxiety or antianxiety or anti anxiety or Anxiolytic*) adj3 (drug* or agent* or pill*1 or medication* or medicine or effect*)).ti,ab,kw,kf. | 10840 |
| 36 | (((random* or cluster-random* or quasi-random* or control?ed or crossover or cross-over or blind* or mask*) adj4 (trial*1 or study or studies or analy*)) or rct).ti,ab,kf. | 1060671 |
| 37 | placebo*.ti,ab,kf. | 299523 |
| 38 | ((single-blind* or double-blind* or triple-blind*) adj2 (method or studies)).ti,ab,kf. | 5373 |
| 39 | ((single or double or triple) adj2 (blind*3 or mask*3) adj2 (method or studies)).ti,ab,kf. | 5483 |
| 40 | or/14-34 | 403159 |
| 41 | 36 or 37 or 38 or 39 | 1160518 |
| 42 | 13 and 40 and 41 | 8808 |
| 43 | limit 42 to yr="2022 -Current" | 315 |

PsycInfo (090922)

Database(s): **APA PsycInfo**1806 to August Week 5 2022
Search Strategy:

| **#** | **Searches** | **Results** |
| --- | --- | --- |
| 1 | Anxiety/ or anxiety.mp. | 271955 |
| 2 | axious*.mp. | 1 |
| 3 | Anxiety Disorders/ or anxiety disorder*.mp. | 59198 |
| 4 | Neurotic Disorders/ or neurotic disorder*.mp. | 7365 |
| 5 | Stress, Psychological/ or Stress Disorders, Traumatic, Acute/ or acute stress*.mp. | 5902 |
| 6 | mental stress.mp. | 1258 |
| 7 | Adjustment Disorders/ or adjustment disorder*.mp. | 4406 |
| 8 | Sleep Wake Disorders/ or insomnia*.mp. | 23486 |
| 9 | "Sleep Initiation and Maintenance Disorders"/ or secondary insomnia*.mp. | 60 |
| 10 | sleeplessness*.mp. | 462 |
| 11 | crisis*.mp. | 43860 |
| 12 | Life Change Events/ or life crisis*.mp. or Stress, Psychological/ | 424 |
| 13 | or/1-12 | 344736 |
| 14 | Histamine H1 Antagonists/ or antihistamine*.mp. | 634 |
| 15 | Histamine H1 blocker*.mp. | 2 |
| 16 | Promethazine/ or promethazin*.mp. | 260 |
| 17 | melatonin*.mp. or Melatonin/ | 5497 |
| 18 | zopiclone*.mp. | 305 |
| 19 | Zolpidem/ or zolpidem*.mp. | 979 |
| 20 | z-drug*.mp. | 131 |
| 21 | quetiapin*.mp. | 4089 |
| 22 | Olanzapine/ or olanzapin*.mp. | 6773 |
| 23 | melperone*.mp. | 66 |
| 24 | levomepromazine*.mp. | 156 |
| 25 | Risperidone/ or risperidon*.mp. | 7873 |
| 26 | Mirtazapine/ or mirtazapin*.mp. | 1405 |
| 27 | Mianserin/ or mianserin*.mp. | 1328 |
| 28 | Anti-Anxiety Agents/ or Anti-Anxiety Agent*.mp. | 5253 |
| 29 | (Hypnotic* and Sedative*).mp. | 4675 |
| 30 | "Hypnotics and Sedatives"/ or sedative*.mp. | 8932 |
| 31 | Antipsychotic Agents/ or antipsychotic*.mp. | 43566 |
| 32 | Antipsychotic drug*.mp. | 7676 |
| 33 | sedating antihistamine*.mp. | 17 |
| 34 | ((anti-anxiety or antianxiety or anti anxiety or Anxiolytic*) adj3 (drug* or agent* or pill*1 or medication* or medicine or effect*)).ti,ab,id. | 5328 |
| 35 | (benzodiazepin* or BZD or Abecarnil* or Adinazolam* or Alprazolam* or Arfendazam* or Bentazepam* or Bretazenil* or Bromazepam* or Bromazepamor* or Brotizolam* or Camazepam* or Chlordiazepoxide* or Chlordesmethyldiazepam* or Cinolazepam* or Clobazam* or Clonazepam* or Clorazepate* or Chlorazepate* or Clotiazepam* or Cloxazolam* or Delorazepam* or Demoxepam* or Desmethyldiazepam* or Desoxydemoxepamor* or Devazepide* or Diazepam* or Doxefazepam* or Estazolam* or Fludiazepamor* or Flunitrazepam* or Flurazepam* or dealkylflurazepam* or Flutoprazepam* or Fosazepam* or Gidazepam* or Girisopam* or Halazepamor* or Haloxazolam* or Ketazolam* or Loflazepate* or Loprazolam* or Lorazepam* or Lormetazepam* or Meclonazepam* or Medazepam* or Metaclazepam* or Mexazolam* or Midazolam* or Nerisopam* or Nimetazepam* or Nitrazepam* or Norchlordiazepoxide* or Norclobazamor* or Nordazepam* or Norfludiazepam* or Norflunitrazepam* or Oxazepam* or Oxazolam* or Phenazepamor* or Pinazepam* or Prazepam* or Premazepam* or Propazepam* or Quazepam* or Ripazepam* or Serazepine* or Sograzepide* or Talampanelor* or Tarazepide* or Temazepam* or Tetrazepam* or Tofisopam* or Triazolam*).mp,id. | 25857 |
| 36 | or/14-34 | 69688 |
| 37 | 35 or 36 | 83774 |
| 38 | exp Randomized Controlled Trials/ | 1268 |
| 39 | (((random* or cluster-random* or quasi-random* or control?ed or crossover or cross-over or blind* or mask*) adj4 (trial*1 or study or studies or analy*)) or rct).ti,ab,id. | 122387 |
| 40 | placebo*.ti,ab,id. | 43282 |
| 41 | ((single-blind* or double-blind* or triple-blind*) adj2 (method* or studies)).ti,ab,id. | 1387 |
| 42 | ((single or double or triple) adj1 (blind* or mask*) adj2 (method* or studies)).ti,ab,id. | 1390 |
| 43 | 38 or 39 or 40 or 41 or 42 | 143361 |
| 44 | 13 and 37 and 43 | 3414 |
| 45 | limit 44 to yr="2022 -Current" | 31 |

# Deviations from the protocol

We included agomelatine and pregabalin although these drugs were not specified in our pre-defined PICOT. Pregabalin was included because multiple trials included pregabalin, the effect on symptoms of anxiety appeared superior to placebo and because the guideline panel considered pregabalin a clinically relevant treatment option after further consideration. Agomelatine was included as we found no studies on melatonin, which was predefined in our PICOT, and agomelatine has melatonin receptor agonist properties. The network meta-analysis also showed that clomipramine appeared to be superior compared to placebo for the reduction of symptoms of anxiety, but the guideline panel did not include that drug for further consideration as it found that in clinical practice the adverse effect profile of the drug would preclude its use for this condition.

# Supplementary Figure S1A. PRISMA flow chart.


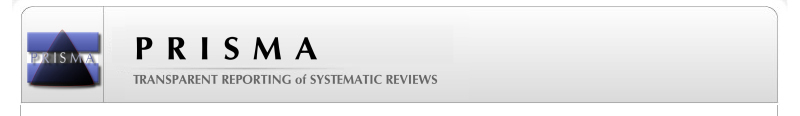


Search for systematic reviews (performed 14^th^ January 2022)

Records identified through database searching
(n =4162)

Screening

Included

Eligibility

Identification

Additional records identified through other sources
(n = 0)

Records after duplicates removed
(n = 2828)

Records screened
(n = 2828)

Records excluded
(n = 2735)

Full-text articles assessed for eligibility
(n = 93)

Full-text articles excluded, with reasons

(n = 89)

Wrong study design (n=18)

Wrong population (n=58)

Wrong intervention (n=4)

Wrong outcome (n=1)

Abstract (n=6)

Full text unavailable (n=4)

Primary trials already identified in the systematic reviews

(n=2)

Systematic review included
(n = 4)

Primary trials included from the identified systematic review (n = 30)

**Supplementary Figure S1B**.
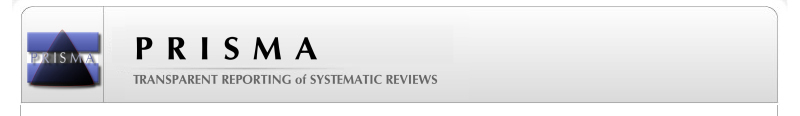
PRISMA 2009 Flow Diagram.

Search for primary studies (performed 11^th^ February 2022 updated 8^th^ September 2022)

Records identified through database searching
(n = 4814)

Screening

Included

Eligibility

Identification

Additional records identified through other sources
(n = 0)

Records after duplicates removed
(n = 3419)

Full-text articles assessed for eligibility
(n = 28)

Records excluded
(n = 3391)

Records screened
(n = 3419)

Full-text articles excluded, with reasons
(n = 24)

Wrong population (n=4)

Wrong intervention (n=10)

Wrong comparison (n=1)

Wrong outcome, no short-term data for critical outcomes (n=7)

Study already included from the systematic reviews (n=2)

Primary studies included
(n = 4)

**Supplementary Table S1.** Characteristics of included studies.

| **Author, year, country** | **Participants n, mean age, (SD), females n (%), diagnosis, duration of symptoms (SD), outpatients (%)** | **Design and funding** | **Interventions** | **Outcomes of interest, timepoint of measurement for extracted data** |
| --- | --- | --- | --- | --- |
| CNCPS^1^, 1990,  International | n = 1168  Overall:  Age in years: 34 (SD not provided)  Females: 62%  Diagnosis: DSM-III panic disorder with limited or extensive phobic avoidance (panic attacks with agoraphobia)  Duration of symptoms: No information  Outpatients (%): Inpatients and outpatients, no further information | RCT, multicenter  12 centres in USA, Spain, Denmark, Germany, England, Italy, Brazil, Mexico, France, Colombia, Austria, Sweden, Canada, Belgium, parallel group, three arms.  Funding: Sponsored by Upjohn Company, Kalamazoo, Michigan | Antidepressant tricyclic (imipramine)  Imipramine 25-250 mg.  The unit dosage was 25 mg of imipramine.  Benzodiazepine (alprazolam)  Alprazolam 1-10 mg.  The unit dosage was 1 mg of alprazolam  Placebo  1-10 placebo capsules  For all interventions: The number of capsules (25 mg of imipramine, 1 mg of alprazolam or placebo) was increased steadily according to a predetermined scedule of 6 capsules at day 19. The number of capsules could be raised or lowered depending on the individual patients clinical state or adverse effect. | Anxiety: HAM-A at 1 week  Serious adverse events: Number of participants, during 8 weeks Suicidal thoughts/attempts: Number of participants, during 8 weeks  Weight changes: Number of participants, during 8 weeks  Drowsiness during daytime: Number of participants, during 8 weeks  Dizziness: Number of participants, during 8 weeks |
| Amore^2^, 1990, Italy | n = 38  Fluoxetine group:  Age in years: 37.0 (SD = 7.1)  Females: 57.89%  Duration of symptoms, years: 5.6 (5.1)  Imipramine group:  Age in years: 37.2 (SD = 8.2)  Females: 36.84%  Duration of symptoms, years: 5.5 (4.2)  Overall:  Diagnosis: DSM-IV Panic Disorder with or without agoraphobia  Outpatients (%): No information | RCT, single center, parallel group, two arms  Funding: Not stated | SSRI (Fluoxetine)  Flexible dosage; range = 10 - 50 mg, mean = 20 mg/day (SD = 10)  Antidepressant tricyclic (Imipramine)  Flexible dosage; range = 25 - 250 mg, mean = 150 mg/day (SD = 25) | Anxiety: HAM-A at 1 week |
| Ansseau^3^, 1996, Belgium, Switzerland and France | n=152  Alprazolam group:  Age in years: 44.2 (11.1)  Duration of symptoms, days: 60.7 (36.6)  Mianserin group:  Age in years: 42.8 (12.6)  Duration of symptoms, days: 66.2 (45.1)  Tianeptine group:  Age in years: 43.6 (10.7)  Duration of symptoms, days: 62.5 (40.2)  Overall:  Females: 70.6 %  Diagnosis: DSM-III-R diagnostic criteria for adjustment disorder with mixed emotional feature (anxiety and depression)  Outpatients (%): 100% | RCT, multicenter 11 centers in Belgium, Switzerland and France, parallel group, three arms.  Funding: Supported by a grant from the ‘Institut de Recherches Internationales Servier" | Benzodiazepine (alprazolam)  Alprazolam (1.5 mg/day)  Antidepressant (mianserin)  Mianserin (60 mg/day)  Antidepressant (tianeptine)  Tianeptine (37.5 mg/day)  For all interventions: All active compounds were then administered in three daily intakes. The daily dose was kept stable during the initial 2-week treatment period and could then be adapted according to efficacy and tolerability between 25 and 50 mg/day for tianeptine, between 40 and 80 mg/day for mianserin, and between 1 and 2 mg/day for alprazolam. | Anxiety: HAM-A at 1 week  Serious adverse events: Number of participants during 6 weeks  Drowsiness during daytime: Number of participants during 6 weeks  Dizziness: Number of participants, during 6 weeks |
| Bakish^4^, 1993, Canada | n = 93  Overall:  Age in years: No information  Females: No information  Diagnosis: DSM-III panic disorder with or without agoraphobia  Duration of symptoms: No information  Outpatients (%): No information | RCT, single center, parallel group, two arms  Funding: Not stated | Antidepressant MAO (brofaromine)  Flexible dosage; range = 50 - 150 mg, M and SD not provided. Medication started at 50 mg daily and increased by 50 mg each week to achive a maximum tolerable dose.  Antidepressant tricyclic (clomipramine)  Flexible dosage, range = 25 - 75 mg, M and SD not provided. Medication started at 25 mg daily and increased by 25 mg each week to achive a maximum tolerable dose. | Anxiety: HAM-A at 1 week |
| De Leo^5^, 1989, Italy | n = 85  Overall:  Age in years: 38.3 (SD not provided)  Females: 60%  Diagnosis: Adjustment disorder with depressed mood or with mixed emotional features (DSM-III)  Duration of symptoms: No information  Outpatients (%): 100% | RCT, single center, parallel group, five arms  Funding: Not stated | Antidepressant (Viloxazine)  Benzodiazepine (Lormetazepam)  Lormetazepam 2 mg/dag  S-adenosylmetioine  100 mg/day intramusculary  Psychotherapy  Psychotherapy psycho analytically oriented  Placebo | No relevant outcomes reported for our interventions of interest |
| Den Boer^6^, 1988, The Netherlands | n = 47  Maprotiline group:  Age in years: 35.0 (7.4)  Females: 83%  Duration of symptoms, years: Minimum 1 year, mean 9.25 (5.8)  Fluvoxamine group:  Age in years: 37.3 (10.6)  Females: 75%  Duration of symptoms:  Overall:  Diagnosis: DSM-III panic disorder without phobic avoidance or panic disorder with severe phobic avoidance behaviour. | RCT, single center, parallel group, two arms  Funding: Not stated | Antidepressant tricyclic (Maprotiline)  Flexible dosage, range = 50 - 150 mg, M and SD not provided. Medication was started with 50 mg daily and gradually increased in 2 weeks to 150 mg.  Antidepressant tricyclic (Fluvoxamine)  Flexible dosage, range = 50 - 150 mg, M and SD not provided. Medication was started with 50 mg daily and gradually increased in 2 weeks to 150 mg. | Anxiety: HAM-A at 1 week |
| De wit^7^, 1999, Belgium | n = 21  Overall:  Age in years, median (range): 36.5 years for females (29 - 44), and 27.5 years for males (18 - 46)  Females: 20%  Diagnosis: HIV and fulfilment of DSM-III-R criteria for the diagnosis of adjustment disorders with anxiety or depressed mood and/or mixed disturbance of emotion and conduct  Duration of symptoms: No information  Outpatients (%):No information | RCT, single center, parallel group, two arms  Funding: Supported by Searle Continental Pharma, Inc., Brussels, Belgium | Antidepressant (Trazodone)  Trazodone 50-150 mg, mean 97.6 mg/day  Benzodiazepine (Clorazepate)  Clorazepate 10-30 mg., mean 15.6 mg/day | No relevant outcomes reported for our interventions of interest |
| EMEA^8^, study 25 2005, unpublished | n= 266  Overall:  Age in years: No information  Females: men and women, no further information  Diagnosis: GAD DSM-IV criteria.  Duration of symptoms: No information  Outpatients (%): 100 % | RCT, parallel group, four arms  Funding: Not stated | Benzodiazepine (Lorazepam)  Lorazepam 6 mg  Pregabalin 150 mg  Pregabalin 150 mg given as three dived doses.  Pregabalin 600 mg  Pregabalin 600 mg, flexible doses, 600 mg given as three dived doses. Pregabalin started at 150 mg/day. Based on individual patient response and tolerability the dose may be increased to 300 mg a day after 1 week, and to 450 mg after 2 weeks og to 600 mg after 3 weeks.  Placebo | Anxiety: HAM-A at 4 weeks  Addiction: Withdrawal symptoms measured with Physician withdrawal checklist (PWC), measured up to two weeks after discontinuation of study drugs (at 6-7 weeks) |
| Feltner^9^, 2003, USA | n = 210  Lorazepam group:  Age in years: 39.2 (11.7)  Females: 58.8 %  Pregabalin 150 mg group:  Age in years: 37.9 (10.9)  Females: 51.4 %  Pregabalin 600 mg group:  Age in years: 36.3 (10.9)  Females: 50 %  Placebo group:  Age in years: 37.8 (10.8)  Females: 50.7 %  Overall:  Diagnosis: GAD DSM-IV criteria.  Duration of symptoms: No information  Outpatients (%): 100 % | RCT, multicenter (4 centers) parallel group, four arms  Funding: Parke-Davis Pharmaceutical research, a division of the Warner-Lambert Company (now Pfiezer) | Benzodiazepine (lorazepam)  Fixed dose 6 mg, 2 mg three times a day.  Pregabalin 150 mg  50 mg three times a day  Pregabalin 600 mg  200 mg three times a day  Placebo  All interventions: Study medication was titrated during the first 6 days of treatment, maintaining a constant number of capsules until the targed dose was reached. | Anxiety: HAM-A at 1 week  Serious adverse events: Number of participants, during 4 weeks  Suicidal thoughts/attempts: Number of participants  Addiction: Withdrawal symptoms measured with Physician withdrawal checklist (PWC) during 1 week post treatment (week 5)  Drowsiness during daytime: Number of participants, during 4 weeks  Dizziness: Number of participants, during 4 weeks |
| Khan^10^, 2001, USA | n= 409  Quetiapine group:  Age in years: 44.6 (12.1)  Females: 71.6 %  Duration of symptoms, years: 15.8 (13.0)  Placebo group:  Age in years: 44.2 (10.9)  Females: 75.8%  Duration of symptoms, years: 15.0 (12.7)  Overall:  Diagnosis: Diagnosis of GAD  Outpatients (%): 100 % | RCT, multicenter (54 centers) parallel group, two arms  Funding: AstraZeneca Pharmaceuticals | Quetiapine  Flexible doses 50-300 mg  quetiapine XR + SSRI/SNRI  Quetiapine XR was initiated at 50 mg/day, with the dose increased to 150 mg/day on Day 3. At Weeks 3 or 4 a mandatory dose increase to 300 mg/day was made in patients with a CGI-S score 4 who tolerated the 150 mg/day dose. No dose increases were permitted after Week 4. Patients unable to tolerate the higher dose returned to 150 mg/day at anytime at the investigator’s discretion. Patients continued to receive the same SSRI or SNRI at the same dose as at enrollment throughout the study.  Placebo  Placebo + SSRI/SNR  Placebo tablets were identical in size, color, smell, and taste to quetiapine XR 50 mg or 300 mg tablets and packaging was identical | Anxiety: HAM-A at 1 week  Serious adverse events: Number of participants, during 14 weeks  Suicidal thoughts/attempts: Number of participants, during 10 weeks  Addiction: Withdrawal symptoms measured with Treatment discontinuation signs and symptoms,1 week post treatment (week 11)  Drowsiness during daytime: Number of participants, during 10 weeks  Dizziness: Number of participants, during 10 weeks |
| Kruger^11^, 1999, Norway, Sweden, the Netherlands | n= 135  Clomipramine group:  Age in years: 36.0 (9.5)  Females: 60.3%  Duration of symptoms, present episode, month: 21.8 (30.1)  Moclobemide group:  Age in years: 35.0 (8.9)  Females: 58.2 %  Duration of symptoms, present episode, month: 23.9 (36.1)  Overall:  Diagnosis: DSM - III - R panic disorder with or without agoraphobia  Outpatients (%): Setting unclear | RCT, multicenter (12 centers) parallel group, two arms  Funding: Hoffmann - La Roche | Antidepressant tricyclic (clomipramine)  Fixed-flexible dosage, range = 100 - 200 mg, M and SD not provided. Patients got the target doses considered effective in the treatment of panic disorders, clomipramine 150 mg day. After 4 weeks of active treatment, there was an option to increase the dose to 200 mg. During the first 4 weeks the dose could be reduced to 100 mg if the patient did not tolerate the dose due to severe side effects. No other changes were permitted.  MAO (moclobemide)  Fixed-flexible dosage, range = 300 - 600 mg, M and SD not provided. Patients got the target doses considered effective in the treatment of panic disorders, moclobemide 450 mg day. After 4 weeks of active treatment, there was an option to increase the dose to 600 mg. During the first 4 weeks the dose could be reduced to 300 mg if the patient did not tolerate the dose due to severe side effects. No other changes were permitted. | Anxiety: HAM-A at 4 week |
| Lepola^12^, 1990, Finland | n= 55  Overall:  Age in years: 37.4, SD not provided  Females: Sex not stated  Diagnosis: DMS-III panic disorder with or without agoraphobia  Duration of symptoms: at least 3 months, mean 6.4 years.  Outpatients (%):0 % | RCT, single center, parallel group, two arms  Funding: Not stated | Benzodiazepine (alpraxolam)  Flexible dosage, range = 1.5 - 8 mg, M = 4.9, SD not provided.  Antidepressant_tricyclic (imipramine)  Flexible dosage, range = 30 - 225 mg, M = 130, SD not provided | Anxiety: HAM-A at 3 week |
| Li^13^, 2016; USA | n = 23  Quetiapine group:  Age in years: 48.7 (8.92)  Females: 72.73%  Placebo group:  Age in years: 52.7 (14.81)  Females: 75 %  Overall:  Diagnosis: DSM-IV criteria major depression disorder and GAD  Duration of symptoms: No information  Outpatients (%): No information | RCT, single center, parallel group, two arms  Funding: supported by AstraZeneca Pharmaceutical Company via an Investigator Initiated study. | Quetiapine  Quetiapine XR, flexible doses between 150 and 300 mg. The study medications were started at 50 mg for day 1 and day 2, increased to 150 mg at day 3 and day 4, and finally increased to 300 mg/d at day 5 and onward. For those who could not tolerate 300 mg/d, a 50 mg decrement per week was allowed to a minimum of 150 mg/d. For those who could not tolerate 150 mg/d, they were discontinued from the study  Placebo | Anxiety: HAM-A at 1 week  Serious adverse events: Number of participants, during 8 weeks  Drowsiness during daytime: Number of participants, during 8 weeks  Dizziness: Number of participants, during 8 weeks |
| Liebowitz^14^, 1992, USA | n = 85  Phenelzine group:  Age in years: 33.7 (9.0)  Females: 68%  Duration of symptoms, years: 16.9 (10.4)  Atenolol group:  Age in years: 34.5 (9.6)  Females: 65%  Duration of symptoms, years: 11.3 (9.0)  Placebo group:  Age in years: 34.8 (7.3)  Females: 73%  Duration of symptoms, years: 16.7 (10.6)  Overall:  Diagnosis: Social phobia DSM-III criteria  Outpatients (%): 100% | RCT, single center, parallel group, three arms  Funding: Parke-Davis Phamaceutical Co now Pfeizer (phelenzine) and Stuart Pharmaceuticals (atenolol) supplied for medication | MAO (Phenelzine)  15-90 mg phenelzine  Treatment begun at 15 mg/day increased to 30 mg/day on day 4, to 45 mg on day 8, and to 60 mg on day 15. After 4 weeks, depending on clinical state and side effects, the dose could be optionally raised to 75 mg/day, and to 90 mg/day after 5 weeks.  Betablokker (Atenolol)  50-100 mg atenolol  Treatment begun at 50 mg/day given in themorning and raised to 100 mg/day if tolerated, after 2 weeks.  Placebo | Anxiety: HAM-A at 4 weeks |
| Llorca^15^, 2002, France | n = 334  Bromazepam group:  Age in years: 44.9 (11.5)  Females: 68%  Hydroxyzine group:  Age in years: 43.6 (11.7)  Females: 70.5%  Placebo group:  Age in years: 41.5 (11.9)  Females: 66,4 %  Overall:  Diagnosis: diagnosis of GAD according to DSM-IV criteria  Duration of symptoms: No information  Outpatients (%): 100% | RCT, multicenter (89 centers, general practioners), parallel group, three arms  Funding: UCB-pharma | Benzodiazepine (bromazepam)  Bromazepam, fixed doses, 6 mg  During the doubled blind period of the study the daily dose of bromazepam was 6 mg (1,5 mg in the morning and at noon and 3 mg in the evening)  Antihistamin (Hydroxyzine)  Hydroxyzine, fixed doses, 50 mg  During the doubled blind period of the study the daily dose of hydroxyzine was 50 mg (12,5 mg in the morning and at noon and 25 mg in the evening)  Placebo  All interventions: Daily medication/placebo was given as oral capsules in 3 divied doses. | Anxiety: HAM-A at 3 weeks  Serious adverse events: Number of participants, during 12 weeks  Drowsiness during daytime: Number of participants, during 12 weeks |
| Merideth^16^, 2012, USA | n = 854  Quetiapine 150 mg group:  Age in years: 38.2 (11.5)  Females: 68%  Quetiapine 300 mg group:  Age in years: 39.0 (12.6)  Females: 71%  Escitalopram group:  Age in years: 40.4 (11.6)  Females: 66%  Placebo group:  Age in years: 36.6 (12.3)  Females: 64%  Overall:  Diagnosis: DSM-IV-TR diagnosis of GAD  Duration of symptoms: No information  Outpatients (%): 100% | RCT, multicenter (64 centers), parallel group, four arms  Funding: sponsored by Astra Zeneca. Medical writing support funded by AstraZeneca. | Quetiapine 150 mg  Quetiapine XR treatment was initiated at 50 mg/day on days 1 and 2 of the randomized treatment period, and increased to 150 mg/day on days 3 and 4.  Quetiapine 300 mg  Quetiapine XR treatment was initiated at 50 mg/day on days 1 and 2 of the randomized treatment period, increased to 150 mg/day on days 3 and 4, and increased to 300 mg/day on day 5.  SSRI (escitalopram)  Escitalopram 10 mg, fixed dose  Placebo  Placebo tablets/capsules were identical in size, color, smell, and taste to their respective active treatment (quetiapine XR or escitalopram) tablets/capsules.  All interventions: To ensure blinding, packaging was identical for all treatments. All study medication was administered orally, once daily, in the evening. | Anxiety: HAM-A at 1 week  Serious adverse events: Number of participants, during 10 weeks  Addiction: Withdrawal symptoms measured with Treatment discontinuation signs and symptoms, during 2 weeks post treatment (week 8-10)  Changes in weight: Number of participants, during 10 weeks  Extrapyramidal symptoms: Number of participants, during 10 weeks  Drowsiness during daytime: Number of participants, during 10 weeks  Dizziness: Number of participants, during 10 weeks |
| Michelson^17^, 2013, USA | n = 213  Lorazepam group:  Age in years: 36.4 (10.8)  Females: 56.5%  L-759274 group:  Age in years: 38.3 (10.5)  Females: 41.1%  Placebo group:  Age in years: 41.3 (11.4)  Females: 56.3%  Overall:  Diagnosis: DSM-IV criteria for generalized anxiety disorder  Duration of symptoms: No information  Outpatients (%): No information | RCT, multicenter (6 academic and private research sites in the United States), parallel group, four arms  Funding: sponsored by Merck. | Benzodiazepine (lorazepam)  Lorazepam 1-6 mg, flexible doses. Lorazepam was initiated at 1 mg/d and could be increased to a maximum of 6 mg/d based on the investigator’s assessment of symptom response  L-759274  40 mg L-759274.  L-759274. Is an antagonist of the NK1 receptor  Placebo  . | Anxiety: HAM-A at 1 week  Serious adverse events: Number of participants, during 6 weeks  Drowsiness during daytime: Number of participants, during 6 weeks  Dizziness: Number of participants, during 6 weeks |
| Møller^18^, 200, Germany | n = 313  Overall:  Age in years: No information  Females: No information  Diagnosis: GAD according to ICD-10 code F41.1  Duration of symptoms: No information  Outpatients (%): 100% | RCT, parallel group, three arms  Funding: Not stated | Benzodiazepine (alprazolam)  Capsules contained 0.5 mg of alprazolam. On day 1, two capsules in the evening were active, and on day 2, the morning capsules also contained active medication. From day 3 onward, the final doses of 200 mg of opipramol and 2 mg of alprazolam given in four capsules were reached, whereas patients receiving placebo were only given inert capsules (days 7 to 35)  Antidepressant tricyclic (opipramol)  Capsules contained 50 mg of opripramol  On day 0 of the first 3 days of the double-blind treatment period, one of the two evening capsules contained active medication in the opipramol and alprazolam groups. On day 1, two capsules in the evening were active, and on day 2, the morning capsules also contained active medication. From day 3 onward, the final doses of 200 mg of opipramol and 2 mg of alprazolam given in four capsules were reached, whereas patients receiving placebo were only given inert capsules (days 7 to 35)  Placebo  Capsules contained placebo  All interventions: Medication was prepared in capsules of identical appearance. | Anxiety: HAM-A at 1 week  Serious adverse events: Number of participants, during 4 weeks  Fractures: Number of participants, during 4 weeks  Dizziness: Number of participants, during 4 weeks |
| Nguyen^19^, 2006 | n = 191  Lorazepam group:  Age in years: 42.0 (13.1)  Females: 69.8%  Etifoxine group:  Age in years: 44.0 (13.4)  Females: 62.4%  Overall:  Diagnosis: Adjustment Disorder With Anxiety (ADWA) (DSM IV)  Duration of symptoms: No information  Outpatients (%): 100% | RCT, multicenter (36 centers general practioners), parallel group, three arms  Funding: Supported by Biocodex, Compiègne, France | Benzodiazepine (lorazepam)  lorazepam up to 2 mg/day, usual dosage 0.5–1 mg by day for lorazepam  Anxiolytic (etifoxine)  Etifoxine up to 150 mg/day, usual dosage 50 mg 3 times a day | Anxiety: HAM-A at 1 week |
| Noyes^20^,1996, USA, Australia | n = 241  Overall:  Age in years: 36.6 (10.5)  Females: 65%  Diagnosis: DSM-III panic disorder or agoraphobia with panic attacks  Duration of symptoms, years: 9.1 (10.1)  Outpatients (%): 100% | RCT, multicenter (2 centers in USA and Australia), parallel group, three arms  Funding:  Supported by a grant from the Upjohn Company | Benzodiazepine (diazepam)  Diazepam, capusels containing 10 mg, flexible dosage, range = 10 - 100 mg, M = 43, SD not provided.  Benzodiazepine (alprazolam)  Alprazolam, capsules containing 1 mg, flexible dosage, range = 1 - 10 mg, M = 4.9, SD not provided.  Placebo  All interventions:  Capsules were administered in divided doses four times daily. Medication was gradually increased according to a standardized schedule until maximum benefits was achieved or dose limiting side-effects appeared. an effort was made to achiede a dose of 6 capsules per day by the end of the 3 week. but the maximum allowed dose was 10 capsules per day. | Anxiety: HAM-A at 4 week  Serious adverse events: Number of participants, during 8 weeks  Changes in weight: Number of participants, during 8 weeks  Drowsiness during daytime: Number of participants, during 8 weeks |
| Pande^21^, 2003, USA | n = 276  Lorazepam group:  Age in years: 33.9 (9.7)  Females: 63.2%  Pregabalin 150 mg:  Age in years: 37.9 (11.8)  Females: 49.3%  Pregabalin 600 mg:  Age in years: 35.5 (11.2)  Females: 57.1%  Placebo:  Age in years: 35.7 (11.5)  Females: 68.1%  Overall:  Diagnosis: generalized anxiety disorder according to DSM-IV criteria  Duration of symptoms, years: No information  Outpatients (%): 100% | RCT, multicenter (5 centers), parallel group, four arms  Funding: Not stated | Benzodiazepine (lorazepam)  Lorazepam, 6 mg/day (2 mg t.i.d).  Pregabalin 150 mg  Pregabalin, 150 mg/day (50 mg t.i.d.).  Pregabalin 600 mg  600 mg/day (200 mg t.i.d.).  Placebo  All interventions: Study medication was titrated during the first 6 days of double-blind treatment. On day 1, subjects received one-sixth of the randomly assigned dose, which was then increased daily until the targeted dose was reached. | Anxiety: HAM-A at 1 week  Serious adverse events: Number of participants, during 4 weeks  Addiction: Withdrawal symptoms measured with Physician withdrawal checklist (PWC), during a taper week (week 5).  Drowsiness during daytime: Number of participants, during 4 weeks  Dizziness: Number of participants, during 4 weeks |
| Razavi^22^, 1999, Belgium | n = 27  Trazodone group:  Age in years, median: median age 56.5 years (range 33 - 71 years)  Clorazepate group:  Age in years, median: median age 56.5 years (range 33 - 71 years)  Overall:  Females: 100%  Diagnosis: Female breast cancer patients with fulfilment of DSM-III-R criteria for the diagnosis of adjustment disorders with anxiety or depressed mood and/or mixed disturbance of emotion and conduct.  Duration of symptoms, years: No information  Outpatients (%): 100% | RCT, single center, parallel group, two arms  Funding: supported by Searle Continental Pharma Inc., Brussels, Belgium | Antidepressant (Trazodone)  Trazodone 150 mg. The dosing schedule was one capsule (containing trazodone 50 mg) on day 1 and day 2, two capsules on day 3 and day 4, and three capsules per day from day 5 to day 28  Benzodiazepine (Clorazepate)  Clorazepate 10-30 mg. The dosing schedule was one capsule (clorazepate 10 mg) on day 1 and day 2, two capsules on day 3 and day 4, and three capsules per day from day 5 to day 28. | No relevant outcomes reported for our interventions of interrest |
| Rickels^23^, 2005, USA | n = 454  Alprazolam group:  Age in years: 40 (12)  Females: 66%  Pregabalin 300 mg group:  Age in years: 38 (10)  Females: 64%  Pregabalin 450 mg group:  Age in years: 38 (12)  Females: 59%  Pregabalin 600 mg group:  Age in years: 39 (12)  Females: 67%  Placebo:  Age in years: 41 (12)  Females: 63%  Overall:  Diagnosis: DSM-IV criteria for GAD  Duration of symptoms, years: No information  Outpatients (%): 100% | RCT, multicenter (29 centers), parallel group, five arms  Funding: supported by Pfizer Inc, New York, NY. | Benzodiazepine (alprazolam)  Fixed dosages of pregabalin 300 mg  Pregabalin 300 mg  Fixed dosages of pregabalin 450 mg  Pregabalin 450 mg  Fixed dosages of pregabalin 600 mg  Pregabalin 600 mg  Fixed dosages  Placebo  All interventions: Pregabalin treatment was initiated at 300 mg/d for all 3 dosages; for patients assigned to 450 and 600 mg of pregabalin, the dosage was titrated to 450 mg/d on day 4; and for those assigned to 600 mg of pregabalin, the dosage was titrated to 600 mg/d on day 7. Study drug was administered in divided doses using a 3 times a day schedule. | Anxiety: HAM-A at 1 week  Serious adverse events: Number of participants, during 6 weeks  Addiction: Withdrawal symptoms measured with Physician withdrawal checklist (PWC), during a taper week (week 5).  Drowsiness during daytime: Number of participants, during 6 weeks  Dizziness: Number of participants, during 6 weeks  Cardial side-effects: Number of participants, during 6 weeks |
| Rocca^24^, 1997, Italy | n = 81  Delorazepam group:  Age in years: 37.5 (11.1)  Duration of symptoms, years: Age at onset 30.0 (6.6)  Paroxetine group:  Age in years: 35.3 (9.3)  Duration of symptoms, years: Age at onset 28 5 (7.4)  Imipramine group:  Age in years: 37.6 (9.3)  Duration of symptoms, years: Age at onset 29.4 (6.7)  Overall:  Diagnosis: DSM-IV criteria for GAD  Females: 57 %  Duration of symptoms, years: For 60% of the patients the duration of the current episode was more than 1 year  Outpatients (%): 100% | RCT, single center, parallel group, three arms  Funding: Not stated | Benzodiazepine (delorazepam)  Flexible doses, range 3-6 mg (daily), mean daily dose 74.2 mg, SD 1.1  SSRI (Paroxetine)  20 mg daily dose  Antidepressant tricyclic (imipramine)  Flexible doses, range 50-100 mg daily, mean daily dose 75 mg, SD 16  All interventions: The optimal dose was reached within 1 week | Anxiety: HAM-A at 2 weeks |
| Schweizer^25^, 1993, USA | n = 106  Overall:  Age in years: 33 (7)  Females: 75 %  Diagnosis: DSM - III panic disorder  Duration of symptoms, years: 84% had suffered from panic disorder at least 1 year and 59% for at least 3 years.  Outpatients (%): Probably outpatients | RCT, parallel group, three arms  Funding: supported by research grant from the Upjohn Co, Kalamazoo, Mich and by Publich Health Service Grant | Benzodiazepine (alprazolam)  Flexible dosage, range = 2 - 10 mg, M = 5.4, SD = 2.1. Capsules containing 1 mg of alprazolam  Antidepressant tricyclic (Imipramine)  Flexible dosage, range = 50 - 250 mg, M = 152, SD = 65.  Capsules containing 25 mg. of imipramine  Placebo  Capsules containing lactose filler as placebo  All interventions: Treatment was initiated at one capsule in the evening. Stepwise increasaes in daily doses were mase every 3 to 4 days according to following schedule: two capsules per day for 3 days, 3 capsuels per day for 4 days, four capsules per day for 4 days, 5 capsules for 4 days and so on as tolerated. Every effort was made to increase the dosage of all patients to a minimum of 6 capsuels per day (6 mg alprazolam/150 mg of imipramine). The maximum permitted dose was 10 capsules (10 mg alprazolam/ 250 mg imipramine). when patients reported advesre effects, the dose titration was slowed, or if necessary, the daily dose was reduced. Medications were taken 4 times daily. Patients were allowed to remain in the study while taklen daily doses af low as one pill per day. | Anxiety: HAM-A at 4 weeks  Addiction: Withdrawal symptoms, number of participants with withdrawal symptoms  Drowsiness during daytime: Number of participants, during 4 weeks |
| Song^26^, 2017, China | n = 156  Diazepam group  Age in years: 47.94 (12.10)  Females: 57.7%  Placebo group (only paroxetine)  Age in years: 50.60 ± 12.84  Females: 60.5 %  Chinese herbs group  Age in years: 48.96 (12.87)  Females: 56 %  Overall:  Diagnosis: GAD based on DSM-V  Duration of symptoms: No information  Outpatients (%): 0% | RCT, single center, parallel group, three arms  Funding: supported by National Natural Science Foundation of China [Grant no. 81601183] and Science and Technology Council of Hangzhou [Grant nos. 20160533B28 and 20140733Q49]. | Benzodiazepine (Diazepam + paroxetine)  7.5 mg diazepam + paroxetine 20-60 mg.  2.5 mg of diazepam three times daily  Placebo (only paroxetine)  Paroxetine 20-60 mg  Chinese herbs (MSZRT) + paroxetine  MSZRT 400 ml + paroxetine 20-60 mg  Daily dose of MSZRT formula for each patient comprised Suanzaoren (Semen Zizyphi Spinosae) 15 g, Zhimu (Rhizoma Anemarrhena) 12 g, Fuling (Sclerotium Poriae Cocos) 15 g, Chuanxiong (Radix Ligustici Chuanxiong) 10 g, Zhizi (Gardenia jasminoides fruit) 10 g, Dandouchi (Fermented Soybean) 6 g, Chanyi (periostracum cicada) 6 g, and Zhigancao (Radix Glycyrrhizae) 6 g. All herbs were purchased from Medicinal Materials Co. Ltd. (Lin’an City, Zhejiang Province, China). They were mixed and prepared as 400 ml of decoction solution according to traditional methods and packed into two bags.  All interventions: Subjects in three groups took paroxetine 20 mg/day half an hour after breakfast in the first week. From second week, they were allowed to increase paroxetine dose. The maximum dose during the study period was 60 mg/day if judged clinically necessary by the investigator. | Anxiety: HAM-A at 1 week  Serious adverse events: Number of participants, during 4 weeks  Drowsiness during daytime: Number of participants, during 4 weeks  Dizziness: Number of participants, during 4 weeks |
| Stein^27^, 2008, Finland and South Africa | n = 121  Overall:  Age in years: 41.7 (12.2)  Females: 68.6 %  Diagnosis: Primary diagnosis of GAD DSM-IV  Duration of symptoms, years: 9.6 (10.5)  Outpatients (%): 100 % | RCT, multicenter (Finland 5 centers, South Africa 6 centers) parallel group, two arms  Funding: Not stated | Agomelatin  Agomelatin 25-50 mg. Dosage could be increased from 25-50 mg daily based on insufficient improvement from week 2 weeks onward.  Placebo | Anxiety: HAM-A at 2 weeks  Serious adverse events: Number of participants, during 12 weeks  Addiction: Withdrawal symptoms measured with Discontinuation-Emergent Signs and Symptoms (DESS) Scale after 12 weeks  Dizziness: Number of participants, during 12 weeks |
| Stein^28^, 2015, South Africa | n = 201  Alprazolam group  Age in years: 38.9 (12.8)  Females: 70.3%  Etifoxine group:  Age in years: 40.0 (11.8)  Females: 76.0 %  Overall:  Diagnosis: Adjustment disorder with anxiety (ADWA) as defined by the DSM-IV  Duration of symptoms: No information  Outpatients (%): 100% | RCT, multicenter (17 centers) parallel group, two arms  Funding: Sponsored by Biocodex, Gentilly, Franc | Benzodiazepine (alprazolam)  alprazolam 1.5 mg/day  Anxiolytic (Etifoxine)  Etifoxine 150 mg/day  All interventions: Study drug was to be taken daily for 28 days (one capsule in the morning, at noon and in the evening), at usual dosages (1.5 mg/day for alprazolam and 150 mg/day for etifoxine), in conformity with the summary of product characteristics (SmPC) of the two drugs. Study treatments were presented as capsules identical in their appearance | Anxiety: HAM-A at 1 week |
| Stein^29^, 2017, Finland, Russia, Poland, Slovakia, Ukraine | n = 412  Agomelatin 10 mg group:  Age in years: 43.6 (13.4)  Females: 67.9%  Duration of symptoms, years: 3.7 (SD not provided)  Agomelatin 25 mg group:  Age in years: 44.1 (15.2)  Females: 71.9 %  Duration of symptoms, years: 4.2 (SD not provided)  Placebo group:  Age in years: 44.1 (13.1)  Females: 63.4 %  Duration of symptoms, years: 3.6 (SD not provided)  Overall:  Diagnosis: Primary diagnosis of GAD according to DSM-IV-TR criteria.  Outpatients (%): 100 % | RCT, multicenter (Finland (6 centres), Russia (6 centres), Poland (9 centres), Slovakia (6 centres), and Ukraine (8 centres)) parallel group, three arms  Funding: sponsored by Servier | Agomelatin  Agomelatin 10 mg in the evening for 12 weeks  Agomelatine 25 mg in the evening for 12 weeks  Placebo  Placebo in the evening for 12 weeks.  All interventions: All treatments were identically labeled | Anxiety: HAM-A at 1 week  Serious adverse events: Number of participants, during 12 weeks  Drowsiness during daytime: Number of participants, during 12 weeks  Dizziness: Number of participants, , during 12 weeks |
| Taylor^30^, 1990, USA | n = 79  Alprazolam group  Age in years: 35.0 (SD not provided)  Females: 81%  Imipramine group:  Age in years: 31.1 (SD not provided)  Females: 65.9 %  Placebo group:  Age in years: 34.9 (SD not provided)  Females: 65.1 %  Overall:  Diagnosis: Panic disorder  Duration of symptoms: No information  Outpatients (%): 100% | RCT, parallel group, three arms  Funding: Supported in part by NIMH grant 40118 and by a giN from the Up- john Company. | Benzodiazepine (alprazolam)  Flexible dosage; range = 1 - 8 mg, mean 3.7  Antidepressant tricyclic (Imipramine)  Flexible dosage; range = 30 - 270 mg, mean = 147  Placebo  Identical capsules up to 10 tablets per day  All interventions: Medications were dispensed in identical capsules of placebo, alprazolam 1 mg or imipramine 30 mg. Medications were uncreased until patients were free of panic attacksm suffered from unplessant side effects or were taking 10 tablets per day. | Anxiety: HAM-A at 1 week |
| Van Vliet^31^ ,1992, The Netherlands | n = 30  Overall:  Age in years, mean (SEM): 32.8 (2.0)  Females: 70%  Diagnosis: Social phobia according to DSM-III-R criteria  Duration of symptoms, years: 12.4 (2.5)  Outpatients (%): 100% | RCT, single center, parallel group, two arms  Funding: Not stated | MAO-I (Brofaromine)  Brofaromine 150 mg daily  The dose of brofaromine was gradually increased from 50 to 150 mg daily (75 mg b.i.d.) in 3 weeks. If patients judged themselves to be improved they could continue their medication under doubleblind conditions in a follow-up period which lasted another 12 weeks.  Placebo | Anxiety: HAM-A at 1 week |
| Van Vliet^32^, 1997, The Netherlands | n = 30  Buspirone group:  Age in years: 41.6 (8.1)  Duration of symptoms, years: age at onset 19.4 (7.3). Duration of ilness 22.2 years (4.7)  Placebo group:  Age in years: 32.9 (9.6)  Duration of symptoms, years: age at onset 17.1 (4.7). Duration of ilness 15.8 years (9.7)  Overall:  Females: 36.7 %  Diagnosis: Social phobia, specific or generalized subtype, according to DSM-IV cirteria  Outpatients (%): 100% | RCT, single center, parallel group, two arms  Funding: Not stated | Anxiolytic (Buspirone)  Buspirone 30 mg daily, the dose was gradually increased from 15 mg in the first week to 30 mg from the third week on (10 mg t.i.d.)  Placebo | Anxiety: HAM-A at 1 week |
| Versiani^33^, 1992, Switzerland | n = 78  Overall  Age in years: No information  Females: men and women, no further information  Diagnosis: DSM-III-R criteria for social phobia  Duration of symptoms: No information  Outpatients (%):No information | RCT, parallel group, three arms  Funding: Not stated | MAO-I (Moclobemide)  Flexible doses, 100-600 mg, the mean (s.d.) daily doses were 580.7 (55.6) mg/day (end of phase 1, 8 weeks)  MAO-I (Phenelzine)  Flexible doses, 30-90 mg. the mean (s.d.) daily doses were: phenelzine group, 67.5 (15.0) mg/day (end of phase 1, 8 weeks)  Placebo  Capsules of identical appearance. mean (s.d.) daily doses were: placebogroup,5.9(0.4) (end of phase 1, 8 weeks)  All interventions: Medication was provided in capsules of identical appearance containing moclobemide 100 mg, phenel´zine 15 mg or placebo. The initial dose was one capsule twice daily, morning, and afternoon; if tolerated, this dose was increased on day 4 to four capsules a day - two in the morning, one in the afternoon, and one at bedtime. This dose was maintained until the end of week 4. At week5, if the dose was tolerated, it was increased again to five capsules per day - two in the morning, two in the afternoon, and one at bedtime. At week 6, there was a further option to increase the dose to two capsules thrice daily; attempts were made to reach this maximum dose (600 mg/day moclobemide, 90 mg/day phenelzine) | Anxiety: HAM-A at 4 weeks |
| Versiani^34^, 1997, Brasil | n = 60  Bromazepam group:  Age in years: 34.7 (9.8)  Females: 40%  Placebo group:  Age in years: 38.7 (10)  Females: 30%  Overall:  Diagnosis: social phobias DSM-III criteria  Duration of symptoms: No information  Outpatients (%): No information | RCT, single center, parallel group, two arms  Funding: Not stated | Benzodiazepine (bromazepam)  Bromazepam, flexible doses 9-27 mg. Doses started at 9 mg (3 mg. three times a day), and increased by 3 mg. every week until week 7 were the doses were 27 mg, (9 mg three times a day). Doses were decreased if not tolerated. Efforts were made to attain the maximum dose mean final dose was 21 mg  Placebo  Tablets of identical appearance | Anxiety: HAM-A at 4 weeks  Function of daily living/disability: Sheehan Disability Scale, work dimention, social dimention and family dimention at 4 weeks  Drowsiness during daytime: Number of participants, during 12 weeks |

**References**

1) CNCPS, Drug treatment of panic disorder. Comparative efficacy of alprazolam, imipramine, and placebo. Cross-National Collaborative Panic Study, Second Phase Investigators. Br J Psychiatry 1992;160:191-202; discussion 202-5.
2) Amore M, Magnani K, Cerisoli M, Casagrande C, Ferrari G. Panic disorder. A long-term treatment study: fluoxetine vs imipramine. . Human Psychopharmacology Clinical and Experimental 1999;14(6):429-34.
3)Ansseau M, Bataille M, Briole G, de Nayer A, Fauchere, P. A., et al. Controlled comparison of tianeptine, alprazolam and mianserin in the treatment of adjustment disorders with anxiety and depression. Human Psychopharmacology: Clinical and Experimental 1996;11(4):293-298.
4) Bakish D, Saxena BM, Bowen R, D'Souza J. Reversible monoamine oxidase-A inhibitors in panic disorder. Clin Neuropharmacol 1993;16 Suppl 2:S77-82.
5) De Leo D. Treatment of adjustment disorders: a comparative evaluation. Psychol Rep 1989;64(1):51-54.
6) Den Boer JA, Westenberg HG. Effect of a serotonin and noradrenaline uptake inhibitor in panic disorder; a double-blind comparative study with fluvoxamine and maprotiline. Int Clin Psychopharmacol 1988;3(1):59-74.
7) De Wit S, Cremers L, Hirsch D, Zulian C, Clumeck N, Kormoss N. Efficacy and safety of trazodone versus clorazepate in the treatment of HIV-positive subjects with adjustment disorders: a pilot study. J Int Med Res 1999;27(5):223-232.
8) EMEA. Scientific discussion from EMEA.Product name: LYRICA Product no: MEA/H/C/000546/II/0004 . Opdateret: 20. march 2005.28. februar 2022.
9) Feltner DE, Crockatt JG, Dubovsky SJ, Cohn CK, Shrivastava RK, Targum SD, et al. A randomized, double-blind, placebo-controlled, fixed-dose, multicenter study of pregabalin in patients with generalized anxiety disorder. J Clin Psychopharmacol 2003;23(3):240-249.
10) Khan A, Atkinson S, Mezhebovsky I, She F, Leathers T, Pathak S. Extended Release Quetiapine Fumarate (Quetiapine XR) as Adjunct Therapy in Patients with Generalized Anxiety Disorder and a History of Inadequate Treatment Response: A Randomized, Double-Blind Study. Psychopharmacol Bull 2011;44(2):5-31.
11) Krüger MB, Dahl AA. The efficacy and safety of moclobemide compared to clomipramine in the treatment of panic disorder. Eur Arch Psychiatry Clin Neurosci 1999;249 Suppl 1:S19-24.
12) Lepola U, Heikkinen H, Rimon RR, P. Clinical evaluation of alprazolam in patients with panic disorder; a double-blind comparison with imipramine. . Human Psychopharmacology 1990;5:159-63 1990;5:159-63.
13) Li R, Wu R, Chen J, Kemp DE, Ren M, Conroy C, et al. A Randomized, Placebo-Controlled Pilot Study of Quetiapine-XR Monotherapy or Adjunctive Therapy to Antidepressant in Acute Major Depressive Disorder with Current Generalized Anxiety Disorder. Psychopharmacol Bull 2016;46(1):8-23.
14) Liebowitz MR, Schneier F, Campeas R, Hollander E, Hatterer J, Fyer A, et al. Phenelzine vs atenolol in social phobia. A placebo-controlled comparison. Arch Gen Psychiatry 1992;49(4):290-300.
15) Llorca PM, Spadone C, Sol O, Danniau A, Bougerol T, Corruble E, et al. Efficacy and safety of hydroxyzine in the treatment of generalized anxiety disorder: a 3-month double-blind study. J Clin Psychiatry 2002;63(11):1020-1027.
16) Merideth C, Cutler AJ, She F, Eriksson H. Efficacy and tolerability of extended release quetiapine fumarate monotherapy in the acute treatment of generalized anxiety disorder: a randomized, placebo controlled and active-controlled study. Int Clin Psychopharmacol 2012;27(1):40-54.
17) Michelson D, Hargreaves R, Alexander R, Ceesay P, Hietala J, Lines C, et al. Lack of efficacy of L-759274, a novel neurokinin 1 (substance P) receptor antagonist, for the treatment of generalized anxiety disorder. Int J Neuropsychopharmacol 2013;16(1):1-11.
18) Möller HJ, Volz HP, Reimann IW, Stoll KD. Opipramol for the treatment of generalized anxiety disorder: a placebo-controlled trial including an alprazolam-treated group. J Clin Psychopharmacol 2001;21(1):59-65.
19) Nguyen N, Fakra E, Pradel V, Jouve E, Alquier C, Le Guern M, et al. Efficacy of etifoxine compared to lorazepam monotherapy in the treatment of patients with adjustment disorders with anxiety: A doubleblind controlled study in general practice. Human Psychopharmacology: Clinical and Experimental 2006;21:139-149.
20) Noyes R,Jr, Burrows GD, Reich JH, Judd FK, Garvey MJ, Norman TR, et al. Diazepam versus alprazolam for the treatment of panic disorder. J Clin Psychiatry 1996;57(8):349-355.
21) Pande AC, Crockatt JG, Feltner DE, Janney CA, Smith WT, Weisler R, et al. Pregabalin in generalized anxiety disorder: a placebo-controlled trial. Am J Psychiatry 2003;160(3):533-540.
22) Razavi D, Kormoss N, Collard A, Farvacques C, Delvaux N. Comparative study of the efficacy and safety of trazodone versus clorazepate in the treatment of adjustment disorders in cancer patients: a pilot study. J Int Med Res 1999;27(6):264-272.
23) Rickels K, Pollack MH, Feltner DE, Lydiard RB, Zimbroff DL, Bielski RJ, et al. Pregabalin for treatment of generalized anxiety disorder: a 4-week, multicenter, double-blind, placebo-controlled trial of pregabalin and alprazolam. Arch Gen Psychiatry 2005;62(9):1022-1030.
24) Rocca P, Fonzo V, Scotta M, Zanalda E, Ravizza L. Paroxetine efficacy in the treatment of generalized anxiety disorder. Acta Psychiatr Scand 1997;95(5):444-450.
25) Schweizer E, Rickels K, Weiss S, Zavodnick S. Maintenance drug treatment of panic disorder. I. Results of a prospective, placebo-controlled comparison of alprazolam and imipramine. Arch Gen Psychiatry 1993;50(1):51-60.
26) Song M, Hu L, Liu W, Liu Y, Tao X, Wang T, et al. Modified Suanzaorentang Had the Treatment Effect for Generalized Anxiety Disorder for the First 4 Weeks of Paroxetine Medication: A Pragmatic Randomized Controlled Study. Evid Based Complement Alternat Med 2017;2017:8391637.
27) Stein DJ, Ahokas AA, de Bodinat C. Efficacy of agomelatine in generalized anxiety disorder: a randomized, double-blind, placebo-controlled study. J Clin Psychopharmacol 2008;28(5):561-566.
28) Stein DJ. Etifoxine versus alprazolam for the treatment of adjustment disorder with anxiety: a randomized controlled trial. Adv Ther 2015;32(1):57-68.
29) Stein DJ, Ahokas A, Jarema M, Avedisova AS, Vavrusova L, Chaban O, et al. Efficacy and safety of agomelatine (10 or 25 mg/day) in non-depressed out-patients with generalized anxiety disorder: A 12-week, double-blind, placebo-controlled study. Eur Neuropsychopharmacol 2017;27(5):526-537.
30) Taylor CB, Hayward C, King R, Ehlers A, Margraf J, Maddock R, et al. Cardiovascular and symptomatic reduction effects of alprazolam and imipramine in patients with panic disorder: results of a double-blind, placebo-controlled trial. J Clin Psychopharmacol 1990;10(2):112-118.
31) van Vliet IM, den Boer JA, Westenberg HG. Psychopharmacological treatment of social phobia: clinical and biochemical effects of brofaromine, a selective MAO-A inhibitor. Eur Neuropsychopharmacol 1992;2(1):21-29.
32) van Vliet IM, den Boer JA, Westenberg HG, Pian KL. Clinical effects of buspirone in social phobia: a double-blind placebo-controlled study. J Clin Psychiatry 1997;58(4):164-168.33)
33) Versiani M, Nardi AE, Mundim FD, Alves AB, Liebowitz MR, Amrein R. Pharmacotherapy of social phobia. A controlled study with moclobemide and phenelzine. Br J Psychiatry 1992;161:353-360.
34) Versiani, M., Nardi AE, Figueira I, Mendlowicz M, Marques C. Double-blind placebo controlled trial with bromazepam in social phobia.
. J bras psiquiatr 1997;46(3):167-171

**Supplementary Table S2.** List of excluded articles after full text-review.

| Study (author and year) | Reference | Reason for exclusion |
| --- | --- | --- |
| Altmann 2020 | Altmann, Helene; Stahl, Sarah T.; Gebara, Marie Anne; Lenze, Eric J.; Mulsant, Benoit H.; Blumberger, Daniel M.; Reynolds, Charles F.,3rd; Karp, Jordan F.Coprescribed Benzodiazepines in Older Adults Receiving Antidepressants for Anxiety and Depressive Disorders: Association With Treatment Outcomes The Journal of clinical psychiatry 2020;81(6) | Wrong intervention |
| Amodeo 2012 | Amodeo, L.; Castelli, L.; Leombruni, P.; Cipriani, D.; Biancofiore, A.; Torta, R. Slow versus standard up-titration of paroxetine for the treatment of depression in cancer patients: a pilot study. Supportive care in cancer : official journal of the Multinational Association of Supportive Care in Cancer 2012;20(2):375-384 | Wrong comparator, Compares one titration method with another - same drug |
| Amore 1999 | Amore M, Magnani K, Cerisoli M, Ferrari G.  Short-term and long-term evaluation of selective serotonin reuptake inhibitors in the treatment of panic disorder: fluoxetine vs citalopram. Human Psychopharmacology: Clinical and Experimental 1999;14(6):435-40. | Wrong intervention |
| Asakura 2007 | Asakura, S.; Tajima, O.; Koyama, T. Fluvoxamine treatment of generalized social anxiety disorder in Japan: a randomized double-blind, placebo-controlled study. The international journal of neuropsychopharmacology 2007;10(2):263-274 | Wrong intervention, SSRI |
| Bakish 1996 | Bakish, D.; Hooper, C. L.; Filteau, M. J.; Charbonneau, Y.; Fraser, G.; West, D. L.; Thibaudeau, C.; Raine, D.  A double-blind placebo-controlled trial comparing fluvoxamine and imipramine in the treatment of panic disorder with or without agoraphobia. Psychopharmacology bulletin 1996;32(1):135-141 | Wrong outcomes, no data regarding critical outcomes within 4 weeks  Same study as Nair 1996 |
| Bandelow 2010 | Bandelow, B.; Chouinard, G.; Bobes, J.; Ahokas, A.; Eggens, I.; Liu, S.; Eriksson, H.  Extended-release quetiapine fumarate (quetiapine XR): a once-daily monotherapy effective in generalized anxiety disorder. Data from a randomized, double-blind, placebo- and active-controlled study  The international journal of neuropsychopharmacology 2010;13(3):305-320 | Wrong outcomes, no data regarding critical outcomes within 4 weeks |
| Barnett 2002 | Barnett, S. D.; Kramer, M. L.; Casat, C. D.; Connor, K. M.; Davidson, J. R. Efficacy of olanzapine in social anxiety disorder: a pilot study  Journal of psychopharmacology (Oxford, England) 2002;16(4):365-368 | Wrong outcomes, no data regarding critical outcomes within 4 weeks |
| Blanco 2010 | Blanco, C.; Heimberg, R. G.; Schneier, F. R.; Fresco, D. M.; Chen, H.; Turk, C. L.; Vermes, D.; Erwin, B. A.; Schmidt, A. B.; Juster, H. R.; Campeas, R.; Liebowitz, M. R. A placebo-controlled trial of phenelzine, cognitive behavioral group therapy, and their combination for social anxiety disorder  Archives of General Psychiatry 2010;67(3):286-295  2010 | Wrong outcomes, no data regarding critical outcomes within 4 weeks |
| Bystritsky 1994 | Bystritsky, A.; Rosen, R. M.; Murphy, K. J.; Bohn, P.; Keys, S. A.; Vapnik, T. Double-blind pilot trial of desipramine versus fluoxetine in panic patients. Anxiety 1994;1(6):287-290 | Wrong outcomes, no data regarding critical outcomes within 4 weeks |
| Careri 2015 | Careri, J. M.; Draine, A. E.; Hanover, R.; Liebowitz, M. R. A 12-Week Double-Blind, Placebo-Controlled, Flexible-Dose Trial of Vilazodone in Generalized Social Anxiety Disorder. The primary care companion for CNS disorders 2015;17(6):10.4088/PCC.15m01831 | Wrong intervention, SSRI |
| Christensen 2019 | Christensen, M C; Loft, H; Florea, Ioana; McIntyre, Roger S.  Efficacy of vortioxetine in working patients with generalized anxiety disorder. CNS spectrums 2019;24(2):249-257 | Wrong intervention, |
| Connor 1998 | Connor, K. M.; Davidson, J. R.; Potts, N. L.; Tupler, L. A.; Miner, C. M.; Malik, M. L.; Book, S. W.; Colket, J. T.; Ferrell, F. Discontinuation of clonazepam in the treatment of social phobia  Journal of clinical psychopharmacology 1998;18(5):373-378 | Wrong outcomes, no data regarding critical outcomes within 4 weeks |
| Davidson 1993 | Davidson, J. R.; Potts, N.; Richichi, E.; Krishnan, R.; Ford, S. M.; Smith, R.; Wilson, W. H. Treatment of social phobia with clonazepam and placebo  Journal of clinical psychopharmacology 1993;13(6):423-428 | Wrong outcomes, no data regarding critical outcomes within 4 weeks |
| Den Boer 1990 | Den Boer, J. A.; Westenberg, H. G. Serotonin function in panic disorder: a double blind placebo controlled study with fluvoxamine and ritanserin. Psychopharmacology 1990;102(1):85-94 | Wrong intervention, SSRI |
| Durgan 2016 | Durgam, S; Gommoll, C; Forero, G; Nunez, R; Tang, X; Mathews, M; Sheehan, D V. Efficacy and Safety of Vilazodone in Patients With Generalized Anxiety Disorder: A Randomized, Double-Blind, Placebo-Controlled, Flexible-Dose Trial. The Journal of clinical psychiatry 2016;77(12):1687-1694 | Wrong intervention |
| Fahlén 1995 | Fahlén, T.; Nilsson, H. L.; Borg, K.; Humble, M.; Pauli, U. Social phobia: the clinical efficacy and tolerability of the monoamine oxidase -A and serotonin uptake inhibitor brofaromine. A double-blind placebo-controlled study  Acta Psychiatrica Scandinavica 1995;92(5):351-358 | Wrong outcomes, no data regarding critical outcomes within 4 weeks |
| Furmark 2005 | Furmark, T.; Appel, L.; Michelgård, A.; Wahlstedt, K.; Ahs, F.; Zancan, S.; Jacobsson, E.; Flyckt, K.; Grohp, M.; Bergström, M.; Pich, E. M.; Nilsson, L. G.; Bani, M.; Långström, B.; Fredrikson, M.Cerebral blood flow changes after treatment of social phobia with the neurokinin-1 antagonist GR205171, citalopram, or placebo. Biological psychiatry 2005;58(2):132-142 | Wrong intervention, SSRI |
| Fava 1989 | Fava, M.; Rosenbaum, J. F.; MacLaughlin, R. A.; Tesar, G. E.; Pollack, M. H.; Cohen, L. S.; Hirsch, M.  Dehydroepiandrosterone-sulfate/cortisol ratio in panic disorder. Psychiatry research 1989;28(3):345-350 | Wrong outcomes, no data regarding critical outcomes within 4 weeks  Same study as Labbate 1994, Pollack 1993, Tesar 1987, Tesar 1991, |
| Gao 2009 | Gao, R.; Zhao, Y.  高润利,赵艳艳.米氮平与劳拉西泮治疗广泛性焦虑症的疗效观察[J].四川精神卫生  2009;(1):59-59,63 | Language |
| Garvey 1989 | Garvey, M.; Noyes, R.,Jr; Cook, B.; Tollefson, G.  The relationship of panic disorder and its treatment outcome to 24-hour urinary MHPG levels. Psychiatry research 1989;30(1):53-61 | Wrong outcomes, no data regarding critical outcomes within 4 weeks |
| **Gentil 1993** | Gentil, V.; Lotufo-Neto, F.; Andrade, L.; Cordás, T.; Bernik, M.; Ramos, R.; Maciel, L.; Miyakawa, E.; Gorenstein, C. Clomipramine, a better reference drug for panic/agoraphobia. I. Effectiveness comparison with imipramine  Journal of psychopharmacology (Oxford, England) 1993;7(4):316-324 | Wrong outcomes, no data regarding critical outcomes within 4 weeks  Same study as Marcourakis 1993 |
| **Goddard 2015** | Goddard, A W.; Mahmud, W; Medlock, C; Shin, Y W; Shekhar, A.. A controlled trial of quetiapine XR coadministration treatment of SSRI-resistant panic disorder. Annals of general psychiatry 2015;14(Journal Article):26 | Wrong outcomes, no data regarding critical outcomes within 4 weeks |
| Gommoll 2015 | Gommoll, C; Forero, G; Mathews, M; Nunez, R; Tang, X; Durgam, S; Sambunaris, A.  Vilazodone in patients with generalized anxiety disorder: A double-blind, randomized, placebo-controlled, flexible-dose study. International clinical psychopharmacology 2015;30(6):297-306 | Wrong intervention |
| Gong 2016 | Gong Y.; Lin L. Effects of mirtazapine combined with standard medical treatment in patients with functional dyspepsia accompanying psychological distress: A randomized, double-blind, placebo-controlled trial. Gastroenterology 2016;150(4):S43 | Wrong outcomes, no data regarding critical outcomes within 4 weeks |
| GSK Clinical Studies Register 2018 | GSK Clinical Studies Register. A double-blind, multicenter, flexible-dose study of paroxetine, alprazolam and placebo in the treatment of panic disorder. 2018;17. februar 2022(Web Page): | Wrong outcomes, no data regarding critical outcomes within 4 weeks |
| GSK, Glaxo Smith Kline 1993 | GSK, Glaxo Smith Kline. A double-blind placebo controlled comparative study of paroxetine and clomipramine in the treatment of panic disorder. GSK-ClinicalStudy Register.  11/12 1993;(Web Page):1993 11/12  https://www.gsk-studyregister.com/en/trial-details/?id=29060/187 | Wrong outcomes, no data regarding critical outcomes within 4 weeks  Same study as **Lecrubier 1997a, Lecrubier 1997b** |
| Guo 2009 | Guo, H.; Ren, Y.; Li, Y.  米氮平治疗广泛性焦虑症32例疗效观察  2009;(1):206-208 | Language |
| Heimberg 1998 | Heimberg, R. G.; Liebowitz, M. R.; Hope, D. A.; Schneier, F. R.; Holt, C. S.; Welkowitz, L. A.; Juster, H. R.; Campeas, R.; Bruch, M. A.; Cloitre, M.; Fallon, B.; Klein, D. F. Cognitive behavioral group therapy vs phenelzine therapy for social phobia: 12-week outcome. Archives of General Psychiatry 1998;55(12):1133-1141 | Wrong outcomes, no data regarding critical outcomes within 4 weeks |
| Holland 1999 | Holland, R.; Musch, B.; Hindmarch, I. Specific effects of benzodiazepines and tricyclic antidepressants in panic disorder: comparisons of clomipramine with alprazolam SR and adinazolam SR. 1999;14(Journal Article):199-24 | Wrong outcomes, no data regarding critical outcomes within 4 weeks |
| Huang 2005 | Huang, T.; Zhu, J.; Jiang, X.; Zhou, D.; Zhang, F.  黄寅平,朱建中,蒋幸衍,周德怡,张峰.米氮平与阿普唑仑治疗广泛性焦虑的对照研究[J].中国行为医学科学  2005;10(Journal Article):919 | Language |
| Ichitovkina 2014 | Ichitovkina, E. G.; Zlokazova, M. V.; Solov’ev, A. G.  Efficacy of medical-psychological rehabilitation of combatants. 2014;44(Journal Article):933-938 | Wrong study design, observational study |
| Jia 2009 | Jia, Mingxian  贾裕堂,陈圣侠,朱凤玲.米氮平与帕罗西汀治疗广泛性焦虑障碍的对照研究[J].精神医学杂志  2009;22(03):209-210 | Language |
| Katschnig 1997 | Katschnig, k.; Stein, M. B.; Buller, R. Moclobemide in Social Phobia. Moclobemide in social phobia. A double-blind, placebo-controlled clinical study. European archives of psychiatry and clinical neuroscience 1997;247(2):71-80 | Wrong outcomes, no data regarding critical outcomes within 4 weeks |
| Khan 2011 | Khan, A.; Joyce, M.; Atkinson, S.; Eggens, I.; Baldytcheva, I.; Eriksson, H. A randomized, double-blind study of once-daily extended release quetiapine fumarate (quetiapine XR) monotherapy in patients with generalized anxiety disorder.Journal of clinical psychopharmacology 2011;31(4):418-428 | Wrong outcomes, no data regarding critical outcomes within 4 weeks |
| Khan 2016 | Khan, A; Durgam, S; Tang, X; Ruth, A; Mathews, M; Gommoll, C P. Post Hoc Analyses of Anxiety Measures in Adult Patients With Generalized Anxiety Disorder Treated With Vilazodone. The primary care companion for CNS disorders 2016;18(2) | Wrong intervention |
| Klerman 1986 | Klerman, G. L.; Coleman, J. H.; Purpura, R. P.  The design and conduct of the Upjohn Cross-National Collaborative Panic Study. Psychopharmacology bulletin 1986;22(1):59-64  United States 1986 | A protocol, Cross-National Collaborative Panic Study |
| Klosko 2016 | Klosko, J S.; Barlow, D H.; Tassinari, R; Cerny, J A.  A comparison of alprazolam and behavior therapy in treatment of panic disorder. The neurotic paradox: Progress in understanding and treating anxiety and related disorders., Vol.1 2016;(Journal Article):105-121 | Wrong outcomes, no data regarding critical outcomes within 4 weeks |
| Labbate 1994 | Labbate, L. A.; Pollack, M. H.; Otto, M. W.; Tesar, G. M.; Rosenbaum, J. F. The relationship of alprazolam and clonazepam dose to steady-state concentration in plasma. Journal of clinical psychopharmacology 1994;14(4):274-276 | Wrong outcomes, no data regarding critical outcomes within 4 weeks  Same study as Pollack 1993, Tesar 1987, Tesar 1991, Fava 1989 |
| **Lecrubier 1997a** | Lecrubier, Y.; Bakker, A.; Dunbar, G.; Judge, R. Collaborative Paroxetine Panic Study Investigators. A comparison of paroxetine, clomipramine and placebo in the treatment of panic disorder. Acta Psychiatrica Scandinavica 1997;95(2):145-152. | Wrong outcomes, no data regarding critical outcomes within 4 weeks  Same study as **Lecrubier 1997b, GSK 1993** |
| **Lecrubier 1997b** | Lecrubier, Y.; Judge, R. Collaborative Paroxetine Panic Study Investigators. Long-term evaluation of paroxetine, clomipramine and placebo in panic disorder.  Acta Psychiatrica Scandinavica 1997;95(2):153-160 | Wrong outcomes, no data regarding critical outcomes within 4 weeks  Same study as **Lecrubier 1997a, GSK 1993** |
| Leinonen 2000 | Leinonen, E.; Lepola, U.; Koponen, H.; Turtonen, J.; Wade, A.; Lehto, H. Citalopram controls phobic symptoms in patients with panic disorder: randomized controlled trial  Journal of psychiatry & neuroscience : JPN 2000;25(1):24-32 | Wrong outcomes, Wrong outcomes, no data regarding critical outcomes within 4 weeks  Same study as Wade 1997, Wade 1999, Lepola 1998 |
| Lepola 1998 | Lepola, U. M.; Wade, A. G.; Leinonen, E. V.; Koponen, H. J.; Frazer, J.; Sjödin, I.; Penttinen, J. T.; Pedersen, T.; Lehto, H. J. A controlled, prospective, 1-year trial of citalopram in the treatment of panic disorder. The Journal of clinical psychiatry 1998;59(10):528-534 | Wrong outcomes, Wrong outcomes, no data regarding critical outcomes within 4 weeks  Same study as Wade 1997, Wade 1999, Leinonen 2000 |
| Li 2005 | Li  李卫军,刘晓红.米氮平治疗广泛性焦虑症对照研究[J]. 神经疾病与精神卫生  2005;(06):447-448 | Language |
| Li 2011 | Li  李刚.艾司西酞普兰与氟西汀治疗广泛性焦虑的对照研究[J].现代医药卫生  2011;27(09):1287-1289 | Language |
| Liu 2004 | Liu   刘祥臣,陈悦霞,苏荣红.氟西汀与地西泮治疗广泛性焦虑症的对照研究[J].中国行为医学科学  2004;(03):43-44 | Language |
| Liux 2005 | Liux  刘晓伟,杨雀萍,曹磊明,王进良.米氮平和丁螺环酮治疗广泛性焦虑症的对照研究[J].中国民康医学  2005;(09):495-496 | Language |
| Lott 1997 | Lott, M.; Greist, J. H.; Jefferson, J. W.; Kobak, K. A.; Katzelnick, D. J.; Katz, R. J.; Schaettle, S. C.. Brofaromine for social phobia: a multicenter, placebo-controlled, double-blind study. Journal of clinical psychopharmacology 1997;17(4):255-260 | Wrong outcomes, no data regarding critical outcomes within 4 weeks |
| Meco 1989 | Meco, G.; Capriani, C.; Bonifati, U. Etizolam: a new therapeutic possibility in the treatment of panic disorder. Advances in Therapy 1989;6(4):196-206. 1989;6(4):196-206 | Wrong comparator |
| Marcourakis 1993 | Marcourakis, T.; Gorenstein, C.; Gentil, V. Clomipramine, a better reference drug for panic/agoraphobia. II. Psychomotor and cognitive effects. Journal of psychopharmacology (Oxford, England) 1993;7(4):325-330 | Wrong outcomes, no data regarding critical outcomes within 4 weeks  Same study as Gentil1993 |
| Mirzaei 2021 | Mirzaei, E; Mirjalili, M; Jahangard, Leila; H, Mohammad; Yasrebifar, F; Mohammadi, Y; Larki-Harchagani, A; Mehrpooya, M. Influence of Simvastatin as Augmentative Therapy in the Treatment of Generalized Anxiety Disorder: A Pilot Randomized, Placebo-Controlled Study. Neuropsychobiology 2021;80(3):242-252 | Wrong intervention |
| Muehlbacher 2005 | Muehlbacher, M.; Nickel, M. K.; Nickel, C.; Kettler, C.; Lahmann, C.; Pedrosa Gil, F.; Leiberich, P. K.; Rother, N.; Bachler, E.; Fartacek, R.; Kaplan, P.; Tritt, K.; Mitterlehner, F.; Anvar, J.; Rother, W. K.; Loew, T. H.; Egger, C. Mirtazapine treatment of social phobia in women: a randomized, double-blind, placebo-controlled study. Journal of clinical psychopharmacology 2005;25(6):580-583 | Wrong outcomes, no data regarding critical outcomes within 4 weeks |
| **Nair 1996** | Comparison of fluvoxamine, imipramine, and placebo in the treatment of outpatients with panic disorder  Nair, N. P.; Bakish, D.; Saxena, B.; Amin, M.; Schwartz, G.; West, T. E.  Anxiety 1996;2(4):192-198  United States 1996 | Wrong outcomes, no data regarding critical outcomes within 4 weeks  Same study as Bakish 1996 |
| Niu 2004 | Niu  钮富荣,沈鑫华,孙松涛.米氮平与氟西汀治疗伴有广泛焦虑障碍的抑郁症各35例的疗效比较[J].中国新药与临床杂志  2004;(12):853-855 | Language |
| Noyes 1997 | Noyes, R.,Jr; Moroz, G.; Davidson, J. R.; Liebowitz, M. R.; Davidson, A.; Siegel, J.; Bell, J.; Cain, J. W.; Curlik, S. M.; Kent, T. A.; Lydiard, R. B.; Mallinger, A. G.; Pollack, M. H.; Rapaport, M.; Rasmussen, S. A.; Hedges, D.; Schweizer, E.; Uhlenhuth, E. H. Moclobemide in social phobia: a controlled dose-response trial. Journal of clinical psychopharmacology 1997;17(4):247-254 | Wrong outcomes, no data regarding critical outcomes within 4 weeks |
| Oliveira 2022 | Oliveira, P S C; Sant'Anna, B C; Seixas, N B; Mendonca, J A. Low-dose midazolam for anxiolysis for pregnant women undergoing cesarean delivery: a randomized trial. Brazilian journal of anesthesiology (Elsevier) 2022;72(4):450-456 | Wrong patient population |
| Oosterbaan 2001 | Oosterbaan, D. B.; Van Balkom, A. J. L. M.; Spinhoven, P.; van Oppen, P.; van Dyck, R. Cognitive therapy versus moclobemide in social phobia: a controlled study. 2001;8(4):263-273 | Wrong outcomes, no data regarding critical outcomes within 4 weeks |
| Pollack 1993 | Pollack, M. H.; Otto, M. W.; Tesar, G. E.; Cohen, L. S.; Meltzer-Brody, S.; Rosenbaum, J. F. Long-term outcome after acute treatment with alprazolam or clonazepam for panic disorder. Journal of clinical psychopharmacology 1993;13(4):257-263 | Wrong outcomes, no data regarding critical outcomes within 4 weeks  Same study as Labbate 1994, Tesar 1987, Tesar 199, Fava 1989 |
| Pfizer 2012 | Safety and Efficacy Evaluation Of Pregabalin (Lyrica) With Patients With Generalized Anxiety Disorder Long Term Safety And Efficacy Study Of Pregabalin (Lyrica) In Subjects With Generalized Anxiety Disorder NCT 00624780 (Public Disclosure Synopsis for protocol A0081147) | A protocol |
| Peng 2012 | Peng  彭岚,吴东,涂军,廖波,冷小兵,江昆伙.氟西汀与阿普唑仑治疗广泛性焦虑的对照研究[J].中国现代医生  2012;50(31):78-79 | Language |
| Ravindran 2022 | Ravindran, N; McKay, M; Paric, A; Johnson, S; Chandrasena, R; Abraham, G; Ravindran, A V. Randomized, Placebo-Controlled Effectiveness Study of Quetiapine XR in Comorbid Depressive and Anxiety Disorders. The Journal of clinical psychiatry 2022;83(3) | Wrong outcomes, no data regarding critical outcomes within 4 weeks |
| Ribeiro 2001 | Ribeiro, L.; Busnello, J. V.; Kauer-Sant'Anna, M.; Madruga, M.; Quevedo, J.; Busnello, E. A.; Kapczinski, F. Mirtazapine versus fluoxetine in the treatment of panic disorder. Brazilian journal of medical and biological research = Revista brasileira de pesquisas medicas e biologicas 2001;34(10):1303-1307 | Wrong outcomes, no data regarding critical outcomes within 4 weeks |
| Rothschild 2012 | Rothschild, A. J.; Mahableshwarkar, A. R.; Jacobsen, P.; Yan, M.; Sheehan, D. V.  Vortioxetine (Lu AA21004) 5 mg in generalized anxiety disorder: results of an 8-week randomized, double-blind, placebo-controlled clinical trial in the United States. European neuropsychopharmacology : the journal of the European College of Neuropsychopharmacology 2012;22(12):858-866 | Wrong intervention |
| Sasson 1999 | Sasson, Y.; Iancu, I.; Fux, M.; Taub, M.; Dannon, P. N.; Zohar, J. A double-blind crossover comparison of clomipramine and desipramine in the treatment of panic disorder. European neuropsychopharmacology : the journal of the European College of Neuropsychopharmacology 1999;9(3):191-196 | Wrong outcomes, no data regarding critical outcomes within 4 weeks |
| Schneider 2020 | Schneider, R B.; Auinger, P; Tarolli, C G.; Iourinets, J; Gil-Diaz, M; Richard, I H. A trial of buspirone for anxiety in Parkinson's disease: Safety and tolerability. Parkinsonism & related disorders 2020;81(Journal Article):69-74 | Wrong outcomes, no data regarding critical outcomes within 4 weeks |
| Schneier 1998 | Schneier, F. R.; Goetz, D.; Campeas, R.; Fallon, B.; Marshall, R.; Liebowitz, M. R. Placebo-controlled trial of moclobemide in social phobia. The British journal of psychiatry : the journal of mental science 1998;172(Journal Article):70-77 | Wrong outcomes, no data regarding critical outcomes within 4 weeks |
| Schutters 2010 | Schutters, S. I.; Van Megen, H. J.; Van Veen, J. F.; Denys, D. A.; Westenberg, H. G. Mirtazapine in generalized social anxiety disorder: a randomized, double-blind, placebo-controlled study. International clinical psychopharmacologicology 2010; 25 (5): 302-304 | Wrong outcomes, no data regarding critical outcomes within 4 weeks |
| Sedighi 2020 | Sedighi S.; Rahmani S.; Moinolghorabaei M. The effects of analytic group therapy compared with pharmacotherapy in patients with anxiety disorders. Biomedical Research and Therapy 2020;7(12):4152-4157 | Wrong intervention and wrong comparator |
| Servant 1998 | Servant, D.; Graziani, P. L.; Moyse, D.; Parquet, P. J.  Treatment of adjustment disorder with anxiety: efficacy and tolerance of etifoxine in a double-blind controlled study  L'Encephale 1998;24(6):569-574  France 1998 | Language |
| Shahrokhi 2021 | Shahrokhi, M; Ghaeli, P; Arya, P; Shakiba, A; Noormandi, A; Soleimani, M; Esfandbod, M. Comparing the Effects of Melatonin and Zolpidem on Sleep Quality, Depression, and Anxiety in Patients With Colorectal Cancer Undergoing Chemotherapy. Basic and clinical neuroscience 2021;12(1):105-114 | Wrong patient population |
| Sheikh 1999 | Sheikh, J. I.; Swales, P. J. Treatment of panic disorder in older adults: a pilot study comparison of alprazolam, imipramine, and placebo. nternational journal of psychiatry in medicine 1999;29(1):107-117 | Wrong outcomes, no data regarding critical outcomes within 4 weeks |
| Slaap 1995 | Slaap, B. R.; van Vliet, I. M.; Westenberg, H. G.; den Boer, J. A. Phobic symptoms as predictors of nonresponse to drug therapy in panic disorder patients (a preliminary report). Journal of affective disorders 1995;33(1):31-38 | Wrong outcomes, no data regarding critical outcomes within 4 weeks |
| Song 2007 | Song  宋传福,陶忠,武慎彬.米氮平治疗广泛性焦虑对照研究 [J].临床精神医学杂志  2007;(05):323-324 | Language |
| Stein 2002 | Stein, D. J.; Cameron, A.; Amrein, R.; Montgomery, S. A.; Moclobemide Social Phobia Clinical Study Group. Moclobemide is effective and well tolerated in the long-term pharmacotherapy of social anxiety disorder with or without comorbid anxiety disorder. nternational clinical psychopharmacology 2002;17(4):161-170 | Wrong outcomes, no data regarding critical outcomes within 4 weeks |
| Stein 2018 | Stein, D J.; Khoo, J P; Ahokas, A; Jarema, M; Van Ameringen, M; Vavrusova, L; Hoschl, C; Bauer, M; Bitter, I; Mosolov, S N.; Olivier, V; Matharan, S; Picarel-Blanchot, F; de Bodinat, C.12-week double-blind randomized multicenter study of efficacy and safety of agomelatine (25-50mg/day) versus escitalopram (10-20mg/day) in out-patients with severe generalized anxiety disorder. European neuropsychopharmacology : the journal of the European College of Neuropsychopharmacology 2018;28(8):970-979 | Wrong outcomes, no data regarding critical outcomes within 4 weeks |
| Syunyakov 2016 | Syunyakov, T. S.; Neznamov, G. G. Evaluation of the therapeutic efficacy and safety of the selective anxiolytic afobazole in generalized anxiety disorder and adjustment disorders: Results of a multicenter randomized comparative study of diazepam  Terapevticheskii arkhiv 2016;88(8):73-86  Russia (Federation) 2016 | Language |
| Tesar 1987 | Tesar, G. E.; Rosenbaum, J. F.; Pollack, M. H.; Herman, J. B.; Sachs, G. S.; Mahoney, E. M.; Cohen, L. S.; McNamara, M.; Goldstein, S.  Clonazepam versus alprazolam in the treatment of panic disorder: interim analysis of data from a prospective, double-blind, placebo-controlled trial. The Journal of clinical psychiatry 1987;48 Suppl(Journal Article):16-21 | Wrong outcomes, no data regarding critical outcomes within 4 weeks  Same study as Labbate 1994, Pollack 1993, Tesar 1991, Fava 1989 |
| Tesar 1991 | Tesar, G. E.; Rosenbaum, J. F.; Pollack, M. H.; Otto, M. W.; Sachs, G. S.; Herman, J. B.; Cohen, L. S.; Spier, S. A. Double-blind, placebo-controlled comparison of clonazepam and alprazolam for panic disorder. The Journal of clinical psychiatry 1991;52(2):69-76 | Wrong outcomes, no data regarding critical outcomes within 4 weeks  Same study as Labbate 1994, Pollack 1993, Tesar 1987, Fava 1989 |
| Uhlenhuth 1989 | Uhlenhuth, E. H.; Matuzas, W.; Glass, R. M.; Easton, C. Response of panic disorder to fixed doses of alprazolam or imipramine. Journal of affective disorders 1989;17(3):261-270 | Wrong outcomes, no data regarding critical outcomes within 4 weeks |
| Vaishnavi 2007 | Vaishnavi, S.; Alamy, S.; Zhang, W.; Connor, K. M.; Davidson, J. R. Quetiapine as monotherapy for social anxiety disorder: a placebo-controlled study. Progress in neuro-psychopharmacology & biological psychiatry 2007;31(7):1464-1469 | Wrong outcomes, no data regarding critical outcomes within 4 weeks |
| Van Ameringen 2007 | Van Ameringen, M.; Mancini, C.; Oakman, J.; Walker, J.; Kjernisted, K.; Chokka, P.; Johnston, D.; Bennett, M.; Patterson, B.  Nefazodone in the treatment of generalized social phobia: a randomized, placebo-controlled trial. The Journal of clinical psychiatry 2007;68(2):288-295 | Wrong intervention |
| van Vliet 1996 | van Vliet, I. M.; den Boer, J. A.; Westenberg, H. G.; Slaap, B. R. A double-blind comparative study of brofaromine and fluvoxamine in outpatients with panic disorder. Journal of clinical psychopharmacology 1996;16(4):299-306 | Wrong outcomes, no data regarding critical outcomes within 4 weeks |
| Vicente 2020 | Vicente, B; Saldivia, S; Hormazabal, N; Bustos, C; Rubi, P. Etifoxine is non-inferior than clonazepam for reduction of anxiety symptoms in the treatment of anxiety disorders: a randomized, double blind, non-inferiority trial. Psychopharmacology 2020;237(11):3357-3367 | Wrong outcomes, Wrong outcomes, no data regarding critical outcomes within 4 weeks |
| Wade 1997 | Wade, A. G.; Lepola, U.; Koponen, H. J.; Pedersen, V.; Pedersen, T. The effect of citalopram in panic disorder. The British journal of psychiatry : the journal of mental science 1997;170(Journal Article):549-553 | Wrong outcomes, Wrong outcomes, no data regarding critical outcomes within 4 weeks  Same study as Wade 1999, Leinonen 2000, Lepola 1998 |
| Wade 1999 | Wade A,; Overe, K. F.; Lemming, O. Weight monitoring during two long-term trials of citalopram. 1999;9(Suppl 5):S221 | Wrong outcomes, Wrong outcomes, no data regarding critical outcomes within 4 weeks  Same study as Wade 1997, Wade 1997, Leinonen 2000, Lepola 1998 |
| Wang 2009 | Wang  王树元,李丛梅.米氮平与帕罗西汀治疗广泛性焦虑的对照研究[J].临床精神医学杂志  2009;19(02):76 | Language |
| Wang 2015 | Wang, Limin; Zhong, Zhuoyuan; Hu, Jingyang; Rong, Xiaoming; Liu, Jun; Xiao, Songhua; Liu, Zhonglin. Sertraline plus deanxit to treat patients with depression and anxiety in chronic somatic diseases: A randomized controlled trial. BMC Psychiatry 2015;15: 84 | Wrong intervention |
| Westenberg 1989 | Westenberg, H. G.; den Boer, J. A. Selective monoamine uptake inhibitors and a serotonin antagonist in the treatment of panic disorder. Psychopharmacology bulletin 1989;25(1):119-123 | Wrong outcomes, no usable data |
| Wolitzky-Taylor 2018 | Wolitzky-Taylor, K; Niles, A N.; Ries, R; Krull, J L.; Rawson, R; Roy-Byrne, Peter; Craske, Mi.  Who needs more than standard care? Treatment moderators in a randomized clinical trial comparing addiction treatment alone to addiction treatment plus anxiety disorder treatment for comorbid anxiety and substance use disorders. | Wrong intervention |
| Yang 2005 | Yang  杨福收,王新法,王新友.米氮平治疗广泛性焦虑症的疗效及安全性[J].中国新药杂志  2005;(05):113-115 | Language |
| Yong 2017 | Young A.; Patrick F.; Wise T.; Meyer N.; Mazibuko N.; Oates A.E.; Van Der Bijl A.M.H.; Danjou P.; O'Connor S.; Doolin E.; Wooldridge C.; MacAre C.; Williams S.C.R.; Perkins A.; Young A.H.. Modulation of anxiety-relevant neural circuits in generalized anxiety disorder: A novel cholinergic system pharmacotherapy approach. Biological psychiatry 2017;81(10):S70 | Wrong intervention |
| Zammit 2019 | Zammit G.; Mayleben D.; Kumar D.; Moline M. Efficacy of lemborexant vs zolpidem extended release and placebo in elderly subjects with insomnia: Results from sunrise 1. Journal of the American Geriatrics Society 2019;67(Journal Article):S51-S52 | Wrong patient population |
| Zammit 2020 | Zammit G.; Mayleben D.; Kumar D.; Moline M.  Efficacy and safety of lemborexant vs zolpidem extended release and placebo in elderly subjects with insomnia: Results from sunrise-1. Neurology 2020;94(15) | Wrong patient population |
| Zullino 2015 | Zullino D.; Chatton A.; Fresard E.; Stankovic M.; Bondolfi G.; Borgeat F.; Khazaal Y.  Venlafaxine Versus Applied Relaxation for Generalized Anxiety Disorder: A Randomized Controlled Study on Clinical and Electrophysiological Outcomes. Psychiatric Quarterly 2015;86(1):69-82 | Wrong intervention |

# Supplementary Table S3. AMSTAR assessment of included systematic reviews.

| Citation | Outcomes | Was meta-analysis performed for the relevant outcomes? | 1. Was an 'a priori' design provided? | 2. Was there duplicate study selection and data extraction? | 3. Was a comprehensive literature search performed? | 4. Was the status of publication (i.e. grey literature) used as an inclusion criterion? | 5. Was a list of studies (included and excluded) provided? | 6. Were the characteristics of the included studies provided? | 7. Was the scientific quality of the included studies assessed and documented? | 8. Was the scientific quality of the included studies used appropriately in formulating conclusions? | 9. Were the methods used to combine the findings of studies appropriate? | 10. Was the likelihood of publication bias assessed? | 11. Was the conflict of interest included? |
| --- | --- | --- | --- | --- | --- | --- | --- | --- | --- | --- | --- | --- | --- |
| O'Donnell et al 2018 | Changes in anxiety symptoms | No | No | Yes | Yes | No | No | Yes | Yes | Yes | Not applicable | Not applicable | No |
| Slee et al. 2019 | Changes in anxiety symptoms | Yes | Yes | No | No | Yes | No | Yes | Yes | No | Yes | No | No |
| Williams et al. 2020 | Changes in anxiety symptoms | Yes | Yes | Yes | Yes | yes | No | Yes | Yes | No | Yes | No | No |
| Bighelli et al. 2016 | Anxiety symptoms | Yes | Yes | Yes | Yes | yes | Yes | Yes | Yes | Yes | Yes | Yes | No |

# Supplementary Figure S2. Risk of bias assessment.


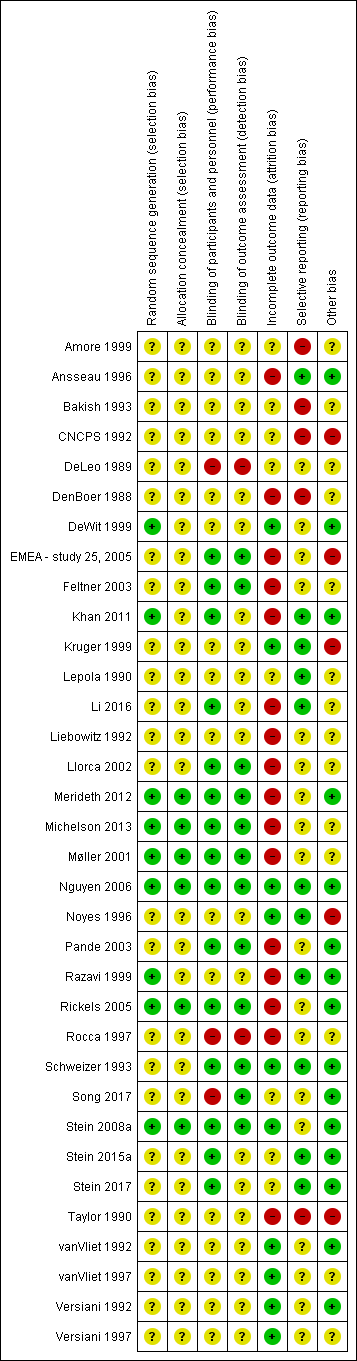


##

## Supplementary Table S3. CINeMA assessment.

|  |  |  | **CINeMA domains** | | | | | |  |
| --- | --- | --- | --- | --- | --- | --- | --- | --- | --- |
|  |  |  | **1** | **2** | **3** | **4** | **5** | **6** |  |
| **Comparison** | **No. direct studies** | **Network estimate** | **Within-study bias** | **Reporting bias** | **Indirectness** | **Imprecision** | **Heterogeneity** | **Incoherence** | **Confidence rating** |
| Agomelatin:Placebo | 2 | -0.22 (-0.67; 0.24) | Some concerns | Low risk | Major concerns | Some concerns | Some concerns | No concerns | **Very low^1,3,4,5^** |
| Benzodiazepin:Placebo | 13 | -0.58 (-0.77; -0.40) | Major concerns | Low risk | Major concerns | No concerns | Some concerns | No concerns | **Low^1,3^** |
| Hydroxyzine:Placebo | 1 | -0.30 (-0.87; 0.26) | Major concerns | Low risk | Major concerns | Some concerns | Some concerns | No concerns | **Very low^1,3,4,5^** |
| Mianserin:Placebo | 0 | -0.45 (-1.17; 0.27) | Major concerns | Low risk | Major concerns | Some concerns | Some concerns | No concerns | **Very low^1,3,4,5^** |
| Pregabalin:Placebo | 4 | -0.58 (-0.87; -0.28) | Major concerns | Low risk | Major concerns | No concerns | Some concerns | No concerns | **Low^1,3^** |
| Quetiapin:Placebo | 3 | -0.51 (-0.90; -0.13) | Major concerns | Low risk | Major concerns | No concerns | Some concerns | No concerns | **Low^1,3^** |
| Agomelatin:Benzodiazepin | 0 | 0.37 (-0.12; 0.86) | Major concerns | Low risk | Major concerns | Some concerns | Some concerns | No concerns | **Very low^1,3,4,5^** |
| Agomelatin:Hydroxyzine | 0 | 0.09 (-0.64; 0.81) | Major concerns | Low risk | Major concerns | Major concerns | No concerns | No concerns | **Very low^1,3,4^** |
| Agomelatin:Mianserin | 0 | 0.23 (-0.62; 1.09) | Major concerns | Low risk | Major concerns | Major concerns | No concerns | No concerns | **Very low^1,3,4^** |
| Agomelatin:Pregabalin | 0 | 0.36 (-0.18; 0.90) | Major concerns | Low risk | Major concerns | Some concerns | Some concerns | No concerns | **Very low^1,3,4,5^** |
| Agomelatin:Quetiapin | 0 | 0.30 (-0.30; 0.90) | Major concerns | Low risk | Major concerns | Some concerns | Some concerns | No concerns | **Very low^1,3,4,5^** |
| Benzodiazepin:Hydroxyzine | 1 | -0.28 (-0.85; 0.28) | Major concerns | Low risk | Major concerns | Some concerns | Some concerns | No concerns | **Very low^1,3,4,5^** |
| Benzodiazepin:Mianserin | 1 | -0.14 (-0.84; 0.56) | Major concerns | Low risk | Some concerns | Major concerns | No concerns | No concerns | **Very low^1,3,4^** |
| Benzodiazepin:Pregabalin | 4 | -0.01 (-0.30; 0.28) | Major concerns | Low risk | Major concerns | No concerns | Major concerns | No concerns | **Low^1,3^** |
| Benzodiazepin:Quetiapin | 0 | -0.07 (-0.49; 0.35) | Major concerns | Low risk | Major concerns | Major concerns | No concerns | No concerns | **Very low^1,3,4^** |
| Hydroxyzine:Mianserin | 0 | 0.15 (-0.75; 1.04) | Major concerns | Low risk | Major concerns | Major concerns | No concerns | No concerns | **Very low^1,3,4^** |
| Hydroxyzine:Pregabalin | 0 | 0.28 (-0.35; 0.90) | Major concerns | Low risk | Major concerns | Major concerns | No concerns | No concerns | **Very low^1,3,4^** |
| Hydroxyzine:Quetiapin | 0 | 0.21 (-0.47; 0.89) | Major concerns | Low risk | Major concerns | Major concerns | No concerns | No concerns | **Very low^1,3,4^** |
| Mianserin:Pregabalin | 0 | 0.13 (-0.63; 0.89) | Major concerns | Low risk | Major concerns | Major concerns | No concerns | No concerns | **Very low^1,3,4^** |
| Mianserin:Quetiapin | 0 | 0.06 (-0.75; 0.88) | Major concerns | Low risk | Major concerns | Major concerns | No concerns | No concerns | **Very low^1,3,4^** |
| Pregabalin:Quetiapin | 0 | -0.06 (-0.54; 0.42) | Major concerns | Low risk | Major concerns | Major concerns | No concerns | No concerns | **Very low^1,3,4^** |

Within-study bias was evaluated based on average RoB for each comparison. Reporting bias was evaluated based on inspection of funnel plots from direct comparisons when 10 or more trials were available. Indirectness was evaluated based on average indirectness for each comparison. Imprecision, heterogeneity, and incoherence were evaluated considering a clinically important effect size of 0.3 SMD. 1: Within-study bias, 2: Reporting bias, 3: Indirectness (due to differences between the population of interest and those studied.), 4: Imprecision, 5: Heterogeneity, 6: Incoherence.

# Supplementary Figure S3. Forest plot for the effect of each treatment against placebo on HAM-A (SMDs with 95% CrI) – Bayesian (sensitivity).


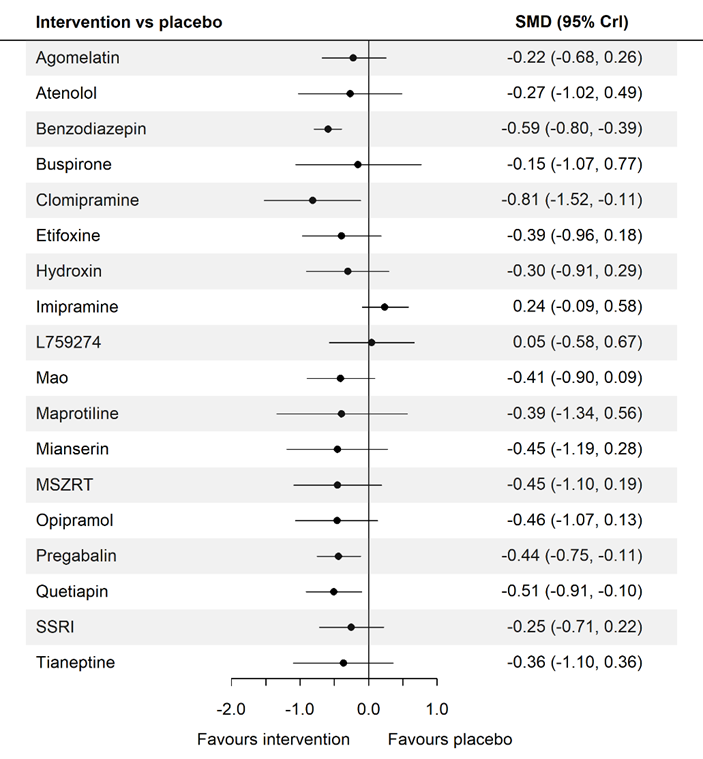


CrI=credible interval

**Supplementary Figure S4**. Ranking of treatments.

*A) Based on analysis with SMDs*


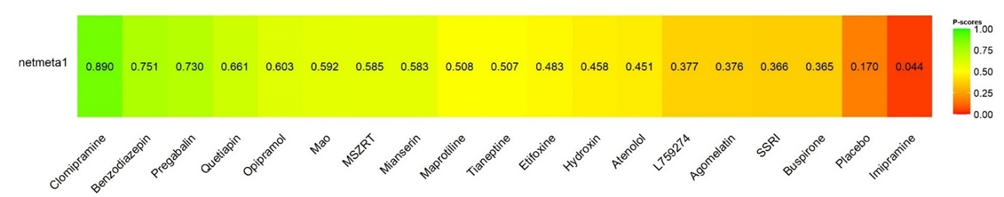


*B) Based on analysis with MDs*


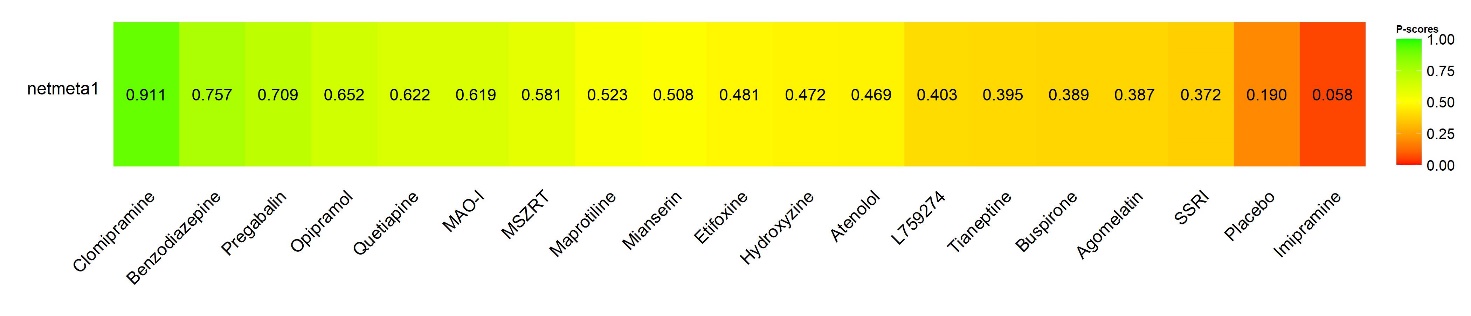


Ranking of the individual treatments based on p-scores indicated with colours. P-scores describe the extent of certainty that a treatment is better than the other competing treatments.

# Supplementary Figure S5. Forest plot for the effect of each treatment against placebo on HAM-A (MDs with 95%CIs).


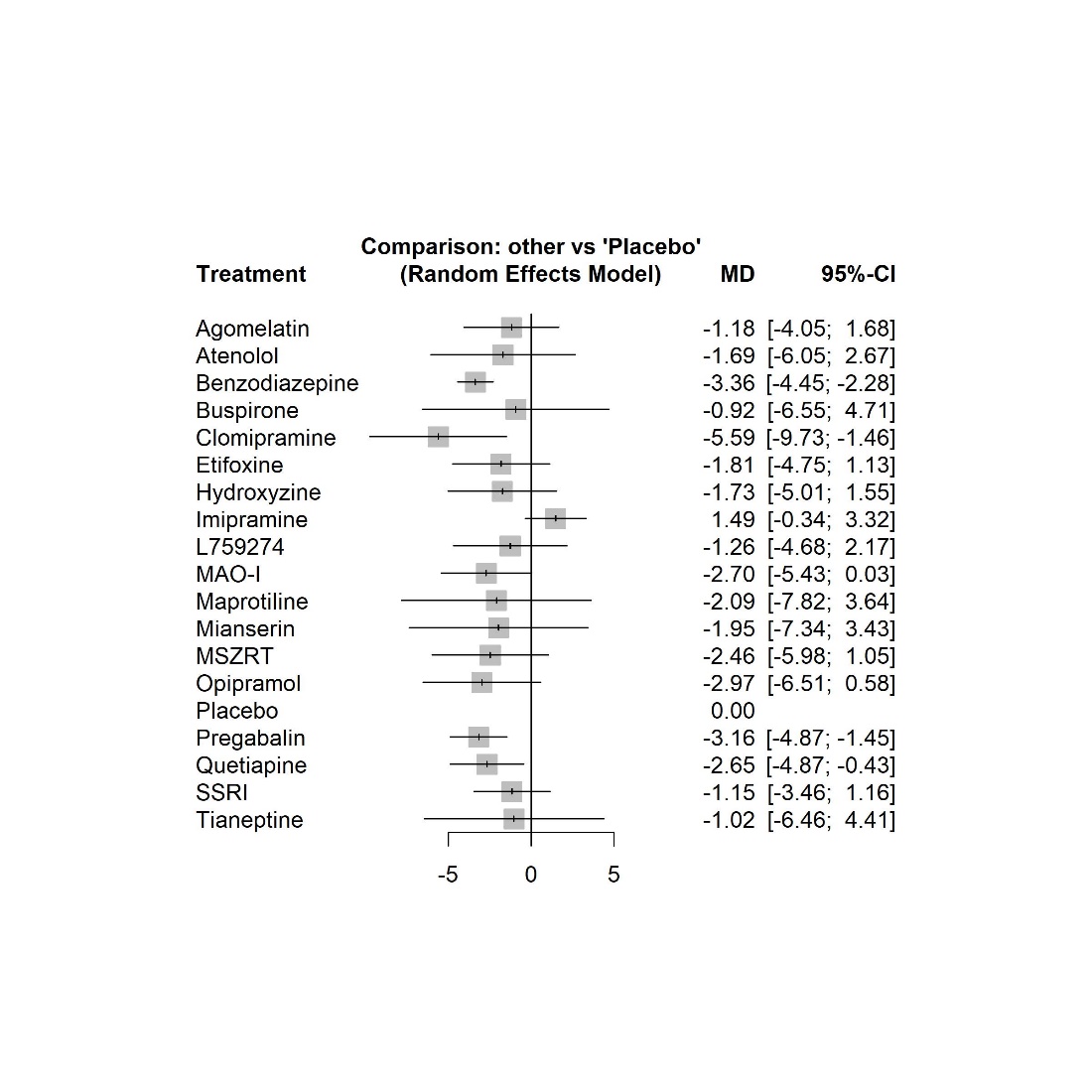


# Supplementary Figure S6. Consistency assessed with node splitting.

There was no evidence of inconsistency (i.e., the agreement between direct and indirect evidence), when considering direct and indirect evidence separately with node-splitting. Below are the results for the effects of different comparisons when using only direct, only indirect and all available evidence (ideally these should not differ substantially).


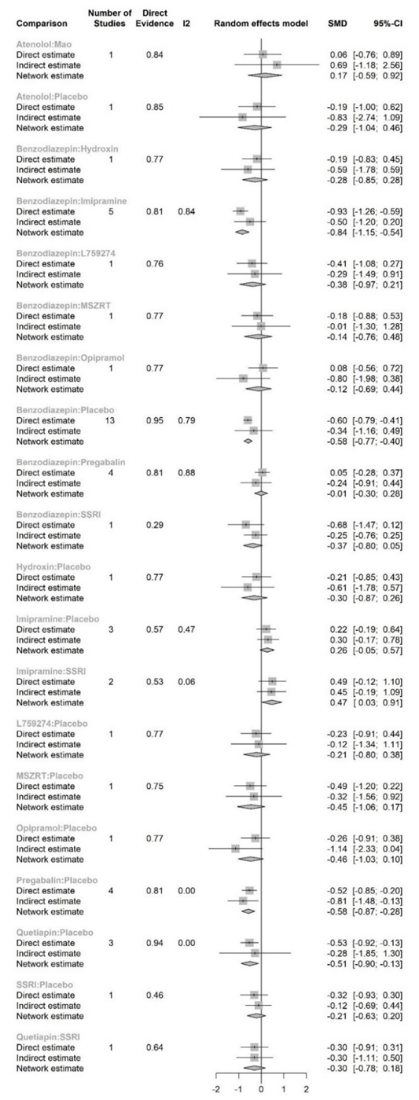


# Supplementary Figure S7. Transitivity.

The transitivity assumption underlying the network meta-analyses (i.e., that the network includes studies that are sufficiently similar in important clinical and methodological characteristics).

*A) Diagnosis*


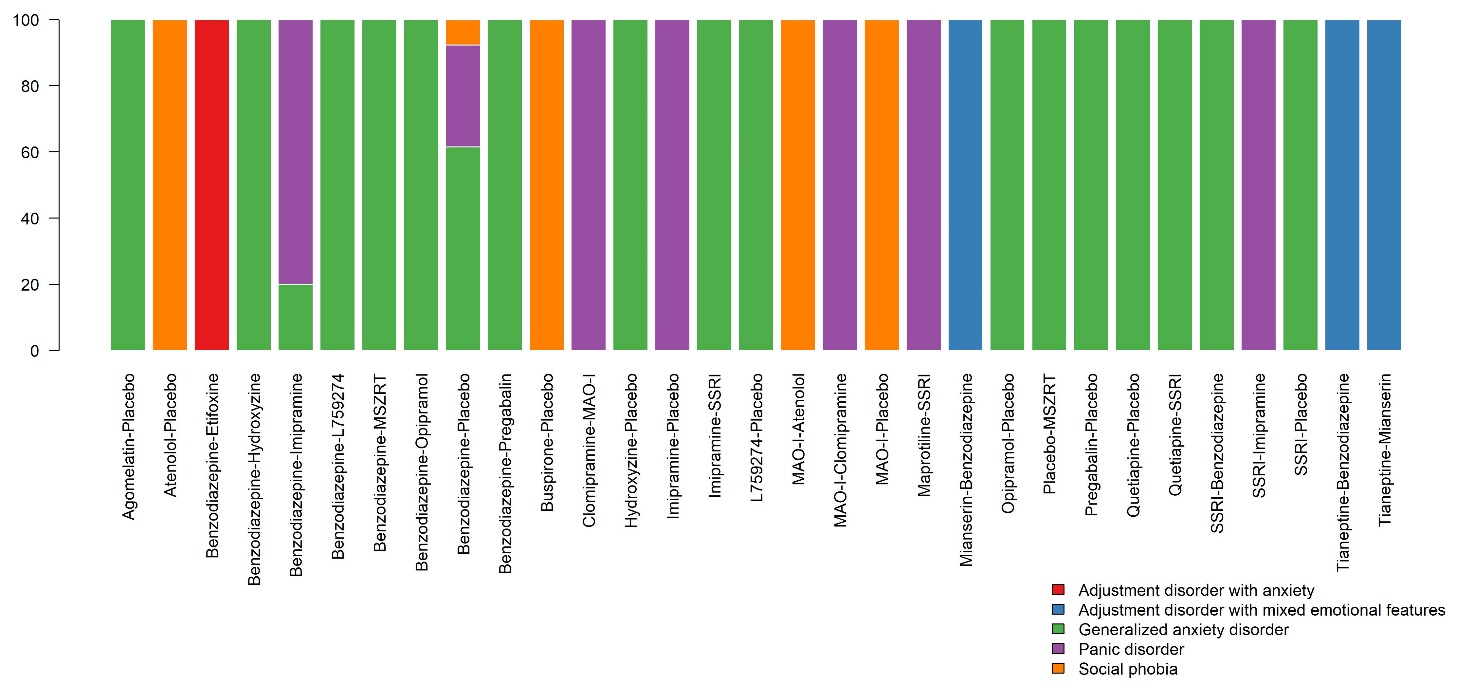


*B) Co-medication*


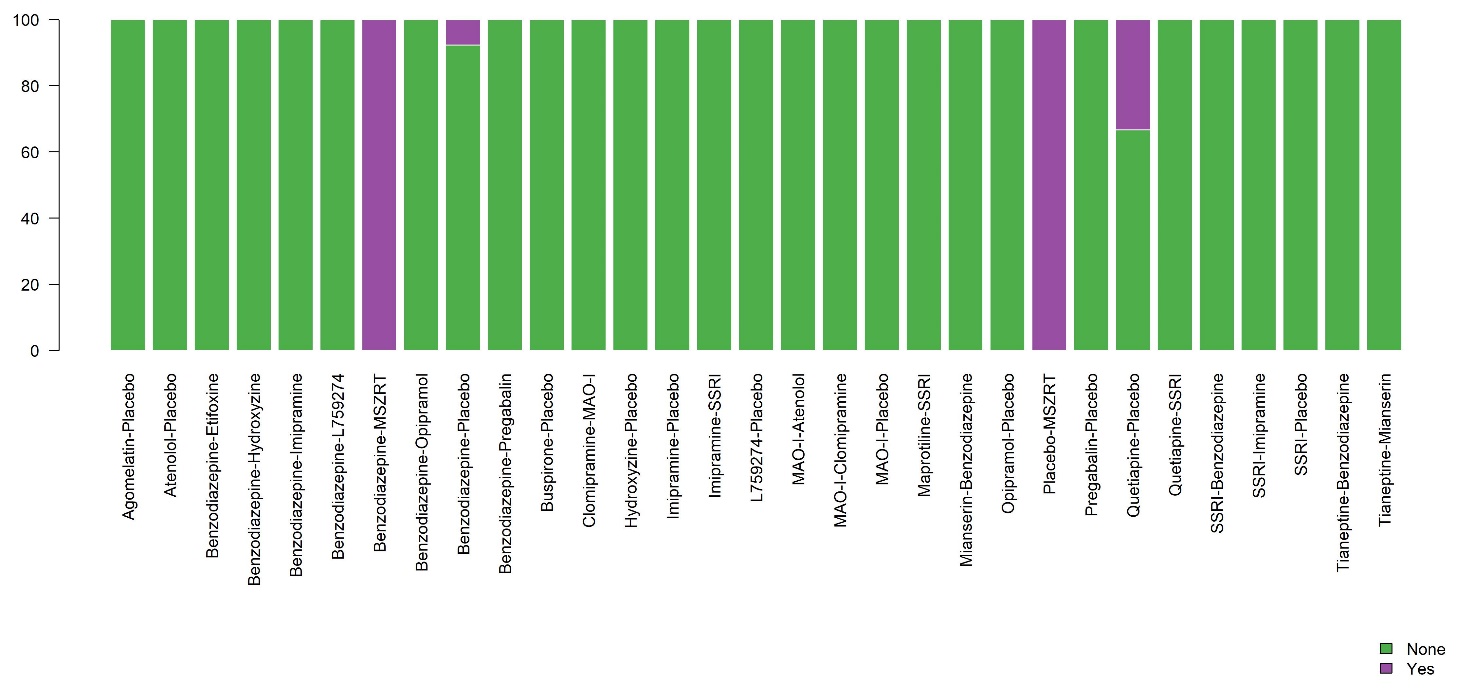


*C) Placebo run-in*


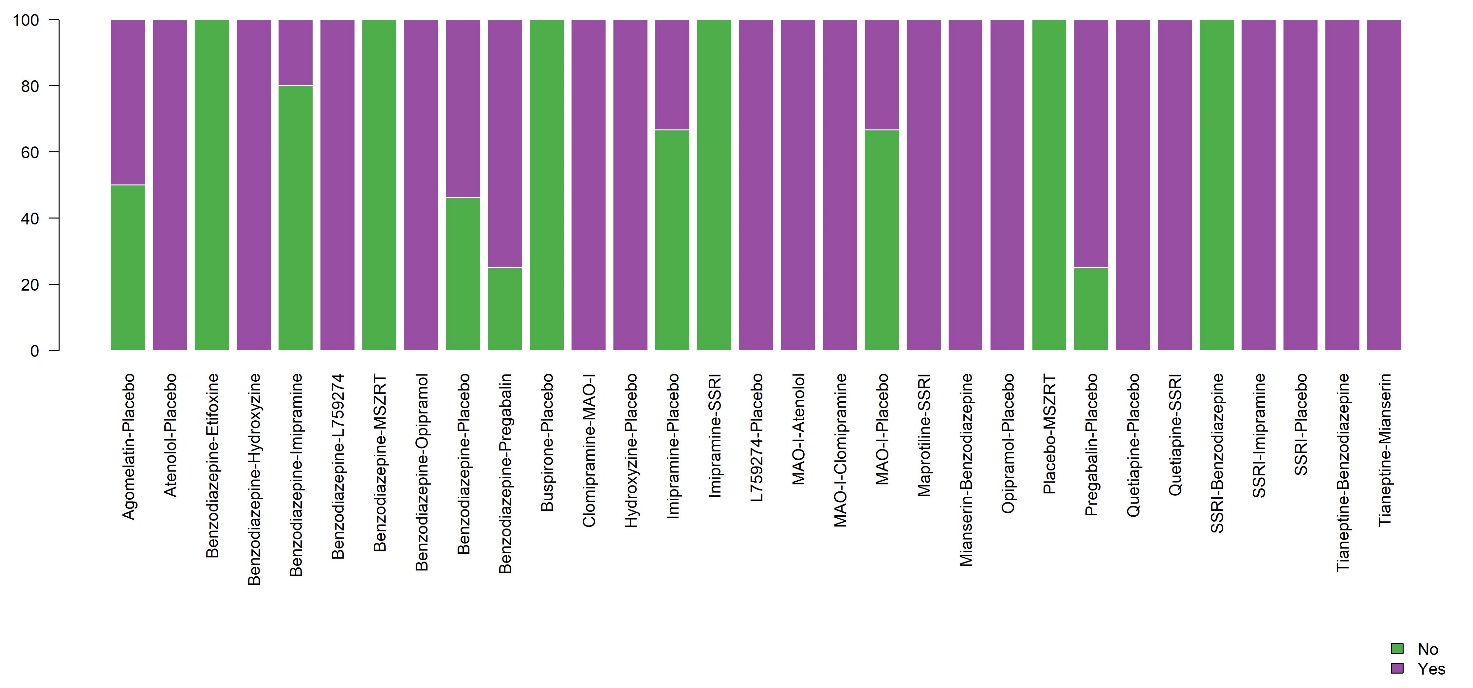


# Supplementary Table S4. SUCRA (based on SMD) – Bayesian (sensitivity).

| **Treatment** | **SUCRA** |
| --- | --- |
| Clomipramine | 0.858 |
| Benzodiazepines | 0.778 |
| Quetiapine | 0.674 |
| Opipramol | 0.615 |
| MSZRT | 0.606 |
| Mianserin | 0.604 |
| Pregabalin | 0.603 |
| MAO-I | 0.569 |
| Etifoxine | 0.555 |
| Maprotiline | 0.548 |
| Tianeptine | 0.528 |
| Hydroxyzine | 0.473 |
| Atenolol | 0.449 |
| SSRI | 0.418 |
| Agomelatin | 0.396 |
| Buspirone | 0.377 |
| L759274 | 0.205 |
| Placebo | 0.184 |
| Imipramine | 0.061 |

The surface under the cumulative ranking curve (SUCRA) is a numeric presentation of the overall ranking and presents a single number associated with each treatment. SUCRA values range from 0 to 1. The higher the SUCRA value, the higher the likelihood that a therapy is in the top rank or one of the top ranks; the closer to 0, the more likely that a therapy is in the bottom rank, or one of the bottom ranks.

# Supplementary results

## Pair-wise meta-analyses of critical outcomes

### Pair-wise meta-analysis of short-term treatment with benzodiazepines compared with placebo

*Symptoms of anxiety*

Short-term treatment with benzodiazepine may reduce symptoms of anxiety (SMD 0.6, 95% CI: 0.79 to 0.41, 13 studies, low certainty evidence).

*Serious adverse events*

The evidence is very uncertain about the effect of short-term treatment with benzodiazepine on number of patients with serious adverse events (RR 1.43, 95% CI: 0.43 to 4.80, 9 studies, very low certainty evidence).

*Function of daily living*

One study reported data for the critical outcome of function of daily living and the evidence is very uncertain about the effect of short-term treatment with benzodiazepine on function for all dimensions: function (SMD 0.57, 95% CI: 0.05 to 1.08, very low certainty evidence), social life (SMD 0.59, 95% CI: 0.07 to 1.10, very low certainty evidence), and family life (SMD 0.74, 95% CI: 0.22 to 1.27, very low certainty evidence).

### Pair-wise meta-analysis of short-term treatment with pregabalin compared with placebo

*Symptoms of anxiety*

Short-term treatment with pregabalin may reduce symptoms of anxiety (SMD 0.53, 95% CI: 0.38 to 0.68, 4 studies, low certainty evidence).

*Serious adverse events*

The evidence is very uncertain about the effect of short-term treatment with pregabalin on the number of patients with serious adverse events (RR 0.14, 95% CI: 0.01 to 1.31, 3 studies, very low certainty evidence).

**Pair-wise meta-analysis of short-term treatment with quetiapine compared with placebo**

*Symptoms of anxiety*

Short-term treatment with quetiapine may reduce symptoms of anxiety (SMD 0.54, 95% CI: 0.41 to 0.66, 3 studies, low certainty evidence).

*Serious adverse events*

The evidence is very uncertain about the effect of short-term treatment with quetiapine on the number of patients with serious adverse events (RR 1.19, 95% CI: 0.12 to 11.32, 3 studies, very low certainty evidence).

### Pair-wise meta-analysis of short-term treatment with agomelatine compared with placebo

*Symptoms of anxiety*

The evidence is very uncertain about the effect of short-term treatment with agomelatine on symptoms of anxiety (SMD 0.22, 95% CI: 0.21 to 0.66, 2 studies, very low certainty evidence).

*Serious adverse events*

The evidence is very uncertain about the short-term effect of agomelatin on the number of patients with serious adverse events (RR 1.84, 95% CI: 0.39 to 8.74, 2 studies, very low certainty evidence).

### Pair-wise meta-analysis of short-term treatment with hydroxyzine compared with placebo

*Symptoms of anxiety*

One study reported the outcome symptoms of anxiety and the evidence is very uncertain about the effect of short-term treatment with hydroxyzine on symptoms of anxiety. (SMD -0.21, 95% CI: -0.48 to 0.06, very low certainty evidence).

*Serious adverse events*

One study reported the outcome serious adverse events, and the evidence is very uncertain about the effect of short-term treatment with hydroxyzine on the number of patients with serious adverse events (RR 3.23, 95% CI: 0.13 to 78.34, very low certainty evidence).

### Pair-wise meta-analysis of short-term treatment with benzodiazepines compared with pregabalin

*Symptoms of anxiety*

The evidence is very uncertain about the effect of short-term treatment with benzodiazapines on symptoms of anxiety compared with pregabalin (SMD 0.04, 95% CI: -0.4 to 0.49, 4 studies, very low certainty evidence).

*Serious adverse events*

The evidence is very uncertain about the effect of short-term treatment with benzodiazepine on the number of patients with serious adverse events compared to pregabalin (RR 6.79, 95% CI: 1.08 to 42.82, 3 studies, very low certainty evidence).

None of the included studies investigated direct comparisons between pregabalin and quetiapine or between benzodiazpeines and quetiapine.

## Pair-wise meta-analyses of important outcomes

### Pair-wise meta-analysis of short-term treatment with benzodiazepines compared with placebo

#### Addiction – risk of discontinuation symptoms

One study reported data for the important outcome of addiction measures and the evidence is very uncertain about the effect of short-term treatment with benzodiazepines on addiction, measured as the risk of experiencing discontinuation symptoms (RR 8.89, 95% CI: 1.38 to 57.34, low certainty evidence).

#### Addiction – number of discontinuation symptoms

The evidence is very uncertain about the effect of short-term treatment with benzodiazepines on addiction - measured as withdrawal symptoms (SMD 0.37, 95% CI: 0.11 to 0.63, 4 studies, very low certainty evidence).

#### Suicide attempts/suicide

The evidence is very uncertain about the effect of short-term treatment with benzodiazepines on the number of patients with suicidal thoughts/attempts (RR 0.33, 95% CI: 0.01 to 7.92, 2 studies, very low certainty evidence).

#### Daytime drowsiness

Short-term treatment with benzodiazepines may increase the risk of daytime drowsiness (RR 2.21, 95% CI: 1.55 to 3.16, 10 studies, very low certainty evidence).

*Fractures*

One study reported data for the important outcome of fractures the evidence is very uncertain about the effect of short-term treatment with benzodiazepines on the number of patients with fractures (RR 3.09, 95% CI: 0.13 to 74.92, very low certainty evidence).

*Weight change*

The evidence is very uncertain about the effect of short-term treatment with benzodiazepines on weight change (RR 2.20, 95% CI: 1.05´to 4.62, 2 studies, very low certainty evidence).

*Cardiac adverse events*

One study reported the important outcome of cardiac adverse events, and the evidence is very uncertain about the effect of short-term treatment with benzodiazepines on cardiac side-effects (RR 0.00, 95 %CI: -0.02 to 0.02, very low certainty evidence).

*Dizziness*

The evidence is very uncertain about the effect of short-term treatment with benzodiazepines on dizziness (RR 1.21, 95% CI: 0.83 to 1.76, 7 studies, very low certainty evidence).

### Pair-wise meta-analysis of short-term treatment with pregabalin compared with placebo

*Addiction – number of discontinuation symptoms*

The evidence is very uncertain about the effect of short-term treatment with pregabalin on addiction, measured as withdrawal symptoms (SMD 0.22, 95% CI: 0.06 to 0.39, 4 studies, very low certainty evidence).

*Suicide attempts/suicide*

One study reported data on suicidal related events and the evidence is very uncertain about the effect of short-term treatment with pregabalin on the number of patients with suicidal thoughts/attempts (RR 0.17, 95% CI: 0.01 to 4.01, very low certainty evidence).

*Daytime drowsiness*

Short-term treatment with pregabalin may increase daytime drowsiness (RR 2.55, 95% CI 1.55 to 3.16, 10 studies, low certainty evidence).

*Cardiac adverse events*

One study found reported cardiac adverse events and the evidence is very uncertain about the effect of short-term treatment with pregabalin on cardiac side-effects (RR 0.00, 95% CI: -0.02 to 0.02, very low certainty evidence).

*Dizziness*

Short-term treatment with pregabalin may increase dizziness (RR 3.79, 95% CI: 2.39 to 6.01, 7 studies, low certainty evidence).

### Pair-wise meta-analysis of short-term treatment with quetiapine compared with placebo

*Addiction – number of discontinuation symptoms*

The evidence is very uncertain about the effect of short-term treatment with quetiapine on addiction, measured as withdrawal symptoms (SMD 0.22, 95% CI: 0.04 to 0.40, 2 studies, very low certainty evidence.

*Suicide attempts/suicide*

The evidence is very uncertain about the effect of short-term treatment with quetiapine on the number of patients with suicidal thoughts/attempts (RR 0.36, 95% CI: 0.02 to 8.04, 2 studies, very low certainty evidence).

*Daytime drowsiness*

The evidence is very uncertain about the effect of short-term treatment with quetiapine on daytime drowsiness (RR 1.56, 95% CI: 0.67 to 3.64, 3 studies, very low certainty evidence).

*Weight change*

One study reported the outcome of weight change, and the evidence is very uncertain about the effect of short-term treatment with quetiapine on weight change (RR 4.05, 95% CI: 0.94 to 17.44, low certainty evidence).

*Dizziness*

The evidence is very uncertain about the effect of short-term treatment with quetiapine on dizziness (RR 1.70, 95% CI: 1.16 to 2.4, 3 studies, very low certainty evidence).

*Extrapyramidal symptoms*

One study reported on the outcome of extrapyramidal symptoms and the evidence is very uncertain about the effect of short-term treatment with quetiapine on extrapyramidal symptoms RR 1.52, 95% CI: 0.73 to 3.17, low certainty evidence).

### Pair-wise meta-analysis of short-term treatment with agomelatin compared with placebo

*Addiction – number of discontinuation symptoms*

The evidence is very uncertain about the effect of short-term treatment with agomelatin on addiction, measured as withdrawal symptoms (SMD 0.06, 95% CI: -0.29 to 0.42, 2 studies, very low certainty evidence).

*Daytime drowsiness*

One study reported the outcome of daytime drowsiness, and the evidence is very uncertain about the effect of short-term treatment with agomelatin on daytime drowsiness (RR 1.30, 95% CI: 0.25 to 6.60, very low certainty evidence).

*Dizziness*

The evidence is very uncertain about the effect of short-term treatment with agomelatin on dizziness (RR 1.56, 95% CI: 0.54 to 4.56, 2 studies, very low certainty evidence).

### Pair-wise meta-analysis of short-term treatment with hydroxyzine compared with placebo

*Daytime drowsiness*

One study reported the outcomes of daytime drowsiness, and the evidence is very uncertain about the effect of short-term treatment with hydroxyzine on daytime drowsiness (RR 2.15, 95% CI: 0.40 to 11.51, very low certainty evidence).

### Pair-wise meta-analysis of short-term treatment with benzodiazepines compared with pregabalin

*Addiction – number of discontinuation symptoms*

The evidence is very uncertain about the effect of short-term treatment with benzodiazepines on addiction, assessed as the number of discontinuation symptoms compared to pregabalin (SMD 0.13, 95% CI: -0.05 to 0.3, 4 studies, very low certainty evidence).

*Suicide attempts/suicide*

One study reported the outcome of suicide or suicide attempts, and it is very uncertain about the effect of short-term treatment with benzodiazepines on suicidal thoughts/attempts compared to pregabalin (RR 0, -0.023 to 0.023, very low certainty evidence).

*Daytime drowsiness*

The evidence is very uncertain about the effect of short-term treatment with benzodiazepines on daytime drowziness compared to pregabalin (RR 1.53, 95% CI: 1.07 to 2.18, 3 studies, very low certainty evidence).

*Cardiac adverse effects*

One study reported the outcome cardiac adverse effects and the evidence is very uncertain about the effect of short-term treatment with benzodiazepines on cardiac side-effects compared to pr,egabalin (RR 0, -0.016 to 0.016, very low certainty evidence).

*Dizziness*

The evidence is very uncertain about the effect of short-term treatment with benzodiazepines on dizziness compared to pregabalin (RR 0.56, 95% CI: 0.31 to 0.98, 3 studies, very low certainty evidence).

# Supplementary Figure S8. Forest plots of pair-wise meta-analyses.

# Anxiety symptoms measured on the HAM-A

Benzodiazepine vs placebo, outcome: Anxiety symptoms - HAM-A.

**
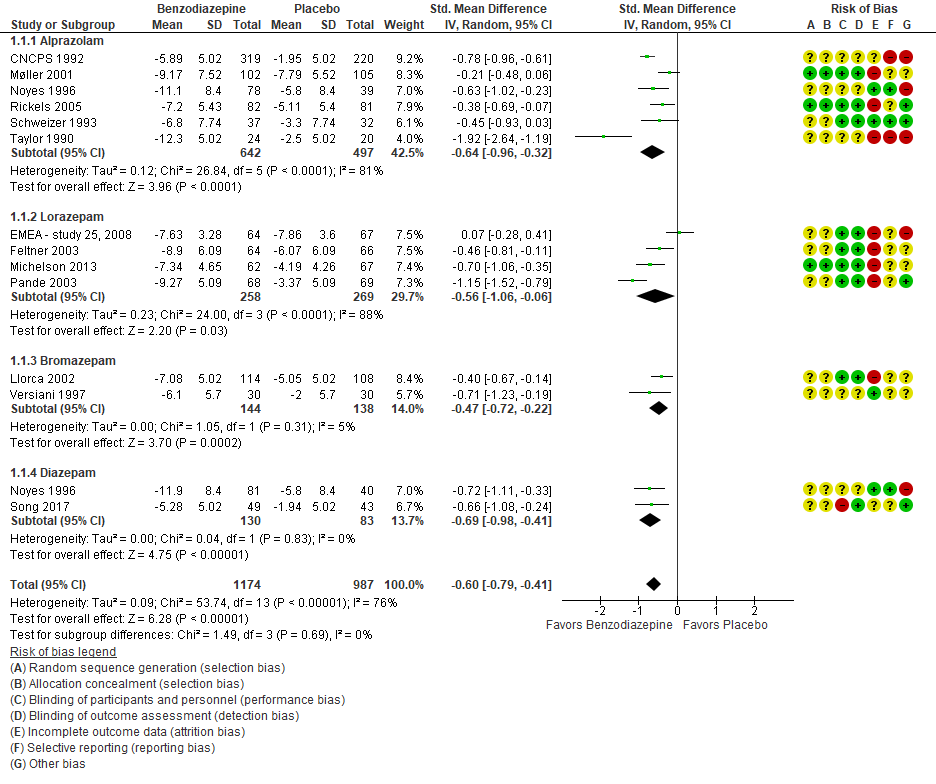
**

Pregabalin vs placebo, outcome: Anxiety symptoms - HAM-A.


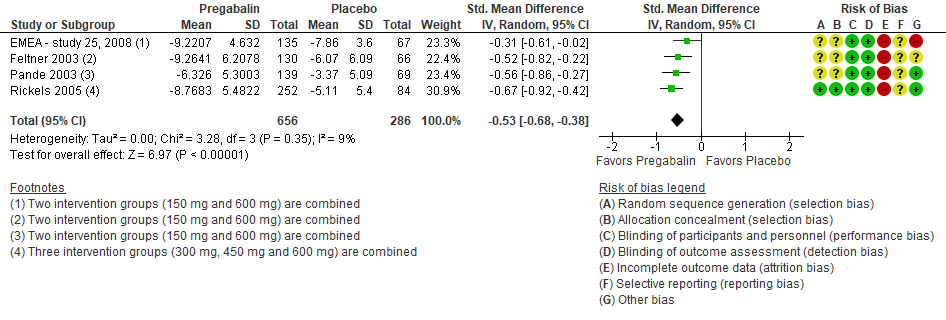


Quetiapine vs placebo, outcome: Anxiety symptoms - HAM-A.


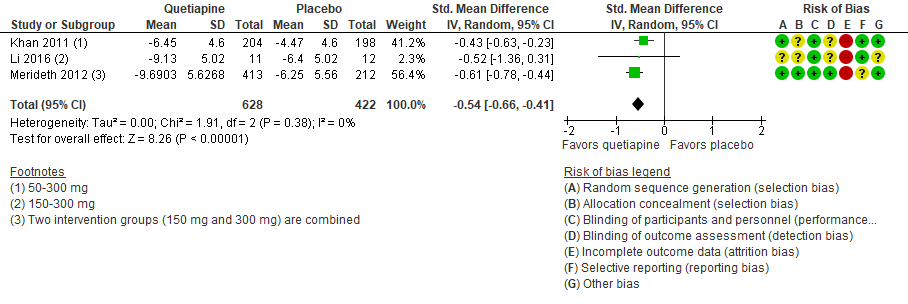


Agomelatine vs placebo, outcome: Anxiety symptoms - HAM-A.


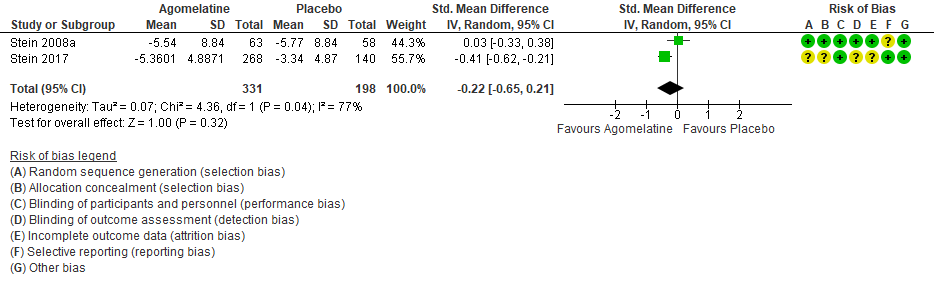


Hydroxyzine vs placebo, outcome: Anxiety symptoms - HAM-A.

**
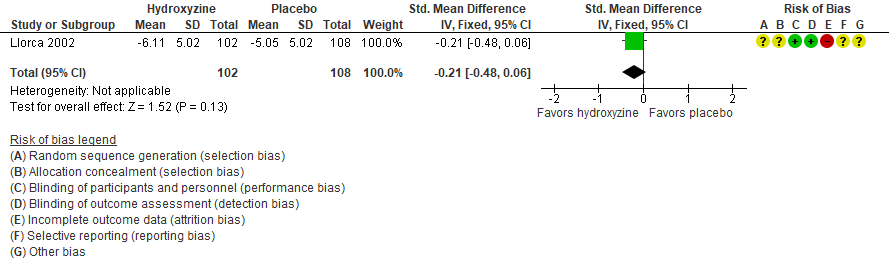
**

Benzodiazepine vs pregabalin, outcome: Anxiety symptoms - HAM-A


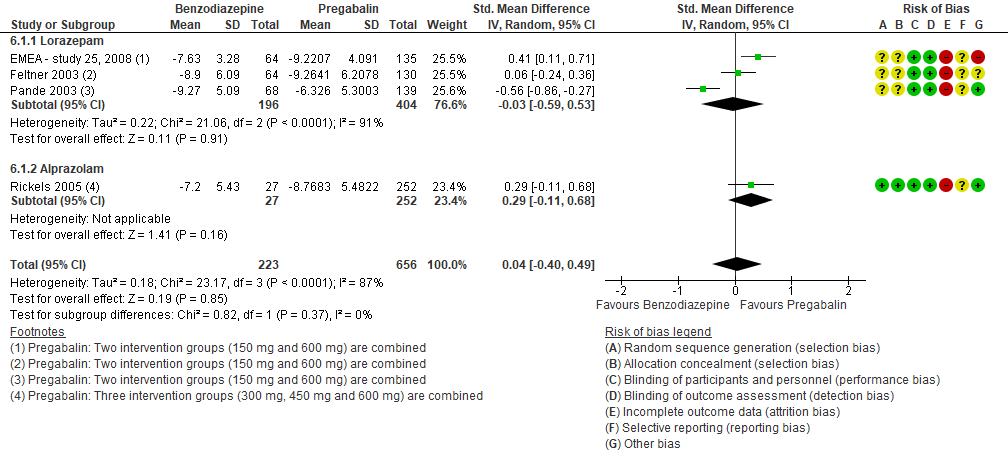


**Function of daily living/disability**

**Benzodiazepine vs placebo, outcome: 1.4 Function - Work.**

**
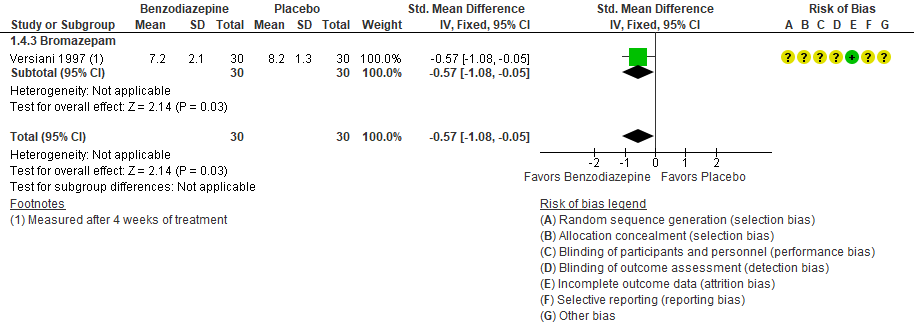
**

**Benzodiazepine vs placebo, outcome: 1.5 Function – Social**


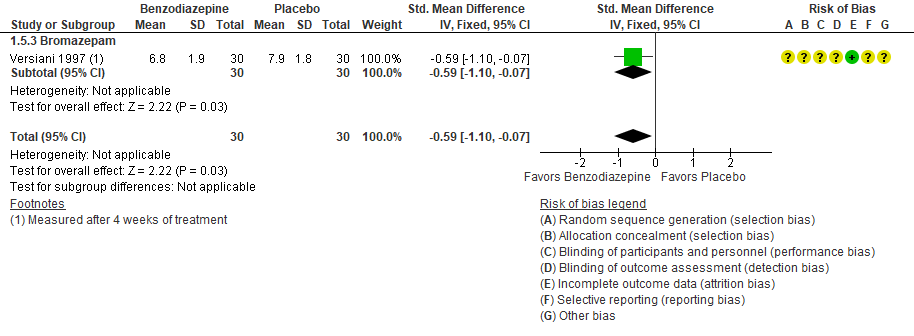


**Benzodiazepine vs placebo, outcome: 1.6 Function – Family**


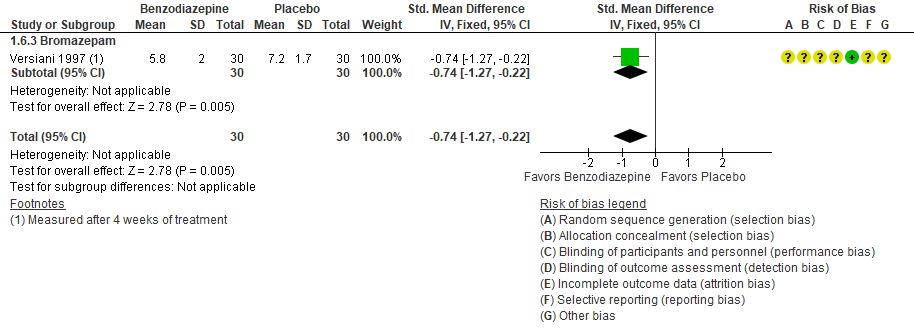


**Serious adverse events**

**Benzodiazepine vs placebo, outcome: 1.7 Serious adverse events_risk ratio**


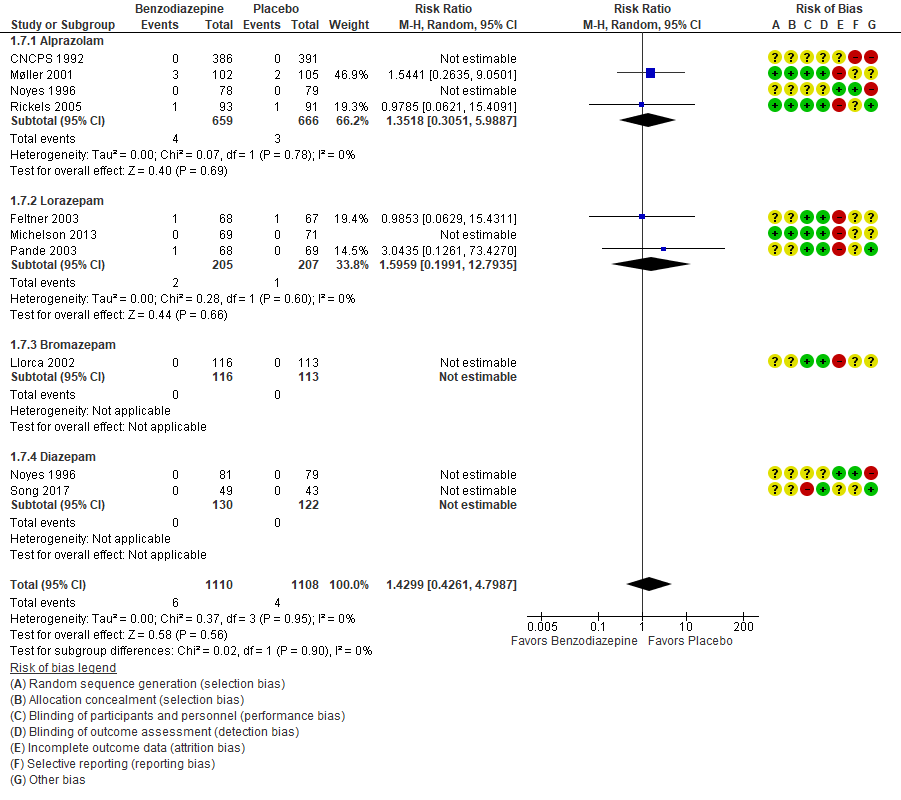


**Benzodiazepine vs placebo, outcome: 1.8 Serious adverse events_risk difference.**

**
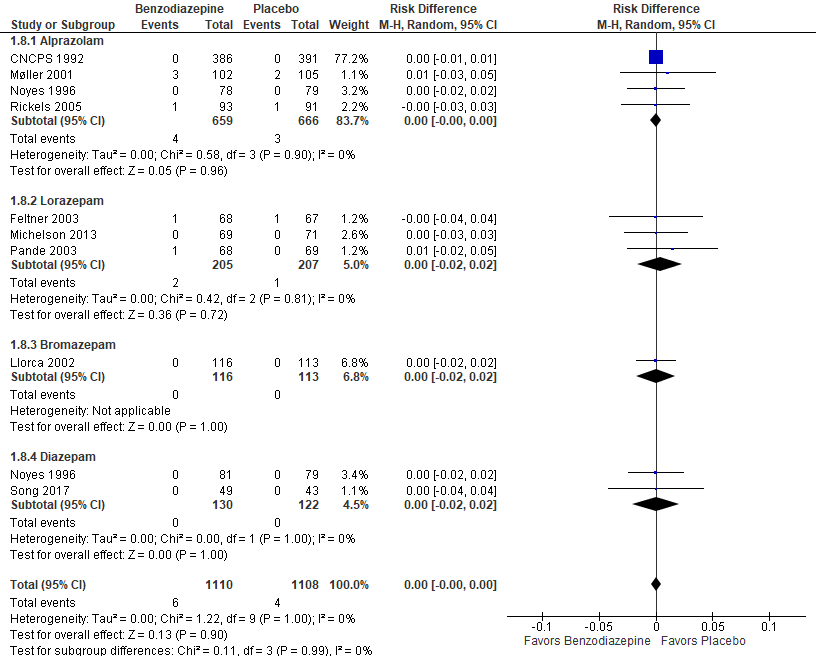
**

**Pregabalin vs placebo, outcome: 2.2 Serious adverse events_risk difference.**

**
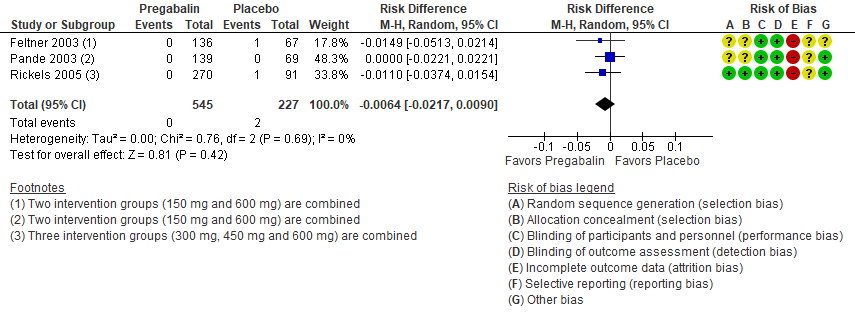
**

**Pregabalin vs placebo, outcome: 2.3 Serious adverse events_risk ratio**


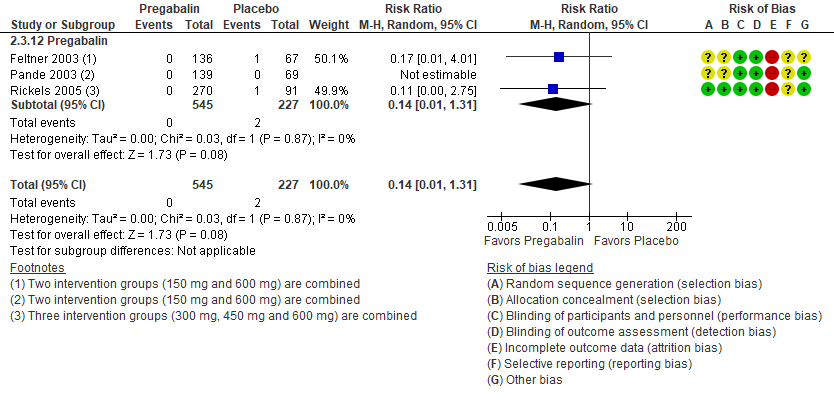


**Quetiapine vs placebo, outcome: 3.2 Serious adverse events_risk ratio.**


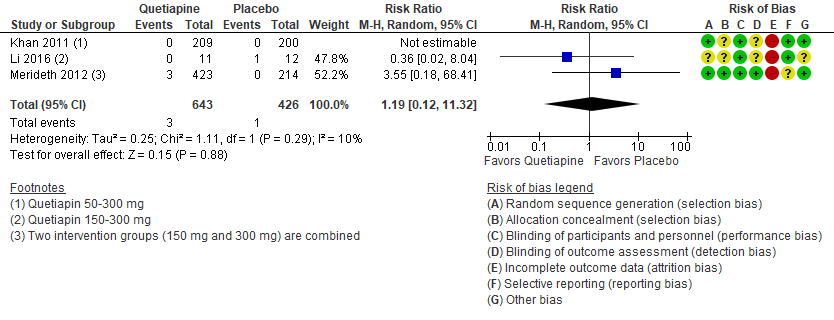


**Quetiapine vs placebo, outcome: 3.3 Serious adverse events_risk difference.**


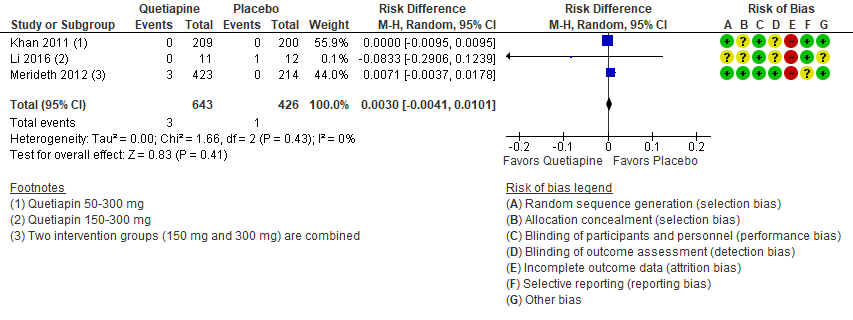


**Agomelatin vs placebo, outcome: 4.2 Serious adverse events_risk ratio.**


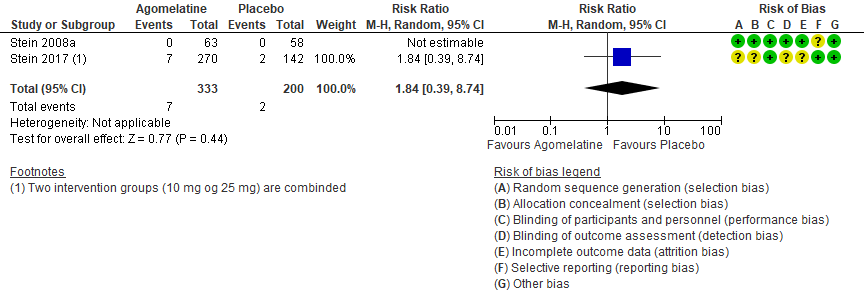


**Agomelatin vs placebo, outcome: 4.3 Serious adverse events_risk difference.**

**
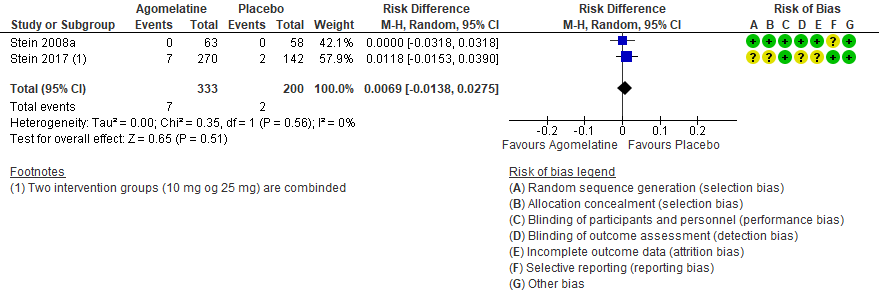
**

**Hydroxyzine vs placebo, outcome: 5.2 Serious adverse events_risk ratio.**


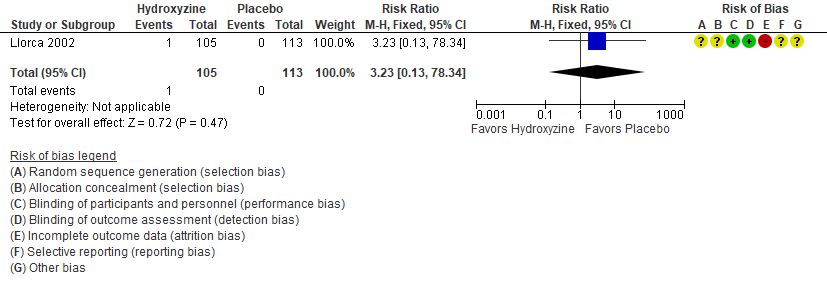


**Hydroxyzine vs placebo, outcome: 5.3 Serious adverse events_risk difference.**


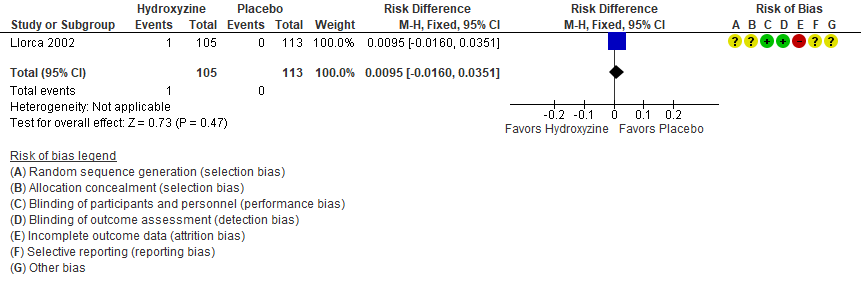


**Benzodiazepine vs pregabalin, outcome: 6.2 Serious adverse events_risk ratio**


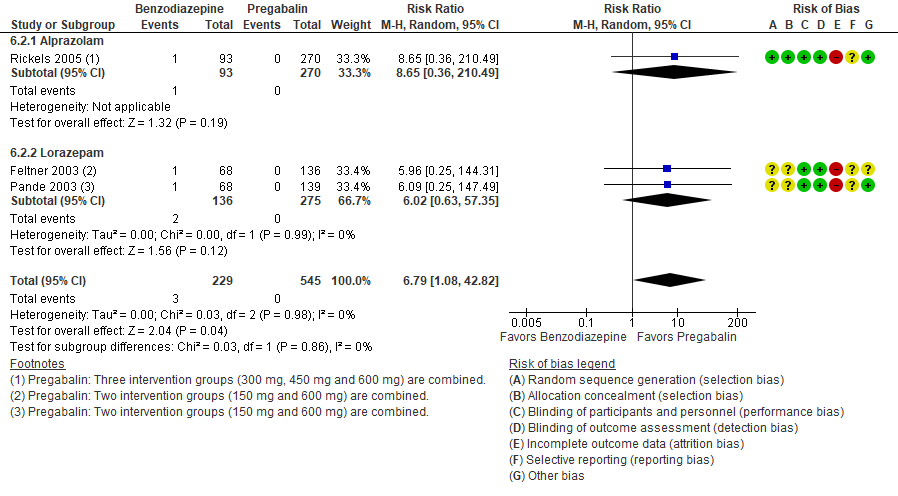


**Benzodiazepine vs pregabalin, outcome: 6.3 Serious adverse events_risk diference**

**
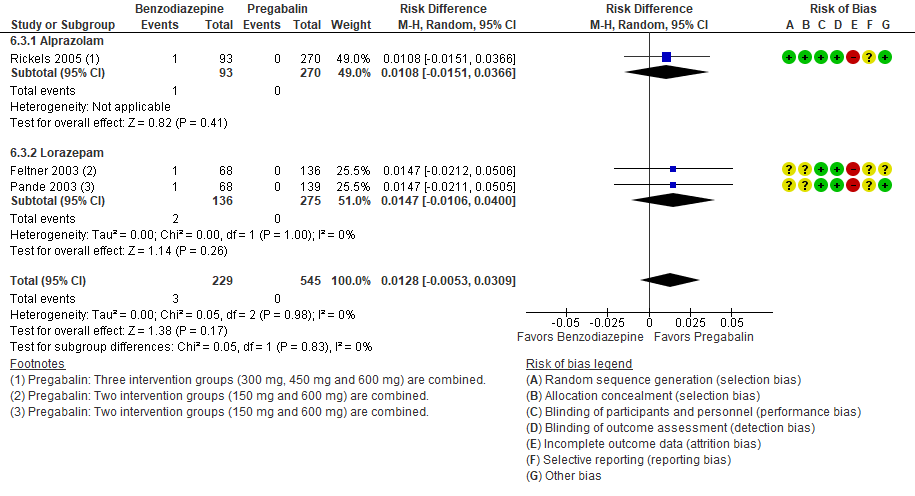
**

**Important outcomes**

**Benzodiazepine vs placebo, outcome: 1.2 Addiction - Withdrawal symptoms**

**
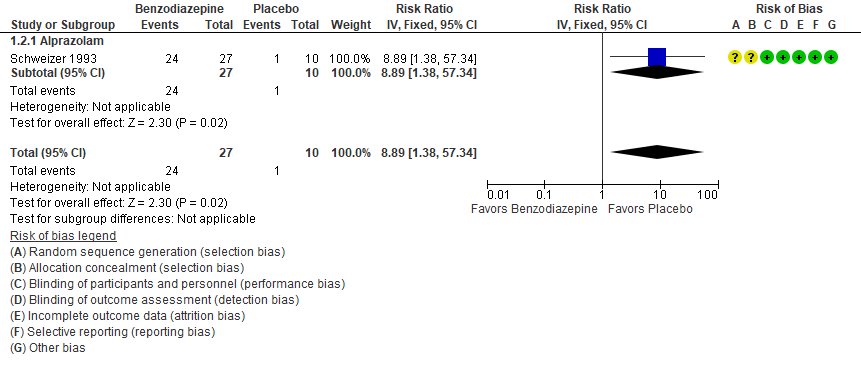
**

**Benzodiazepine vs placebo, outcome: 1.3 Addiction withdrawal symptoms**

**
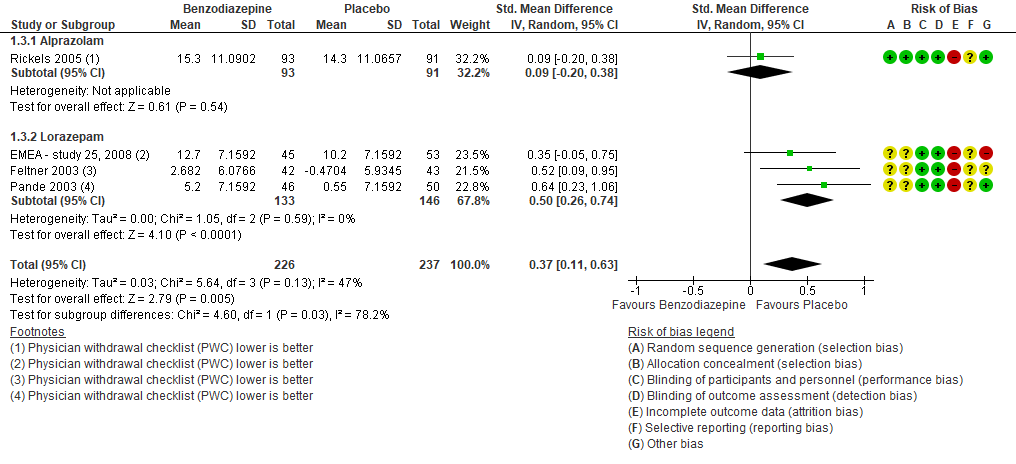
**

**Benzodiazepine vs placebo, outcome: 1.9 Suicidal thoughts/attempts_risk ratio**

**
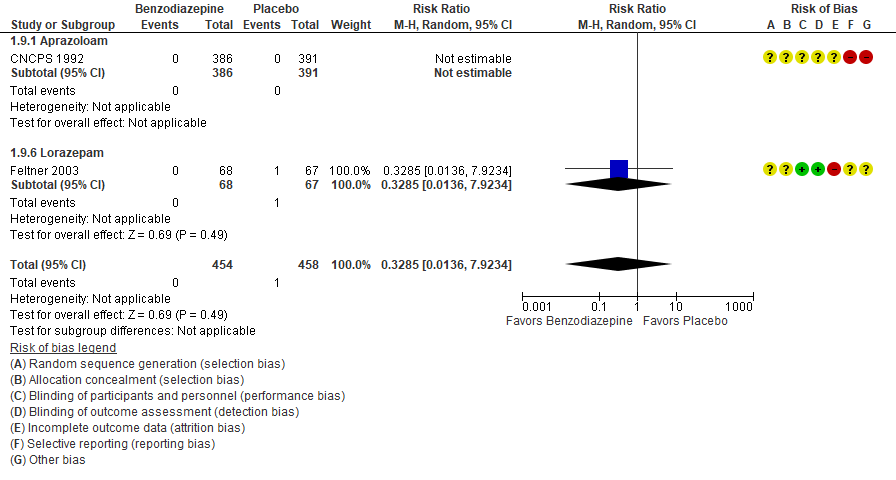
**

**Benzodiazepine vs placebo, outcome: 1.10 Suicidal thoughts/attempts_risk difference**

**
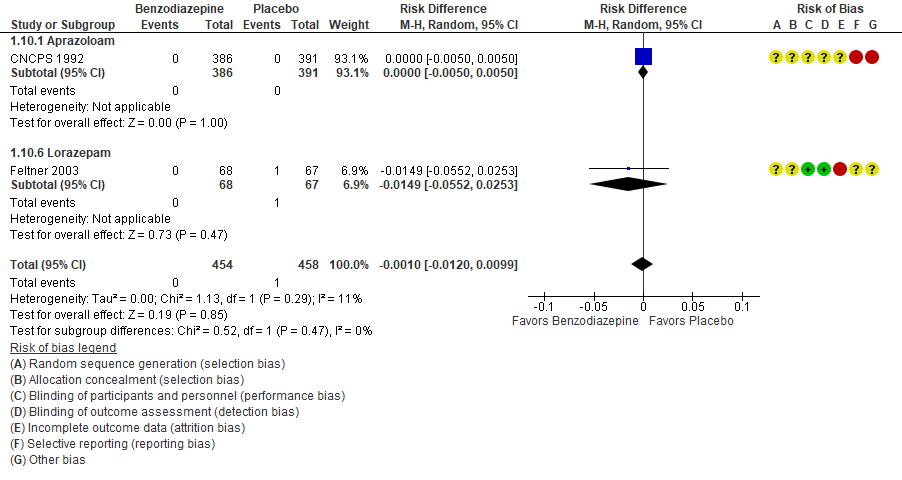
**

**Benzodiazepine vs placebo, outcome: 1.11 Daytime drowsiness**

**
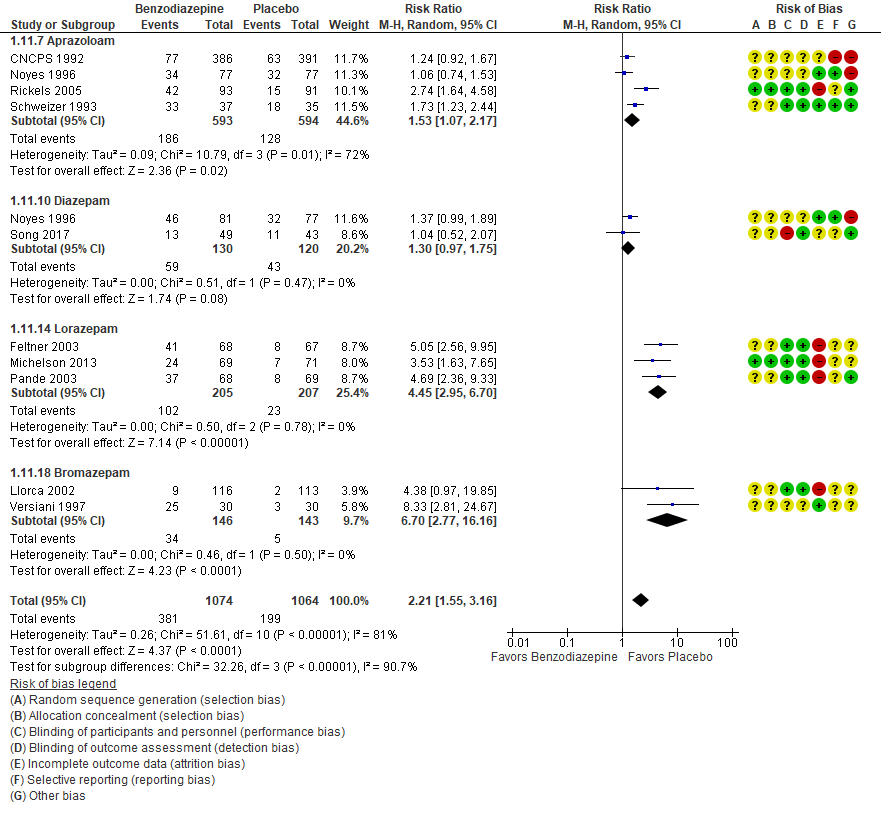
**

**Benzodiazepine vs placebo, outcome: 1.12 Fractures_risk ratio**

**
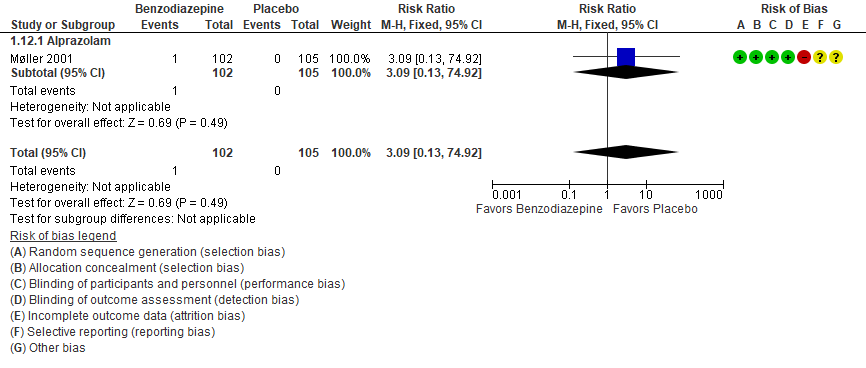
**

**Benzodiazepine vs placebo, outcome: 1.13 Fractures_ risk difference**

**
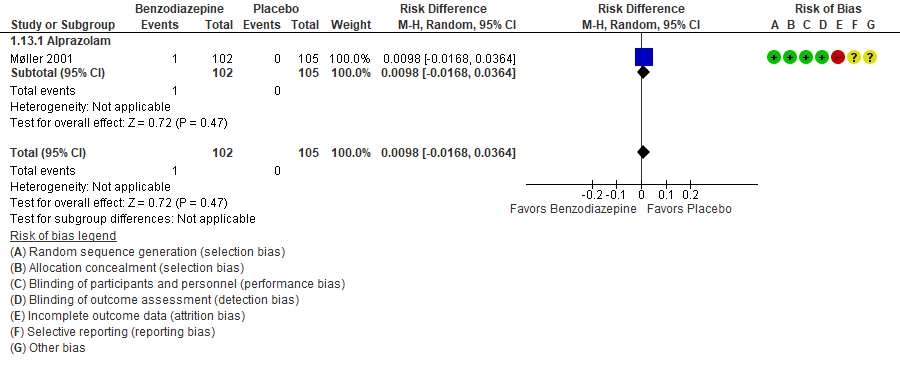
**

**Benzodiazepine vs placebo, outcome: 1.14 Weight change**

**
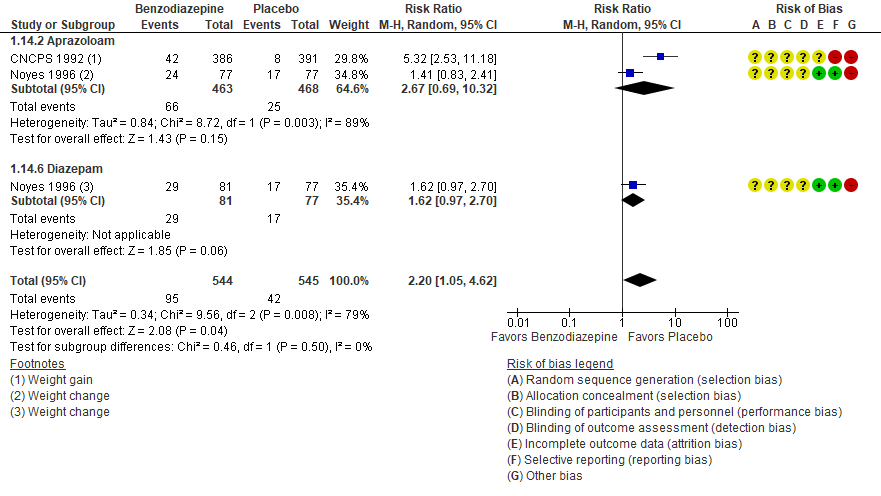
**

**Benzodiazepine vs placebo, outcome: 1.15 Cardiac side-effects_risk difference.**

**
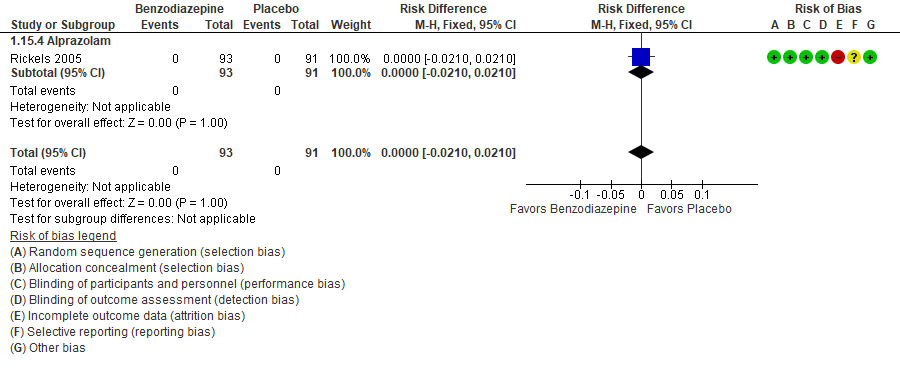
**

**Benzodiazepine vs placebo, outcome: 1.16 Dizziness**

**
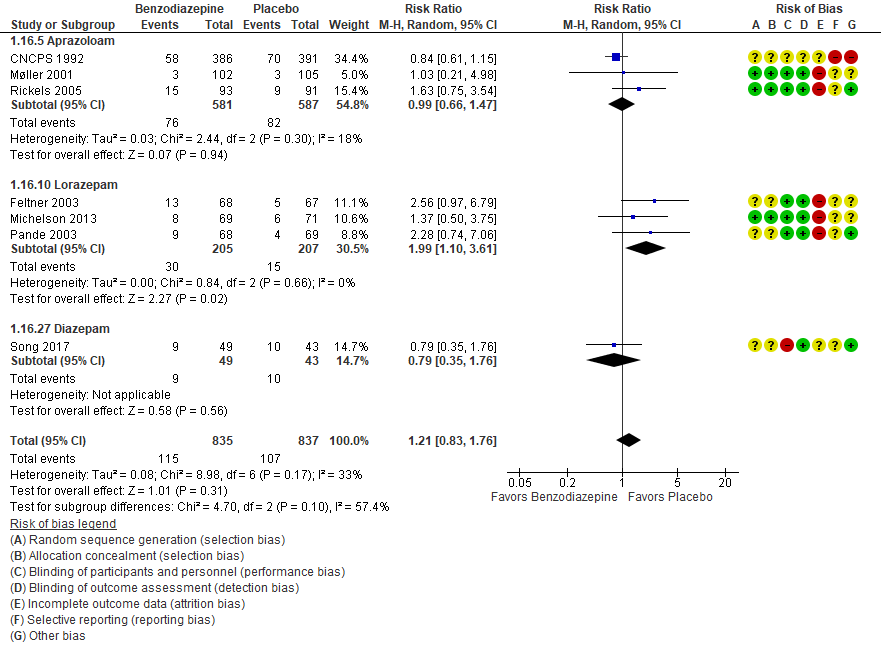
**

**Pregabalin vs placebo, outcome: 2.4 Suicidal thoughts/attempts_risk ratio**

**
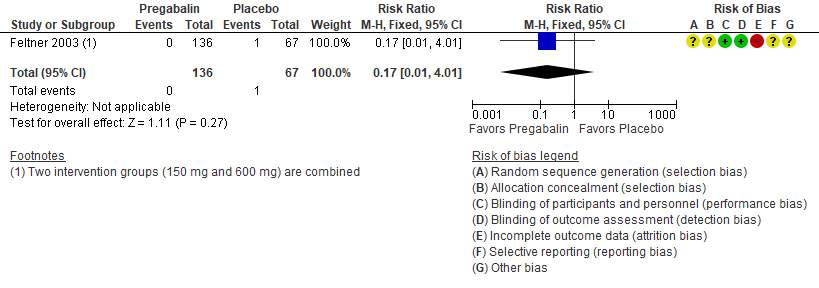
**

**Pregabalin vs placebo, outcome: 2.5 Suicidal thoughts/attempts_risk difference**

**
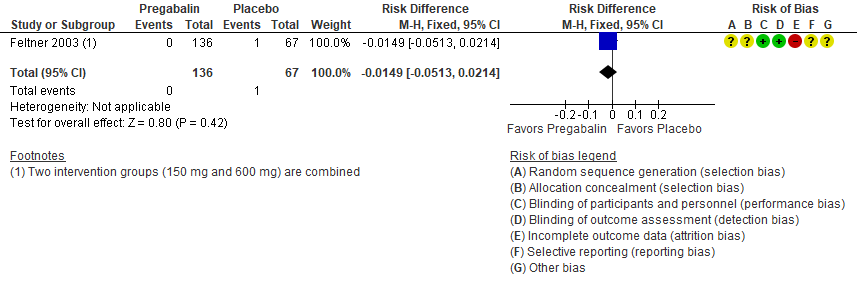
**

**Pregabalin vs placebo, outcome: 2.6 Daytime drowsiness**

**
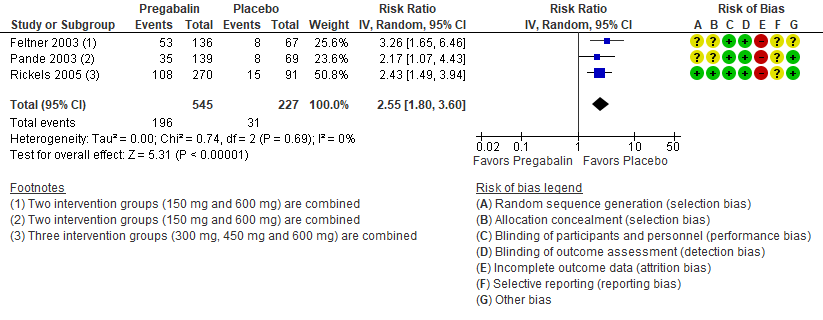
**

**Pregabalin vs placebo, outcome: 2.7 Cardiac side-effects_risk difference**

**
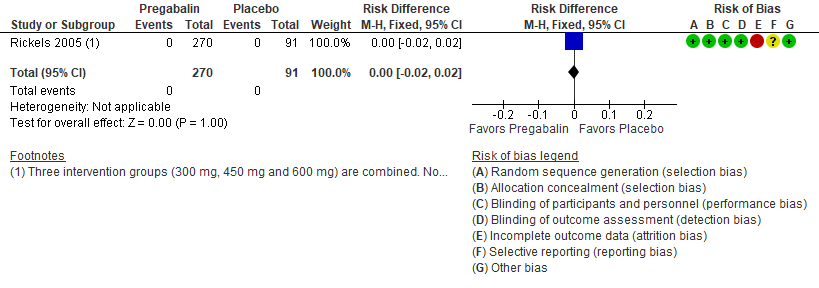
**

**Pregabalin vs placebo, outcome: 2.8 Addiction - withdrawal symptoms**

**
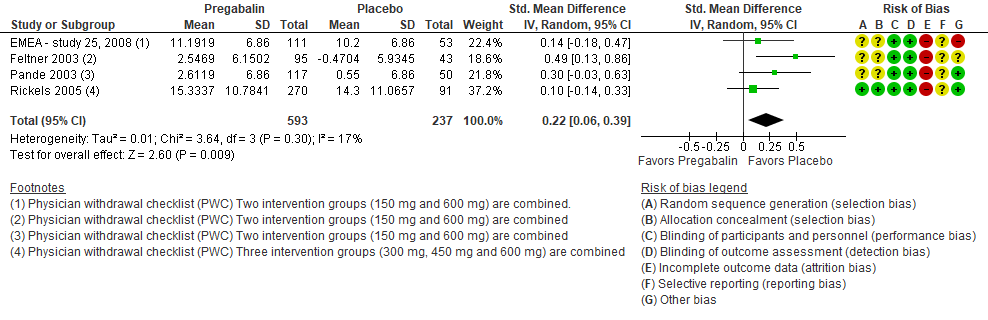
**

**Pregabalin vs placebo, outcome: 2.9 Dizziness**

**
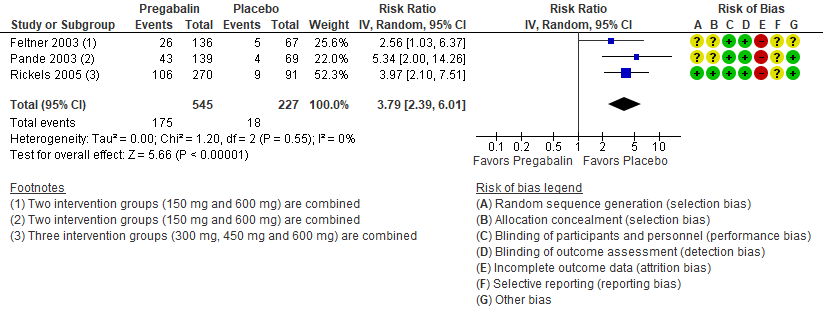
**

**Quetiapine vs placebo, outcome: 3.4 Addiction - Withdrawal symptoms**

**
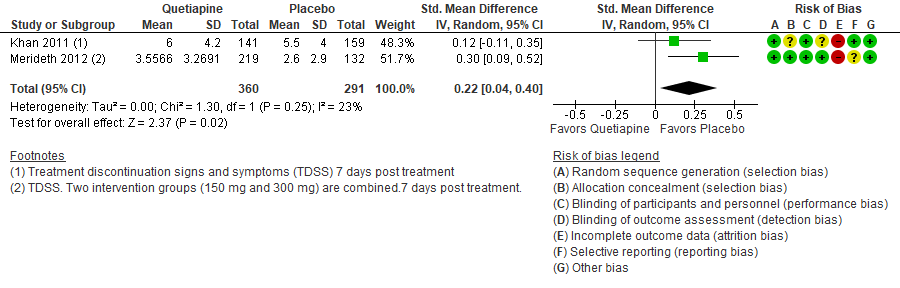
**

**Quetiapine vs placebo, outcome: 3.5 Suicidal thoughts/attempts**

**
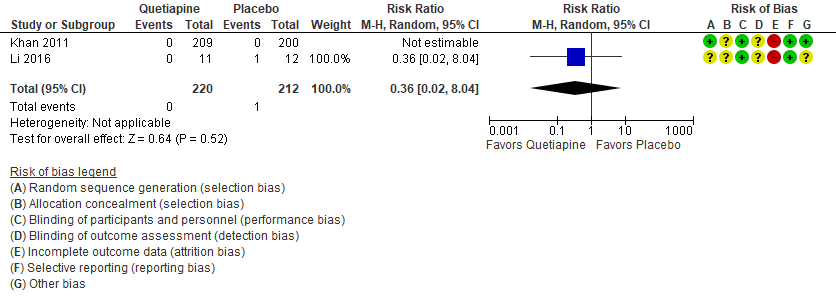
**

**Quetiapine vs placebo, outcome: 3.6 Daytime drowsiness**

**
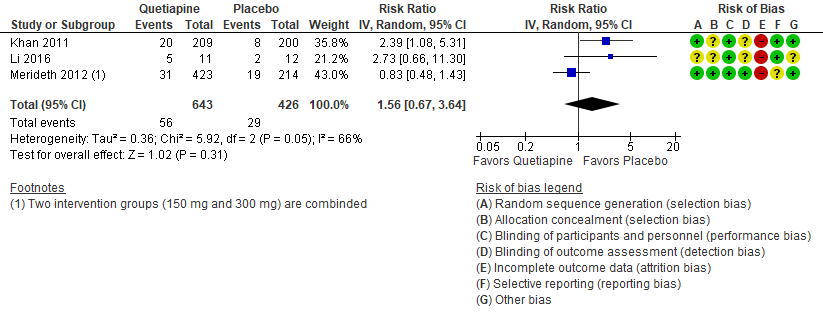
**

**Quetiapine vs placebo, outcome: 3.7 Weight change**

**
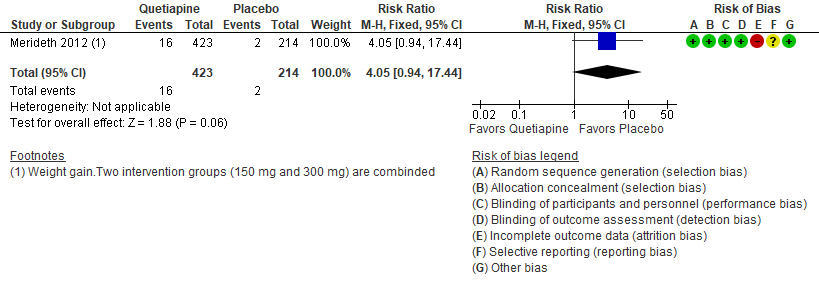
**

**Quetiapine vs placebo, outcome: 3.8 Extrapyramidal symptoms**

**
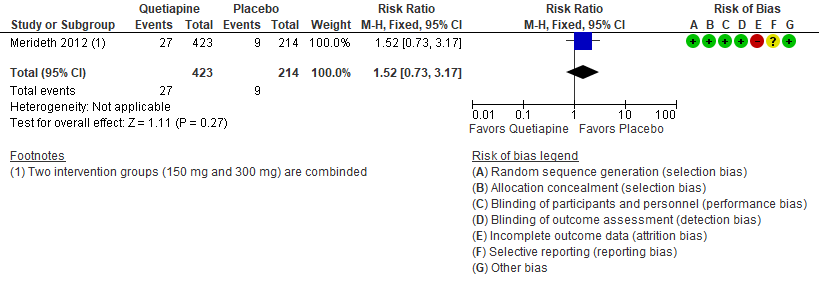
**

**Agomelatine vs placebo, outcome: 4.4 Daytime drowsiness**

**
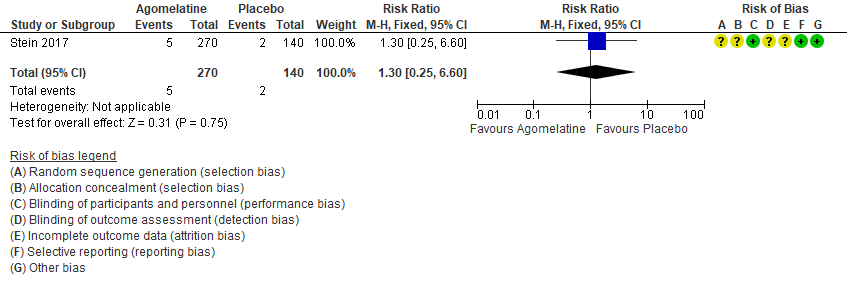
**

**Agomelatine vs placebo, outcome: 4.5 Addiction withdrawal symptoms**

**
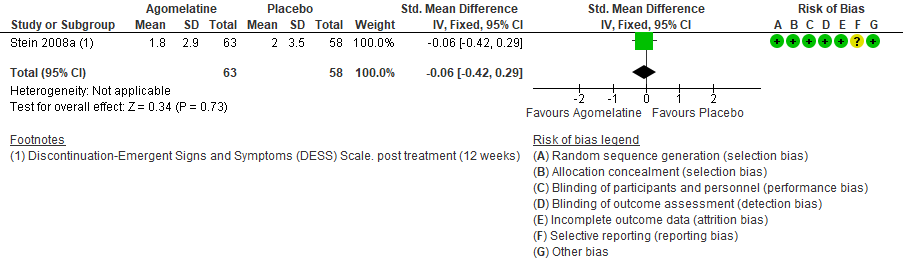
**

**Agomelatine vs placebo, outcome: 4.6 Dizziness**

**
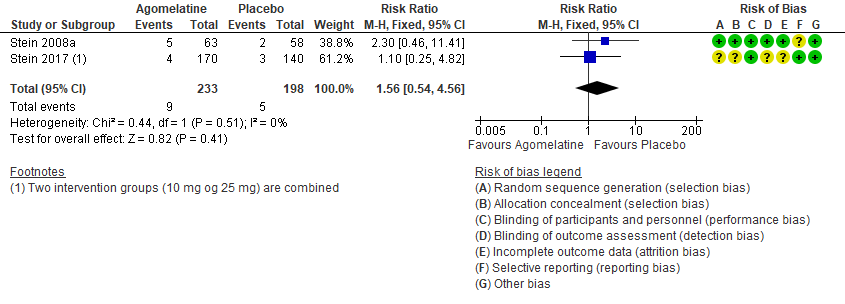
**

**Hydroxyzine vs placebo, outcome: 5.4 Daytime drowsiness**

**
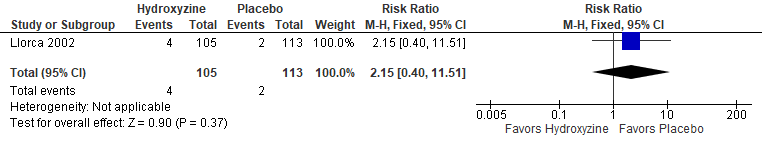
**

**Benzodiazepine vs pregabalin, outcome: 6.4 Addiction - Withdrawal symptoms**

**
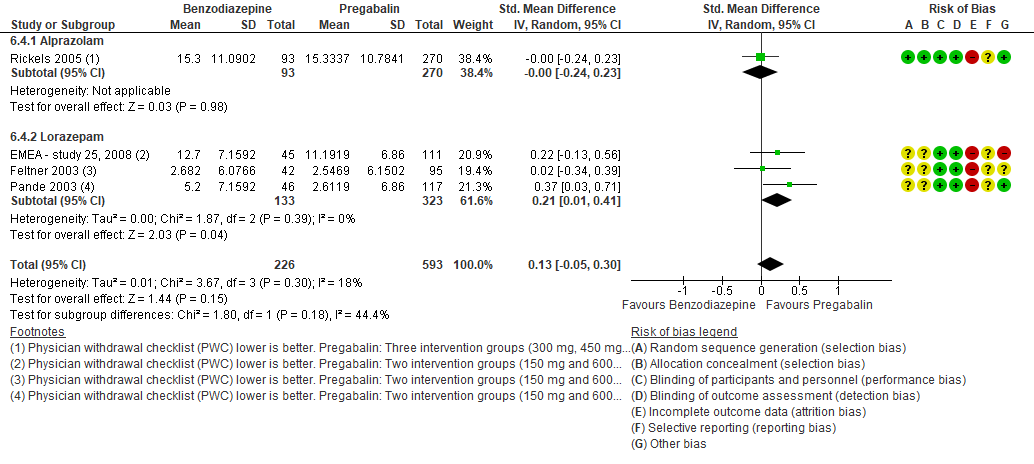
**

**Benzodiazepine vs pregabalin, outcome: 6.5 Suicidal thoughts/attempts**

**
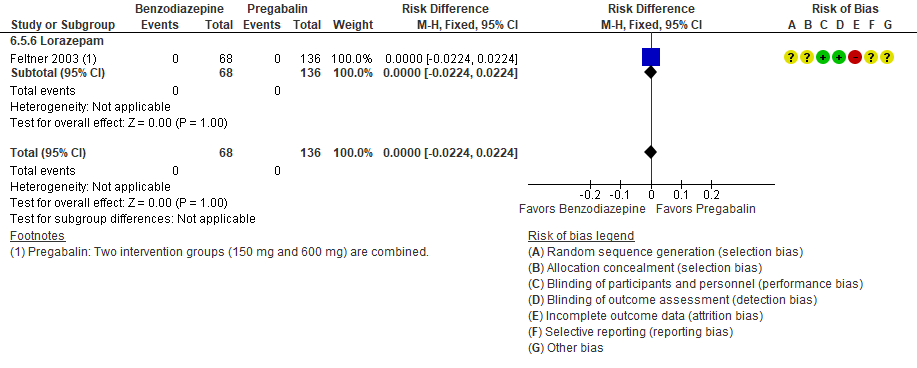
**

**Benzodiazepine vs pregabalin, outcome: 6.6 Daytime drowsiness**

**
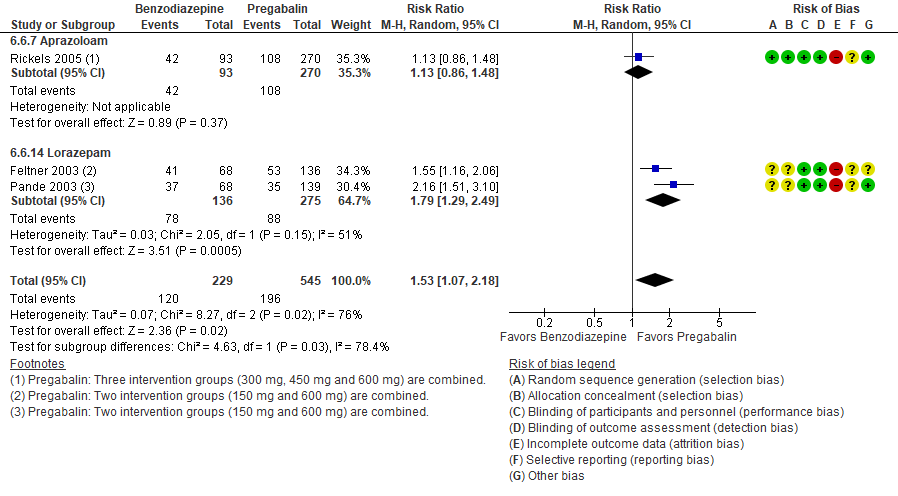
**

**Benzodiazepine vs pregabalin, outcome: 6.7 Cardiac side-effects_risk difference**

**
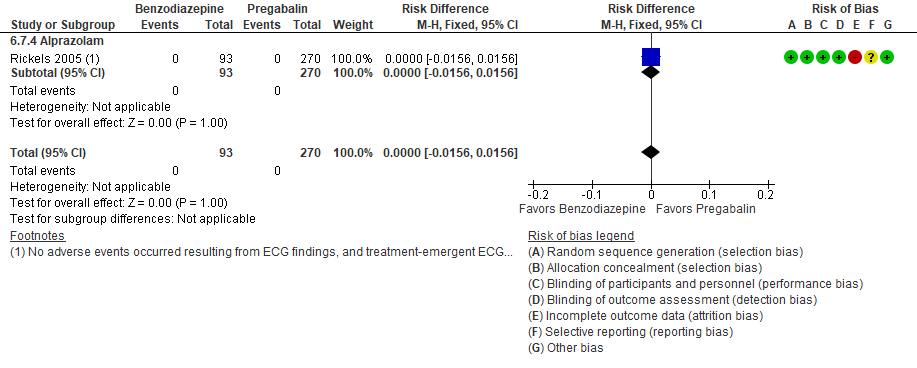
**

**Benzodiazepine vs pregabalin, outcome: 6.8 Dizziness**

**
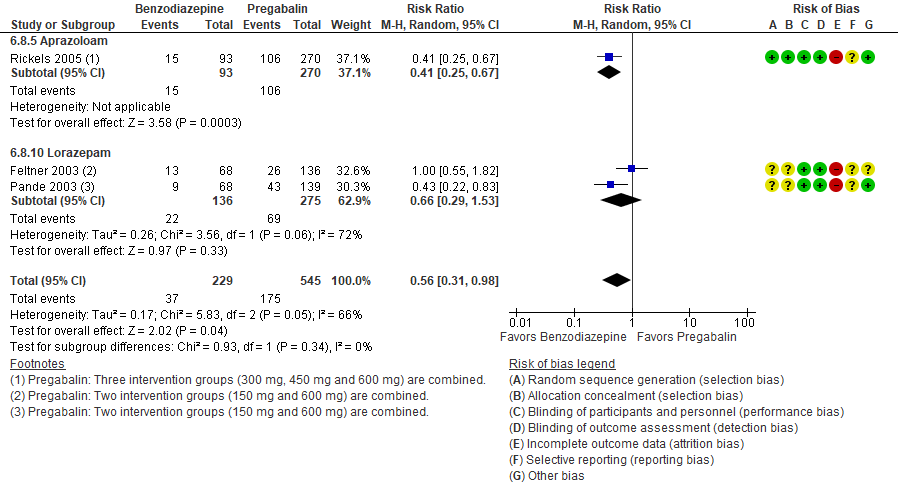
**

# Supplementary Table S5. Summary of Findings Tables.

|  | | | | | | |
| --- | --- | --- | --- | --- | --- | --- |
| **GRADE Summary of Findings Table. Benzodiazepines compared to placebo for short-term treatment of newly-onset symptoms of anxiety and distress** | | | | | | |
| **Patient or population:** Non-hospitalised adults with newly-onset of symptoms of anxiety and distress in need of short-term pharmacological treatment (maximum up to 4 weeks).  **Setting:** Non-hospitalised  **Intervention:** Benzodiazepine  **Comparison:**Placebo | | | | | | |
| Outcomes | **Anticipated absolute effects^*^** (95% CI) | | Relative effect (95% CI) | № of participants (studies) | Certainty of the evidence (GRADE) | Comments |
|  | **Risk with placebo** | **Risk with Benzodiazepine** |  |  |  |  |
| Anxiety symptoms (critical outcome) assessed with: HAM-A | - | SMD **0.6 lower** (0.79 lower to 0.41 lower) | - | 2161 (13 RCTs) | ⨁⨁◯◯ Low^a,b,c,d,e^ | Short-term treatment with benzodiazepine may reduce symptoms of anxiety (critical outcome). |
| Addiction - withdrawal symptoms | 100 per 1.000 | **789 more per 1.000** (570 more to 1.000 more) | **RR 8.89** (1.38 to 57.34) | 37 (1 RCT) | ⨁◯◯◯ Very low^a,c,e,f,g,h,i^ | The evidence is very uncertain about the effect of short-term treatment with benzodiazepine on addiction - measured as number of patients with withdrawal symptoms. |
| Addiction - withdrawal symptoms | - | SMD **0.37 higher** (0.11 higher to 0.63 higher) | - | 463 (4 RCTs) | ⨁◯◯◯ Very low^a,b,c,e,g,h^ | The evidence is very uncertain about the effect of short-term treatment with benzodiazepine on addiction - measured as withdrawal symptoms. |
| Function – Work  (critical outcome) | - | SMD **0.57 lower** (1.08 lower to 0.05 lower) | - | 60 (1 RCT) | ⨁◯◯◯ Very low^a,b,e,f,h,i,j,k^ | The evidence is very uncertain about the effect of short-term treatment with benzodiazepine on function - work dimension  (critical outcome) |
| Function – Social  (critical outcome) | - | SMD **0.59 lower** (1.1 lower to 0.07 lower) | - | 60 (1 RCT) | ⨁◯◯◯ Very low^a,b,e,f,h,i,j,k^ | The evidence is very uncertain about the effect of short-term treatment with benzodiazepine on function - social dimension  (critical outcome) |
| Function – Family  (critical outcome) | - | SMD **0.74 lower** (1.27 lower to 0.22 lower) | - | 60 (1 RCT) | ⨁◯◯◯ Very low^a,b,e,f,h,i,j,k^ | The evidence is very uncertain about the effect of short-term treatment with benzodiazepine on function - family dimension (critical outcome) |
| Serious adverse events (critical outcome) | 4 per 1.000 | **2 more per 1.000** (2 fewer to 15 more) | **RR 1.43** (0.43 to 4.80) | 2218 (9 RCTs) | ⨁◯◯◯ Very low^a,c,e,f,g,l^ | The evidence is very uncertain about the effect of short-term treatment with benzodiazepine on number of patients with serious adverse events (critical outcome) |
| Suicidal thoughts/attempts | 2 per 1.000 | **1 fewer per 1.000**** (12 fewer to 10 more) | **RR 0.33** (0.01 to 7.92) | 912 (2 RCTs) | ⨁◯◯◯ Very low^a,c,e,f,g,h,l^ | The evidence is very uncertain about the effect of short-term treatment with benzodiazepine on the number of patients with suicidal thoughts/attempts. |
| Daytime drowsiness | 187 per 1.000 | **226 more per 1.000** (103 more to 404 more) | **RR 2.21** (1.55 to 3.16) | 2138 (10 RCTs) | ⨁⨁◯◯ Low^a,d,e,f,g^ | Short-term treatment with benzodiazepine may increase the number of patients with daytime drowsiness. |
| Fractures | 0 per 1.000 | **10 more per 1.000**** (17 fewer to 36 more) | **RR 3.09** (0.13 to 74.92) | 207 (1 RCT) | ⨁◯◯◯ Very low^c,e,g,h,i,l^ | The evidence is very uncertain about the effect of short-term treatment with benzodiazepine on the number of patients with fractures |
| Weight change | 77 per 1.000 | **92 more per 1.000** (4 more to 279 more) | **RR 2.20** (1.05 to 4.62) | 1089 (2 RCTs) | ⨁◯◯◯ Very low^a,b,d,e,f,g,h,j^ | The evidence is very uncertain about the effect of short-term treatment with benzodiazepine on weight change. |
| Cardiac side-effects risk difference | 0 per 1.000 | **0 more per 1.000**** (21 fewer to 21 more) | **RD 0.00**  (-0.021.to 0.021) | 184 (1 RCT) | ⨁◯◯◯ Very low^c,e,g,i,m^ | The evidence is very uncertain about the effect of short-term treatment with benzodiazepine on cardiac side-effects |
| Dizziness | 128 per 1.000 | **127 more per 1.000** (22 fewer to 92 more) | **RR 1.21** (0.83 to 1.76) | 1672 (7 RCTs) | ⨁◯◯◯ Very low^a,c,e,f,g,h^ | The evidence is very uncertain about the effect of short-term treatment with benzodiazepine on dizziness. |
| *The risk in the intervention group (and its 95% confidence interval) is based on the assumed risk in the comparison group and the relative effect of the intervention (and its 95% CI).  ** The absolute numbers are calculated based on a risk difference analysis.  **Abbreviation: CI:** Confidence interval; **GRADE**: Grades of Recommendation, Assessment, Development and Evaluation; **SMD:** Standardised mean difference; **RR:** Risk ratio; **RD:** risk difference | | | | | | |
| **GRADE Working Group grades of evidence** **High certainty:** we are very confident that the true effect lies close to that of the estimate of the effect. **Moderate certainty:** we are moderately confident in the effect estimate: the true effect is likely to be close to the estimate of the effect, but there is a possibility that it is substantially different. **Low certainty:** our confidence in the effect estimate is limited: the true effect may be substantially different from the estimate of the effect. **Very low certainty:** we have very little confidence in the effect estimate: the true effect is likely to be substantially different from the estimate of effect. | | | | | | |

#### Explanations

a. Inadequate concealment of allocation during randomization process

b. Inadequate/lack of blinding of participants and personnel

c. Incomplete data and/or large loss to follow up

d. The magnitude of statistical heterogeneity was high

e. Differences between the population of interest and those studied.

f. Inadequate sequence generation/ generation of comparable groups

g. Differences between the intervention/comparator of interest and those studied. We were interested in short-term treatment (up to 4 weeks). The outcome was measured after longer term treatment.

h. Wide confidence intervals

i. Only data from one study

j. Inadequate/lack of blinding of outcome assessors

k. Selective outcome reporting

l. Few events

m. Low number of patients and no events

|  | | | | | | |
| --- | --- | --- | --- | --- | --- | --- |
| **GRADE Summary of Findings Table. Pregabalin compared to placebo for short-term treatment of anxiety and distress** | | | | | | |
| **Patient or population:** Non-hospitalised adults with new-onset of symptoms of anxiety in need of short-term pharmacological treatment (maximum up to 4 weeks).  **Setting:** Non-hospitalised  **Intervention:** Pregabalin  **Comparison:** Placebo | | | | | | |
| Outcomes | **Anticipated absolute effects^*^** (95% CI) | | Relative effect (95% CI) | № of participants (studies) | Certainty of the evidence (GRADE) | Comments |
|  | **Risk with placebo** | **Risk with Pregabalin** |  |  |  |  |
| Anxiety symptoms  (critical outcome) assessed with: HAM-A | - | SMD **0.53 lower** (0.68 lower to 0.38 lower) | - | 942 (4 RCTs) | ⨁⨁◯◯ Low^a,b,c,d,e^ | Short-term treatment with pregabalin may reduce symptoms of anxiety (critical outcome) |
| Serious adverse events  (critical outcome) | 9 per 1.000 | **6 fewer per 1.000**** 22 fewer to 9 more) | **RR 0.14** (0.01 to 1.31) | 772 (3 RCTs) | ⨁◯◯◯ Very low^a,b,c,e,f,g^ | The evidence is very uncertain about the effect of short-term treatment with pregabalin on the number of patients with serious adverse events (critical outcome) |
| Suicidal thoughts/attempts | 15 per 1.000 | **15 fewer per 1.000**** (51 fewer to 21 more) | **RR 0.17** (0.01 to 4.01) | 203 (1 RCT) | ⨁◯◯◯ Very low^a,b,c,e,f,h,i^ | The evidence is very uncertain about the effect of short-term treatment with pregabalin on the number of patients with suicidal thoughts/attempts. |
| Daytime drowsiness | 137 per 1.000 | **243 more per 1.000** (126 more to 408 more) | **RR 2.55** (1.80 to 3.60) | 772 (3 RCTs) | ⨁⨁◯◯ Low^a,b,c,e,f^ | Short-term treatment with pregabalin may increase daytime drowsiness. |
| Cardiac side-effects_risk difference | 0 per 1.000 | **0 per 1.000**** (16 fewer to 16 more) | **RD 0.00** (-0.0159 to 0.0159) | 361 (1 RCT) | ⨁◯◯◯ Very low^c,e,f,h,j^ | The evidence is very uncertain about the effect of short-term treatment with pregabalin on cardiac side-effects |
| Addiction - withdrawal symptoms | - | SMD **0.22 higher** (0.06 higher to 0.39 higher) | - | 830 (4 RCTs) | ⨁◯◯◯ Very low^a,b,c,e,f,k^ | The evidence is very uncertain about the effect of short-term treatment with pregabalin on addiction - measured as withdrawal symptoms. |
| Dizziness | 79 per 1.000 | **220 more per 1.000** (110 more to 396 more) | **RR 3.79** (2.39 to 6.01) | 772 (3 RCTs) | ⨁⨁◯◯ Low^a,b,c,e,f^ | Short-term treatment with pregabalin may increase dizziness. |
| *The risk in the intervention group (and its 95% confidence interval) is based on the assumed risk in the comparison group and the relative effect of the intervention (and its 95% CI).  ** The absolute numbers are calculated based on a risk difference analysis. | | | | | | |
| **GRADE Working Group grades of evidence** **High certainty:** we are very confident that the true effect lies close to that of the estimate of the effect. **Moderate certainty:** we are moderately confident in the effect estimate: the true effect is likely to be close to the estimate of the effect, but there is a possibility that it is substantially different. **Low certainty:** our confidence in the effect estimate is limited: the true effect may be substantially different from the estimate of the effect. **Very low certainty:** we have very little confidence in the effect estimate: the true effect is likely to be substantially different from the estimate of effect. | | | | | | |

#### Explanations

a. Inadequate sequence generation/ generation of comparable groups

b. Inadequate concealment of allocation during randomization process

c. Incomplete data and/or large loss to follow up,

d. Selective outcome reporting

e. Differences between the population of interest and those studied

f. Differences between the intervention/comparator of interest and those studied. We were interested in short-term treatment (up to 4 weeks). The outcome was measured after longer term treatment.

g. Few events

h. Only data from one study

i. Low number of patients and few events

j. No events

k. Wide confidence intervals

|  | | | | | | |
| --- | --- | --- | --- | --- | --- | --- |
| **GRADE Summary of Findings Table. Quetiapine compared to placebo for short-term treatment of newly-onset symptoms of anxiety and distress** | | | | | | |
| **Patient or population:** Non-hospitalised adults with new-onset of symptoms of anxiety in need of short-term pharmacological treatment (maximum up to 4 weeks).  **Setting:** Non-hospitalised  **Intervention:** Quetiapine  **Comparison:** Placebo | | | | | | |
| Outcomes | **Anticipated absolute effects^*^** (95% CI) | | Relative effect (95% CI) | № of participants (studies) | Certainty of the evidence (GRADE) | Comments |
|  | **Risk with placebo** | **Risk with Quetiapine** |  |  |  |  |
| Anxiety symptoms  (critical outcome) assessed with: HAM-A | - | SMD **0.54 lower** (0.66 lower to 0.41 lower) | - | 1050 (3 RCTs) | ⨁⨁◯◯ Low^a,b,c^ | Short-term treatment with quetiapine may reduce symptoms of anxiety (critical outcome). |
| Serious adverse events  (critical outcome) | 2 per 1.000 | **3 more per 1.000**** (4 fewer to 10 more) | **RR 1.19** (0.12 to 11.32) | 1069 (3 RCTs) | ⨁◯◯◯ Very low^a,b,c,d,e,f^ | The evidence is very uncertain about the effect of short-term treatment with quetiapine on the number of patients with serious adverse events (critical outcome) |
| Addiction - Withdrawal symptoms | - | SMD **0.22 higher** (0.04 higher to 0.4 higher) | - | 651 (2 RCTs) | ⨁◯◯◯ Very low^a,b,d,e,g^ | The evidence is very uncertain about the effect of short-term treatment with quetiapine on addiction - measured as withdrawal symptoms. |
| Suicidal thoughts/attempts | 5 per 1.000 | **3 fewer per 1.000** (5 fewer to 35 more) | **RR 0.36** (0.02 to 8.04) | 432 (2 RCTs) | ⨁◯◯◯ Very low^a,b,c,d,f^ | The evidence is very uncertain about the effect of short-term treatment with quetiapine on the number of patients with suicidal thoughts/attempts. |
| Daytime drowsiness | 68 per 1.000 | **38 more per 1.000** (22 fewer to 180 more) | **RR 1.56** (0.67 to 3.64) | 1069 (3 RCTs) | ⨁◯◯◯ Very low^a,b,c,d,e^ | The evidence is very uncertain about the effect of short-term treatment with quetiapine on daytime drowsiness. |
| Weight change | 9 per 1.000 | **27 more per 1.000** (1 fewer to 148 more) | **RR 4.05** (0.94 to 17.44) | 637 (1 RCT) | ⨁◯◯◯ Very low^b,c,d,e,h^ | The evidence is very uncertain about the effect of short-term treatment with quetiapine on weight change. |
| Extrapyramidal symptoms | 42 per 1.000 | **22 more per 1.000** (11 fewer to 91 more) | **RR 1.52** (0.73 to 3.17) | 637 (1 RCT) | ⨁◯◯◯ Very low^b,c,d,h^ | The evidence is very uncertain about the effect of short-term treatment with quetiapine on extrapyramidal symptoms. |
| Dizziness | 77 per 1.000 | **54 more per 1.000** (12 more to 115 more) | **RR 1.70** (1.16 to 2.49) | 1069 (3 RCTs) | ⨁◯◯◯ Very low^a,b,c,d,e^ | The evidence is very uncertain about the effect of short-term treatment with quetiapine on dizziness. |
| *The risk in the intervention group (and its 95% confidence interval) is based on the assumed risk in the comparison group and the relative effect of the intervention (and its 95% CI).  ** The absolute numbers are calculated based on a risk difference analysis. | | | | | | |
| **GRADE Working Group grades of evidence** **High certainty:** we are very confident that the true effect lies close to that of the estimate of the effect. **Moderate certainty:** we are moderately confident in the effect estimate: the true effect is likely to be close to the estimate of the effect, but there is a possibility that it is substantially different. **Low certainty:** our confidence in the effect estimate is limited: the true effect may be substantially different from the estimate of the effect. **Very low certainty:** we have very little confidence in the effect estimate: the true effect is likely to be substantially different from the estimate of effect. | | | | | | |

#### Explanations

a. Inadequate concealment of allocation during randomization process

b. Incomplete data and/or large loss to follow up

c. Differences between the population of interest and those studied

d. Differences between the population of interest and those studied

e. Wide confidence intervals

f. Few events

g. Differences between the intervention/comparator of interest and those studied. We were interested in short-term treatment (up to 4 weeks). The outcome was measured after longer term treatment

h. Only data from one study

|  | | | | | | |
| --- | --- | --- | --- | --- | --- | --- |
| **GRADE Summary of Finding Table. Agomelatine compared to placebo for short-term treatment of newly-onset symptoms of anxiety and distress** | | | | | | |
| **Patient or population:** Non-hospitalised adults with new-onset of symptoms of anxiety in need of short-term pharmacological treatment (maximum up to 4 weeks).  **Setting:** Non-hospitalised  **Intervention:** Agomelatine  **Comparison:** Placebo | | | | | | |
| Outcomes | **Anticipated absolute effects^*^** (95% CI) | | Relative effect (95% CI) | № of participants (studies) | Certainty of the evidence (GRADE) | Comments |
|  | **Risk with placebo** | **Risk with Agomelatine** |  |  |  |  |
| Anxiety symptoms  (critical outcome) assessed with: HAM-A | - | SMD **0.22 lower** (0.65 lower to 0.21 higher) | - | 529 (2 RCTs) | ⨁◯◯◯ Very low^a,b,c,d,e,f,g,h,i^ | The evidence is very uncertain about the effect of short-term treatment with agomelatine on symptoms of anxiety (critical outcome) |
| Serious adverse events  (critical outcome) | 10 per 1.000 | **7 more per 1.000**** (14 fewer to 28 more) | **RR 1.84** (0.39 to 8.74) | 533 (2 RCTs) | ⨁◯◯◯ Very low^a,b,c,d,e,h,i,j,k^ | The evidence is very uncertain about the effect of short-term treatment with agomelatine on the number of patients serious adverse events (critical outcome) |
| Daytime drowsiness | 14 per 1.000 | **4 more per 1.000** (10 fewer to 78 more) | **RR 1.30** (0.25 to 6.60) | 410 (1 RCT) | ⨁◯◯◯ Very low^a,b,c,d,h,i,j,l^ | The evidence is very uncertain about the effect of short-term treatment with agomelatine on daytime drowsiness. |
| Addiction - withdrawal symptoms | - | SMD **0.06 lower** (0.42 lower to 0.29 higher) | - | 121 (1 RCT) | ⨁◯◯◯ Very low^h,i,j,l,m^ | The evidence is very uncertain about the effect of short-term treatment with agomelatine on addiction - measured as withdrawal symptoms. |
| Dizziness | 25 per 1.000 | **14 more per 1.000** (11 fewer to 89 more) | **RR 1.56** (0.54 to 4.56) | 431 (2 RCTs) | ⨁◯◯◯ Very low^a,b,c,d,e,h,i,j^ | The evidence is very uncertain about the effect of short-term treatment with agomelatine on dizziness. |
| *The risk in the intervention group (and its 95% confidence interval) is based on the assumed risk in the comparison group and the relative effect of the intervention (and its 95% CI).  ** The absolute numbers are calculated based on a risk difference analysis. | | | | | | |
| **GRADE Working Group grades of evidence** **High certainty:** we are very confident that the true effect lies close to that of the estimate of the effect. **Moderate certainty:** we are moderately confident in the effect estimate: the true effect is likely to be close to the estimate of the effect, but there is a possibility that it is substantially different. **Low certainty:** our confidence in the effect estimate is limited: the true effect may be substantially different from the estimate of the effect. **Very low certainty:** we have very little confidence in the effect estimate: the true effect is likely to be substantially different from the estimate of effect. | | | | | | |

#### Explanations

a. Inadequate sequence generation/ generation of comparable groups

b. Inadequate concealment of allocation during randomization process

c. Inadequate/lack of blinding of outcome assessors

d. Incomplete data and/or large loss to follow up

e. Selective outcome reporting

f. The magnitude of statistical heterogeneity was high

g. The direction of the effect is not consistent between the included studies

h. Differences between the population of interest and those studied.

i. Wide confidence intervals

j. Differences between the intervention/comparator of interest and those studied. We were interested in short-term treatment (up to 4 weeks). The outcome was measured after longer term treatment.

k. Few events

l. Only data from one study

m. Low number of patients

|  | | | | | | |
| --- | --- | --- | --- | --- | --- | --- |
| **GRADE Summary of Finding Table. Hydroxyzine compared to placebo for short-term treatment of newly-onset symptoms of anxiety and distress** | | | | | | |
| **Patient or population:** Non-hospitalised adults with new-onset of symptoms of anxiety in need of short-term pharmacological treatment (maximum up to 4 weeks).  **Setting:** Non-hospitalised  **Intervention:** Hydroxyzine  **Comparison:** Placebo | | | | | | |
| Outcomes | **Anticipated absolute effects^*^** (95% CI) | | Relative effect (95% CI) | № of participants (studies) | Certainty of the evidence (GRADE) | Comments |
|  | **Risk with placebo** | **Risk with Hydroxyzine** |  |  |  |  |
| Anxiety symptoms  (critical outcome) assessed with: HAM-A | - | SMD **0.21 lower** (0.48 lower to 0.06 higher) | - | 210 (1 RCT) | ⨁◯◯◯ Very low^a,b,c,d,e,f,g^ | The evidence is very uncertain about the effect of short-term treatment with hydroxyzine on symptoms of anxiety (critical outcome) |
| Serious adverse events  (critical outcome) | 0 per 1.000 | **10 more per 1.000**** (16 fewer to 35 more) | **RR 3.23** (0.13 to 78.34) | 218 (1 RCT) | ⨁◯◯◯ Very low^a,b,c,d,e,f,g,h,i^ | The evidence is very uncertain about the effect of short-term treatment with hydroxyzine on the number of patients serious adverse events (critical outcome) |
| Daytime drowsiness | 18 per 1.000 | **21 more per 1.000** (11 fewer to 189 more) | **RR 2.15** (0.40 to 11.51) | 218 (1 RCT) | ⨁◯◯◯ Very low^a,b,c,d,e,f,g,h^ | The evidence is very uncertain about the effect of short-term treatment with hydroxyzine on daytime drowsiness. |
| *The risk in the intervention group (and its 95% confidence interval) is based on the assumed risk in the comparison group and the relative effect of the intervention (and its 95% CI).  ** The absolute numbers are calculated based on a risk difference analysis.  **Abbreviation: CI:** Confidence interval; **GRADE**: Grades of Recommendation, Assessment, Development and Evaluation; **SMD:** Standardised mean difference; **RR:** Risk ratio; **RD:** risk difference | | | | | | |
| **GRADE Working Group grades of evidence** **High certainty:** we are very confident that the true effect lies close to that of the estimate of the effect. **Moderate certainty:** we are moderately confident in the effect estimate: the true effect is likely to be close to the estimate of the effect, but there is a possibility that it is substantially different. **Low certainty:** our confidence in the effect estimate is limited: the true effect may be substantially different from the estimate of the effect. **Very low certainty:** we have very little confidence in the effect estimate: the true effect is likely to be substantially different from the estimate of effect. | | | | | | |

#### Explanations

a. Inadequate sequence generation/ generation of comparable groups,

b. Inadequate concealment of allocation during randomization process

c. Incomplete data and/or large loss to follow up

d. Selective outcome reporting

e. Differences between the population of interest and those studied

f. Wide confidence intervals

g. Only data from one study

h. Differences between the intervention/comparator of interest and those studied. We were interested in short-term treatment (up to 4 weeks). The outcome was measured after longer term treatment

i. Few events

|  | | | | | | |
| --- | --- | --- | --- | --- | --- | --- |
| **GRADE Summary of Finding Table. Benzodiazepine compared to pregabalin for short-term treatment of newly-onset symptoms of anxiety and distress** | | | | | | |
| **Patient or population:** Non-hospitalised adults with new-onset of symptoms of anxiety in need of short-term pharmacological treatment (maximum up to 4 weeks).  **Setting:** Non-hospitalised  **Intervention:** Benzodiazepine  **Comparison:** Pregabalin | | | | | | |
| Outcomes | **Anticipated absolute effects^*^** (95% CI) | | Relative effect (95% CI) | № of participants (studies) | Certainty of the evidence (GRADE) | Comments |
|  | **Risk with Pregabalin** | **Risk with Benzodiazepine** |  |  |  |  |
| Anxiety symptoms (critical outcome) assessed with: HAM-A | - | SMD **0.04 higher** (0.4 lower to 0.49 higher) | - | 879 (4 RCTs) | ⨁◯◯◯ Very low^a,b,c,d,e,f^ | The evidence is very uncertain about the effect of short-term treatment with benzodiazepine on symptoms of anxiety compared to pregabalin (critical outcome). |
| Serious adverse events (critical outcome) | 0 per 1.000 | **13 more per 1.000**** (5 fewer to 31 more) | **RR 6.79** (1.08 to 42.82) | 774 (3 RCTs) | ⨁◯◯◯ Very low^a,b,c,d,e,f,g,h^ | The evidence is very uncertain about the effect of short-term treatment with benzodiazepine on serious adverse events compared to pregabalin (critical outcome). |
| Addiction - wihtdrawal symptoms | - | SMD **0.13 higher** (0.05 lower to 0.3 higher) | - | 819 (4 RCTs) | ⨁◯◯◯ Very low^a,b,c,d,e,f,g^ | The evidence is very uncertain about the effect of short-term treatment with benzodiazepine on addiction - wihtdrawal symptom compared to pregabalin |
| Suicidal thoughts/attempts | 0 per 1.000 | **0 more per 1.000**** (23 fewer to 23 more) | **RD 0.00** (-0.023 to 0.023) | 204 (1 RCT) | ⨁◯◯◯ Very low^a,b,c,d,e,g,i,j^ | The evidence is very uncertain about the effect of short-term treatment with benzodiazepine on suicidal thoughts/attempts compared to pregabalin |
| Daytime drowsiness | 360 per 1.000 | **191 more per 1.000** (25 more to 425 more) | **RR 1.53** (1.07 to 2.18) | 774 (3 RCTs) | ⨁◯◯◯ Very low^a,b,c,d,e,f,g,k^ | The evidence is very uncertain about the effect of short-term treatment with benzodiazepine on daytime drowziness compared to pregabalin |
| Cardiac side-effects risk difference | 0 per 1.000 | **0 more per 1.000**** (16 fewer to 16 more) | **RD 0.00** (-0.016 to 0.016) | 363 (1 RCT) | ⨁◯◯◯ Very low^a,b,c,d,e,g,i,j^ | The evidence is very uncertain about the effect of short-term treatment with benzodiazepine on cardiac side-effects compared to pregabalin |
| Dizziness | 321 per 1.000 | **141 fewer per 1.000** (221 fewer to 6 fewer) | **RR 0.56** (0.31 to 0.98) | 774 (3 RCTs) | ⨁◯◯◯ Very low^a,b,c,d,e,f,g^ | The evidence is very uncertain about the effect of short-term treatment with benzodiazepine on dizziness compared to pregabalin |
| *The risk in the intervention group (and its 95% confidence interval) is based on the assumed risk in the comparison group and the relative effect of the intervention (and its 95% CI).  ** The estimate and absolute numbers are calculated based on a risk difference analysis.  **Abbreviation: CI:** Confidence interval; **GRADE**: Grades of Recommendation, Assessment, Development and Evaluation; **SMD:** Standardised mean difference; **RR:** Risk ratio; **RD:** risk difference | | | | | | |
| **GRADE Working Group grades of evidence** **High certainty:** we are very confident that the true effect lies close to that of the estimate of the effect. **Moderate certainty:** we are moderately confident in the effect estimate: the true effect is likely to be close to the estimate of the effect, but there is a possibility that it is substantially different. **Low certainty:** our confidence in the effect estimate is limited: the true effect may be substantially different from the estimate of the effect. **Very low certainty:** we have very little confidence in the effect estimate: the true effect is likely to be substantially different from the estimate of effect. | | | | | | |

#### Explanations

a. Inadequate sequence generation/ generation of comparable groups

b. Inadequate concealment of allocation during randomization process,

c. Incomplete data and/or large loss to follow up

d. Selective outcome reporting

e. Differences between the population of interest and those studied.

f. Wide confidence intervals

g. Differences between the intervention/comparator of interest and those studied. We were interested in short-term treatment (up to 4 weeks). The outcome was measured after longer term treatment.

h. Few events

i. Only data from one study.

j. Low number of patients and no events

k. The direction of the effect is not consistent between the included studies
